# HIV-Exposed Seronegative Sex Workers Express Low T-Cell Activation and an Intact Ectocervical Tissue Microenvironment

**DOI:** 10.3390/vaccines9030217

**Published:** 2021-03-04

**Authors:** Maria Röhl, Annelie Tjernlund, Julie Lajoie, Gabriella Edfeldt, Frideborg Bradley, Sofia Bergström, Vilde Kaldhusdal, Alexandra Åhlberg, Anna Månberg, Kenneth Omollo, Geneviève Boily-Larouche, Muhammad Asghar, Douglas S. Kwon, Julius Oyugi, Joshua Kimani, Peter Nilsson, Keith R. Fowke, Kristina Broliden

**Affiliations:** 1 Center for Molecular Medicine, Department of Medicine Solna, Division of Infectious Diseases, Karolinska Institutet, Department of Infectious Diseases, Karolinska University Hospital, 17164 Stockholm, Sweden; maria.rohl@ki.se (M.R.); annelie.tjernlund@ki.se (A.T.); gabriella.edfeldt@ki.se (G.E.); frideborg.bradley@ki.se (F.B.); vilde.kaldhusdal@ki.se (V.K.); alexandra.ahlberg@ki.se (A.Å.); asghar.muhammad@ki.se (M.A.); 2 Department of Medical Microbiology, University of Nairobi, 00100 Nairobi, Kenya; julie.lajoie1@gmail.com (J.L.); oduorkenneth@gmail.com (K.O.); julias.oyugi9@gmail.com (J.O.); jkimani@csrtkenya.org (J.K.); keith.fowke@umanitoba.ca (K.R.F.); 3 Department of Medical Microbiology and Infectious Diseases, University of Manitoba, Winnipeg, MB R3A 1R9, Canada; genevieve.boily.larouche@gmail.com; 4 Division of Affinity Proteomics, Department of Protein Science, SciLifeLab, KTH-Royal Institute of Technology, 17164 Stockholm, Sweden; sofia.bergstrom@scilifelab.se (S.B.); anna.manberg@scilifelab.se (A.M.); peter.nilsson@scilifelab.se (P.N.); 5 Ragon Institute of MGH, MIT, and Harvard, Cambridge, MA 02139, USA; dkwon@mgh.harvard.edu; 6 Partners for Health and Development in Africa, 00100 Nairobi, Kenya; 7 Department of Community Health Sciences, University of Manitoba, Winnipeg, MB R3A 1R9, Canada

**Keywords:** HIV, mucosa, female genital tract, RNA sequencing, microbiome, image analysis, protein profiling, HIV-exposed seronegative, HESN

## Abstract

Immunological correlates of natural resistance to HIV have been identified in HIV-exposed seronegative (HESN) individuals and include a low-inflammatory genital mucosal status. The cervicovaginal epithelium has not been studied for such correlates despite constituting an important barrier against sexual HIV transmission. To fill this gap in knowledge, we collected samples of blood, cervical mononuclear cells, cervicovaginal lavage, and ectocervical tissue from Kenyan HESN sex workers (n = 29) and controls (n = 33). The samples were analyzed by flow cytometry, protein profiling, 16S rRNA gene sequencing, in situ image analysis, and tissue-based RNA sequencing. A significantly higher relative proportion of regulatory T cells in blood (B7^+^CD25^hi^FoxP3^+^CD127^lo^CD4^+^ and B7^+^Helios^+^FoxP3^+^CD4^+^), and a significantly lower proportion of activated cervical T cells (CCR5^+^CD69^+^CD4^+^ and CCR5^+^CD69^+^CD8^+^), were found in the HESN group compared with the controls. In contrast, there were no statistically significant differences between the study groups in cervicovaginal protein and microbiome compositions, ectocervical epithelial thickness, E-cadherin expression, HIV receptor expression, and tissue RNA transcriptional profiles. The identification of an intact ectocervical microenvironment in HESN individuals add new data to current knowledge about natural resistance to sexual transmission of HIV.

## 1. Introduction

Globally, sexual HIV-1 (HIV) transmission is the most common infection route and in high-prevalence settings women remain at high risk of acquiring HIV [1]. There is substantial inter-individual heterogeneity in susceptibility to HIV infection and this phenomenon has been studied in highly HIV-exposed, yet seronegative, individuals to find correlates of protective mucosal immune responses. Such high-risk subjects are referred to as HIV-exposed seronegative (HESN) individuals and include sex workers, HIV-status discordant couples, intravenous drug users, and children born to untreated HIV-infected mothers. Relative resistance to HIV infection is likely multi-factorial and several studies have identified innate immune and anti-inflammatory factors, HIV-specific T- and B-cell responses, increased regulatory T- and B-cell activity as well as genetic and epigenetic correlates to this phenotype [2,3,4,5,6,7,8,9,10,11,12,13,14,15].

HESN women have been clearly identified among the Pumwani Sex Worker Cohort in Nairobi, Kenya [16]. Proposed immune correlates in this cohort include many of the immune factors mentioned above [2,3,8,9]. Overall, the results in this sex-worker cohort indicate a HESN-associated state of general low immune activation termed “immune quiescence” [3]. This phenomenon was partly explained by their elevated frequencies of classical CD4^+^CD25^+^FoxP3^+^ regulatory T cells (Tregs) in blood, reduced frequencies of blood and cervical mononuclear cell (CMC)-derived T cells expressing the activation marker CD69, and an altered cytokine profile as compared with HIV-seronegative control women [2,9].

These and other studies on mucosal immune correlates of protection against HIV have been defined by assessing peripheral blood mononuclear cells (PBMCs), plasma samples, cervicovaginal secretions, as well as cytobrush-derived CMCs. Although such samples allow characterization of important viral defense mechanisms, they do not fully represent how primary HIV infection can be hindered within the genital epithelium and submucosa. To explore the genital epithelium at a molecular level it is necessary to obtain tissue biopsies which can be logistically and ethically challenging in a high-risk population [17,18]. Here, we addressed a gap in knowledge of mucosal immunology of HESN sex-working women by assessing unique ectocervical tissue samples from the Pumwani Sex Worker Cohort. The ectocervical epithelium is structurally similar to the vaginal epithelium. Together, these barriers cover the vaginal vault and is therefore a likely site of sexual HIV transmission. The epithelial structure and immune cell content of this important barrier could thus, theoretically, be important mucosal immune correlates of protection against HIV. However, while we here identified other mucosal immune correlates of protection in corresponding PBMC and mucosal samples from the HESN individuals, the ectocervical tissue microenvironment did not differ from the control group as assessed by tissue transcriptomic profiling or image analysis of the epithelial structure and HIV target cell distribution.

## 2. Materials and Methods

### 2.1. Study Participants

This study involved HIV-seronegative women from the Pumwani Sex Worker Cohort, Nairobi, Kenya, who were divided into two study groups depending on the length of sex work practice. The HESN phenotype was here defined as study subjects who were involved in self-declared sex work for at least 7 years. Study subjects active in self-declared sex work for 3 years or less were here controls and were referred to as new negatives (NN). This project is a sub-study of a longitudinal study with previously defined inclusion and exclusion criteria [18]. Participants answered a demographic and behavioral questionnaire at enrolment and at subsequent study visits. Briefly, the study subjects were in general good health. Inclusion criteria at enrolment of this sub-study were: active in sex work, age 18 to 50 years, not being menopausal, having a regular menstrual cycle, no hormonal contraceptive use, no prior hysterectomy, not pregnant or breastfeeding, and no clinical or laboratory signs of ongoing genital infections or seropositivity for HIV or syphilis. Plasma samples were used for HIV-serology, using a rapid test (Determine, Inverness Medical, Shinjuku-ku, Japan), and for *Treponema pallidum* serology (Macro-Vue Rapid Plasma Reagin test, Becton Dickinson, Franklin Lakes, NJ, USA). Urine samples were collected for PCR detection of *Neisseria gonorrhea* and *Chlamydia trachomatis* using the Roche AMPLICOR kit (Pleasanton, NJ, USA). Presence of *Trichomonas vaginalis* was diagnosed using normal saline microscopy and bacterial vaginosis (BV) was defined with Nugent’s Score of Gram-stained smears. Furthermore, measurement of progesterone (P4) concentration in plasma was performed at the first sample time point using the MILLIPLEX MAP Steroid/Thyroid Hormone Magnetic Bead Panel (Millipore, Merck, Darmstadt, Germany) with the sensitivity of 0.25 ng/mL (standard curve range 0.14–100 ng/mL). Written informed consent was obtained from all participants prior to enrolment in the study. The ethical review boards from the Kenyatta National Hospital/University of Nairobi (#P211/09/2006, renewed and amended annually since 2006), the University of Manitoba (HS15280 (B2012:043, regularly renewed since 2012), and the Regional Ethical Review Board in Stockholm (2014/959-31, amended in 2018: 2018/1306-31) approved the study.

### 2.2. Sample Collection

Samples for the present study were collected at two time points from each participant. The first sample time point (all sample sources) aimed for the luteal phase of the menstrual cycle as determined by days since the onset of last menses. The second sample time point (only tissue samples) was approximately two weeks later which is more representative of the follicular phase of the menstrual cycle. Blood samples were collected through venipuncture with sodium heparin as an anticoagulant. PBMCs were isolated by Ficoll-Paque (VWR, Mississauga, ON, Canada) density gradient. Cervicovaginal lavages (CVL) were collected by washing the endocervical os with 2 mL of sterile PBS. The PBS was then collected from the posterior fornix into a 15 mL conical tube and put on ice before transportation. At arrival in the laboratory, the tubes were centrifuged to separate the supernatant from the cellular debris, the latter referred to here as “CVL pellet,” and the specimens were stored at −80 °C. CMCs were collected under speculum examination using a cytobrush and plastic scraper and put on ice until being freshly processed at the laboratory. Tissue samples included two ectocervical biopsies from the superior part of the ectocervix collected using Schubert biopsy forceps (model ER058R; Aesculap, Tuttlingen, Germany) by a trained gynecologist [18]. One biopsy was immediately snap frozen in liquid nitrogen and stored at −80 °C; the other biopsy was immediately placed in RNAlater solution and stored at −80 °C.

Participants were asked to abstain from vaginal intercourse for two weeks following the tissue sampling in line with a previous mucosal sample collection protocol [18]. Testing for prostate-specific antigen (PSA) in genital secretions was performed using a rapid test kit (Seratec PSA Semiquant, Göttingen, Germany) at three days after each biopsy sampling and two weeks later to assess sexual abstinence.

### 2.3. Phenotyping of Peripheral Blood Mononuclear Cells and Cervical Mononuclear Cells

Phenotyping of PBMCs and CMCs by flow cytometry was performed to investigate immune cell populations and activation profiles [19]. For each participant, 10^6^ PBMCs and whole CMC pellet were immunophenotyped ex vivo. Fresh cells were stained with Red-Live-Dead discriminant dye (Invitrogen, Carlsbad, CA, USA) to identify viable cells.

Next, cells were stained for the following markers: PBMC Tregs: CD3-V500 (561416), CD25-PE CF594 (562403), CD45RA-Alexa700 (560774), CD127-PeCy7 (560822) (BD Biosciences, San Jose, CA, USA); CD4-APCC7 (344616), B7-FITC (321214), CD39-BV421 (328213) (Biolegend, San Diego, CA, USA); Helios-Alexa647 (51-9883-82), FoxP3-PE (555854) (ebioscience, Santa Clara, CA, USA). CMC activated CD4 and CD8 cells: CD3-PECy5 (555334), CD4-FITC (555346), CD8-V500 (560774), CD69-PeCy7 (560774), CCR5-V450 (562381) (BD Biosciences). Gating of T cells in PBMC and CMC have been described in detail [19] (Figure S1). Briefly, it was done as follows: (I) singlet; (II) lymphocyte gating; (III) CD3^+^ live; (IV) CD4^+^/CD8^+^; (V) out of CD4^+^ or CD8^+^ (parent population for specific CD4^+^ or CD8^+^ T cells) gating the different markers of PBMC and CMC populations were defined.

To ensure the quality of the data, CMC samples that did not reach 100 live CD3^+^ cells were excluded from the analyses. Data that passed normality test were analyzed with unpaired T-test, data that did not pass were analyzed with the Mann–Whitney *U* test to compare the two study groups. Statistical analyses were performed using GraphPad Prism 6 (version 6.0, GraphPad Software). All *p*-values were two-sided and values < 0.05 were considered significant.

### 2.4. Protein Profiling Using a Bead-Based Affinity Assay

Protein targets were selected from a larger panel of proteins with verified presence in genital fluids and with associations to HIV resistance and inflammation [20]. The panel was further supplemented with protein targets identified in studies on the effect of sex hormones and inflammation on the female genital tract [21,22,23,24,25]. In total, 74 proteins were selected and the used antibody set was polyclonal rabbit antibodies generated within the Human Protein Atlas (HPA) project [26] (Table S1). In brief, previously reported protocols for cervicovaginal secretions were here utilized for CVL samples [20]. Antibodies were coupled to color-coded magnetic beads (MagPlex-C, Luminex Corp., Austin, TX, USA) using EDC-NHS chemistry and mixed to form a bead array. Samples were distributed in 96-well microtiter plates based on age, group, and sampling time point. The protein content of the samples was labeled with biotin and subsequently further diluted prior to heat treatment. The bead array and the heat-treated samples were combined in a 384-well microtiter plate and incubated over night at room temperature. A streptavidin-conjugated fluorophore (Streptavidine R-Phycoerythrin Conjugate, Invitrogen, Stockholm, Sweden) was added to enable detection of captured proteins. Detection for each sample was performed using a FlexMap 3D instrument (Luminex Corp., Austin, TX, USA), where binding events were displayed as fluorescence intensity (arbitrary unit) per sample and bead identity. Data processing was performed using the open software R (version 3.6) [27]. The data was log-transformed and normalized in order to reduce the differences between labeling plates and with robust linear regression applied over sample plate position (rlm function, R package MASS) in order to diminish the effect of delay time during read-out. The potential differences in protein levels in CVL between the two sample groups were evaluated using Mann–Whitney *U* test where *p*-values < 0.05 were regarded significant.

### 2.5. Cytokine Measurement

Levels of a panel of 14 pre-selected cytokines were measured in CVL samples by Milliplex MAP kit (Millipore, Billerica, MA, USA) and analyzed on a BioPlex-200 (Bio-Rad, Mississauga, ON, Canada). The samples were assayed using an overnight incubation protocol [9]. The cytokine expression (values given as pg/mL) showed a skewed distribution and were therefore log_10_ transformed for further analysis. After log transformation, normality test was performed and if the data passed, an unpaired T test was used (IL-1α, IL-1β, IP-10, MCP-1, MIP-1α, MIP-1β, IL-1Ra, MIG, and MIP-3α). For the other markers, the Mann–Whitney *U* test was used. Statistical analyses were performed using GraphPad Prism 6 (version 6.0, GraphPad Software). All *p*-values were two-sided and *p*-values < 0.05 were regarded significant.

### 2.6. 16S Ribosomal RNA (rRNA) Gene Sequencing to Determine the Cervicovaginal Microbiome Composition

Nucleic acid was extracted using the PowerFecal DNA isolation kit (MO BIO Laboratories Inc., Carlsbad, CA, USA) (HESN, n = 24, NN = 26), or a phenol-chloroform-isopropanol protocol [28] (HESN, n = 3, NN = 4) from the CVL pellet. The V4 region of the 16S rRNA gene was amplified and sequenced using Illumina MiSeq instrument [28]. DADA2 [29] was used to create amplicon sequence variant (ASV) table and assign taxonomy with the RDP database [30]. The ASVs with missing annotation on genus level were blasted with nucleotide BLAST [31] against the Silva database version 32 [32]. The dataset contained 382 unique ASVs, all but 18 of these were annotated down to genus level resulting in 64 unique taxa at genus level. Genus level taxonomy was used for all but *Lactobacillus* genus that was separated in *Lactobacillus iners* and non-*iners*.

Based on this taxonomy, the study participants were assigned to one of four cervicotype (CT) groups as previously described [33]. All statistical analysis, data processing, and visualization were performed in R version 3.6.0 (R Core Team, 2018). Scripts were written using many of the packages included in the tidyverse version 1.3.0 [34]. The ggplot2 package version 3.3.1 [35] was used for data visualization. The phyloseq package version 1.30.0 [36] was used to create the ordination plot. Pair-wise comparisons between cervicotype groups were tested using the two-sided Mann–Whitney *U* test. Correlation analysis was performed using Spearman’s correlation.

### 2.7. In Situ Fluorescence Staining and Image Analysis

To investigate the thickness of the epithelial layers as well as abundance and spatial localization of pre-selected CD4^+^ T-cell subsets, immunofluorescence staining was performed on 8-µm thick sections of the cryopreserved ectocervical biopsies as previously described [37]. The following primary antibodies were used: rabbit anti-human CD4 antibody (EPR6855, Abcam, Cambridge, UK), mouse anti-human E-cadherin antibody (36/E-cadherin, BD Biosciences, San Jose, CA, USA), mouse anti-human CCR5 antibody (MC-5, provided by Professor M. Mack, University Clinic of Regensburg, Germany), and rat anti-human Langerin antibody (929F3.01, Dendritics, Lyon, France). The following secondary antibodies were used: Alexa Fluor 594-conjugated donkey anti-rabbit IgG antibody, Alexa Fluor 488-conjugated donkey anti-mouse IgG antibody, and Alexa Fluor 488-conjugated donkey anti-rat IgG antibody (Molecular Probes, Life Technologies Europe BV, Stockholm, Sweden). Tissue sections were counterstained with 4′,6-Diamidino-2-phenylindole DAPI (Molecular Probes, Life Technologies). The stained tissue sections were scanned into digital images using a Pannoramic 250 Flash Slide Scanner (3DHistec Ltd., Budapest, Hungary).

The epithelium was manually outlined in regions of interest (ROIs) in Pannoramic viewer (version 1.15.3, 3DHistotech Ltd., Budapest, Hungary) on all images. The ROIs were exported as tif images which were uploaded to CellProfiler (version 2.2.0) [38,39] and a customized digital image analysis was performed. The ectocervical epithelial thickness of the total, and of the E-cadherin positive and negative layers, was measured. In detail, lines were manually drawn to indicate the basal membrane as well as the E-cadherin positive and negative layers of the epithelium. A distance transform algorithm was used to measure the distance between the manually drawn lines, and to segment the epithelium into 40–50 µm segments starting from the apical border. The expression levels and spatial localization of potential HIV target cells were assessed by measuring the expression of CD4 alone or combined with either CCR5 or Langerin. Pixel-based segmentation, suitable for immune cells with irregular cell morphologies [37,40], was used together with an image-dependent intensity threshold to segment out the positively stained area for each marker. The percentage of positively stained area within the total tissue area was used to represent the frequency of positive cells. The Mann–Whitney *U* test was used to assess the statistical significance between the two study groups.

### 2.8. RNA Sequencing (RNA-seq) Analysis

The ectocervical biopsies that were stored in RNA-later were thawed and RNA was isolated and purified using AllPrep DNA/RNA Mini Kit (QIAGEN, Hilden, Germany) and the QIAcube Connect (QIAGEN). Poly-A containing mRNA transcripts were purified, fragmented, and converted to cDNA. The cDNA fragments were amplified, barcoded, and sequenced using NextSeq 550 (Illumina, San Diego, CA, USA). Base calling and demultiplexing of the Bcl2 files was performed using the bcl2fastq program, and indexing of the human reference genome (hg38/GRCh38) was done using STAR (Spliced Transcripts Alignment to a Reference) [41]. The resulting fastq files were aligned and uniquely mapped reads were counted and mapped to annotated exons using data from UCSC (University of California Santa Cruz, Santa Cruz, CA, USA) genome browser (http://genome.ucsc.edu/ (accessed on 15 February 2021)) as reference. Alignment of the reads to the reference genome was performed in R v. 3.6.0 (R Core Team, 2018)/Bioconductor v. 3.9 (BiocManager 1.30.4) using the STAR alignment program, and read counts normalized with the edgeR package in Bioconductor. If a gene showed >1 count per million in at least three samples, that gene was included.

Analysis of differentially expressed genes (DEGs) between the HESN and NN groups was performed using the linear models pipeline in the EdgeR package. Genes with a false discovery rate (FDR) adjusted *p*-value < 0.05 were considered significant. Volcano plots of all identified genes were performed using the ggplot2 function package version 3.3.1 in [35]. All analyses were conducted using R v. 3.6.0 (R Core Team, 2018) and Bioconductor v. 3.9 (BiocManager 1.30.4). Processed data files and transcriptomics meta data are available at Gene Expression Omnibus (GEO) under accession number GSE165132.

## 3. Results

### 3.1. Study Population

Blood and mucosal samples were collected from 29 HESN women and 33 control women (NN). The HESN study participants had been active in sex work for a median of 10 yrs (range 7–31), and the NN control participants for a median of 2 yrs (range 0.25–3). At enrolment, two weeks before the first sample time point, the study groups were comparable (*p* > 0.05) for years since last pregnancy, BV status, yeast infection, number of clients the last seven days, number of unprotected sex acts with clients the last seven days, and vaginal douching (Table 1). A significant difference between the study groups were, however, observed for age (HESN: median 37 yrs vs. NN: median 31 yrs; *p* = 0.001).

The first sample time point was aimed for the luteal phase of the menstrual cycle based on self-reported menstrual staging (number of days since initiation of last menses: median: 21, range 6–31). Measurement of plasma progesterone levels confirmed the staging in 43 out of 62 study subjects (>1.2 ng/mL) progesterone. There was no statistical difference between the study groups for progesterone levels (Table 1). All experimental assays were performed on samples from the first sample time point, except for the transcriptomic profiling for which RNAlater-preserved samples were only available at the second sample time point (two weeks after the first sample time point and four weeks after enrolment). The image analyses were performed at the first sample time point and verified for the major markers CD4 and E-cadherin at the second sample time point as described below.

### 3.2. Higher Relative Proportion of Regulatory T Cells (Treg) in Blood in HESN versus NN Women

**Figure 1:**
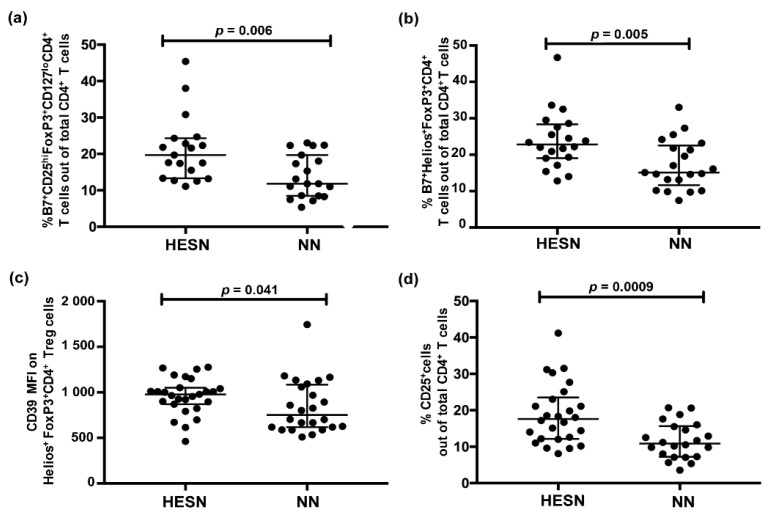
Expression of regulatory T cell populations in PBMC samples from HESN and NN women as assessed by flow cytometry. (**a**) Relative proportion of B7^+^CD25^hi^FoxP3^+^CD127^low^CD4^+^ T cells out of total CD4^+^ T cells. (**b**) Relative proportion of B7^+^ Helios^+^ FoxP3^+^CD4^+^ T cells out of total CD4^+^ T cells. (**c**) MFI of CD39 on Helios^+^FoxP3^+^CD4^+^ T cells. (**d**) Expression of CD25 on CD4^+^ T cells. Data are presented as median and interquartile range. Data were analyzed with the Mann–Whitney *U* test and *p*-values < 0.05 were considered significant. Treg: regulatory T cells; FoxP3: Forkhead box P3; MFI: mean fluorescence intensity.

We assessed, by flow cytometry, phenotypic markers for Tregs in PBMC samples of the study participants corresponding to their first sample time point. The Treg marker CD25 (the IL-2 receptor α-chain) and the transcription factor FoxP3 were here complemented with a larger panel of phenotypical markers to better characterize the functional properties of the CD4^+^ Tregs. The extended panel included B7, a subcomponent of the integrin α4β7, which is associated with an improved capacity to induce suppressive T cells and to migrate to mucosal tissue [42]. The flow cytometry panel also included Helios, a transcription factor often expressed by Tregs. The HESN group had higher relative proportion of the Treg population defined by B7^+^CD25^hi^FoxP3^+^CD127^lo^CD4^+^ T cells out of the total CD4^+^ T cells compared to the NN group (HESN, n = 19: median 20%, IQ range 13–24; NN, n = 19: median 12%, IQ range 8–20); *p* = 0.006) (Figure 1a). Furthermore, the HESN group showed higher relative proportion of Helios^+^FoxP3^+^CD4^+^ T cells expressing B7 (B7^+^Helios^+^FoxP3^+^CD4^+^) out of the total CD4^+^ T cells (HESN: n = 20, median 23%, IQ range 19–28; NN: n = 21, median 15%, IQ range 12–22; *p* = 0.005) (Figure 1b). The level of expression of CD39, a marker for regulation of inflammation, on Helios^+^FoxP3^+^CD4^+^ Tregs was higher in HESN women (HESN: n = 27, median fluorescence intensity (MFI) 978, IQ range 869–1051; NN: n = 24, MFI 751, IQ range 617–1085; *p* = 0.041) (Figure 1c). HESN women also showed a higher percentage of CD25 out of total CD4^+^ T cells (CD25^+^CD4^+^) (HESN: n = 26, median 18%, IQ range 12–24; NN: n = 22, median 11%, IQ range 7–16; *p* = 0.0009) (Figure 1d). Using the univariate regression analysis, age had no influence on any of the parameters outlined in Figure 1a–d within the respective study group (*p* > 0.05 for all comparisons).

### 3.3. Lower Levels of Activated Cervicovaginal CD4^+^ and CD8^+^ T Cells in HESN versus NN Women

**Figure 2:**
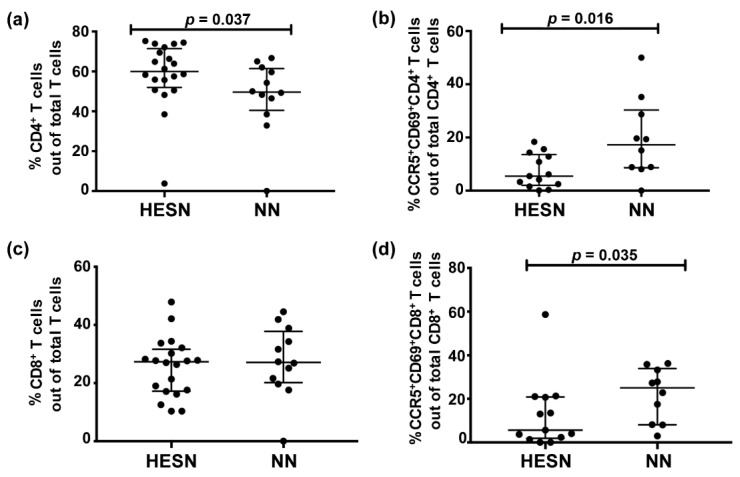
Relative proportion of cellular markers of activation on CMCs from HESN and NN study participants as assessed by flow cytometry. (**a**) Relative proportion of CD4^+^ cells out of CD3^+^ T cells; (**b**) relative proportion of CCR5^+^CD69^+^CD4^+^ T cells out of CD4^+^ T cells. (**c**) Relative proportion of CD8^+^ T cells out of CD3^+^ T cells; (**d**) relative proportion of CCR5^+^CD69^+^ CD8^+^ T cells out of CD8^+^ T cells. Data were analyzed with the Mann–Whitney *U* test and *p*-values < 0.05 were considered significant. Data are presented as median and interquartile range.

CD4^+^ and CD8^+^ T cells from CMCs were analyzed by flow cytometry. CMC samples with >100 live CD3^+^ cells were included, restricting the study to analysis of 20 HESN and 11 NN individuals (Table S2). Due to low numbers of CMCs it was not possible to include markers for Tregs. The HESN group had higher relative proportion of CD4^+^ T cells out of the CD3^+^ T cells than the NN group (HESN: median 60%, IQ range 52–71; NN: median 50%, IQ range 40–61; *p* = 0.037) (Figure 2a). However, the relative proportion of activated (CCR5^+^CD69^+^) CD4^+^ T cells, out of the total CD4^+^ T cells, was lower in the HESN group (HESN, n = 13: median 5%, IQ range 2–13; NN, n = 10: median 17%, IQ range 9–30; *p* = 0.016) (Figure 2b). There was no difference in the proportion of CD8^+^ T cells out of CD3^+^ T cells between the study groups (Figure 2c). However, the HESN group showed a lower proportion of CCR5^+^CD69^+^CD8^+^ T cells out of total CD8^+^ T cells (HESN, n = 13: median 6%, IQ range 2–21; NN, n = 10: median 25%, IQ range 8–34; *p* = 0.035) (Figure 2d). Using the univariate regression analysis, age had no influence on any of the parameters outlined within the respective study group (*p* > 0.05 for all comparisons in Figure 2a,b,d).

### 3.4. Comparable Protein Levels in CVL between HESN and NN Women

A targeted multiplex protein profiling assay was used to screen for markers of mucosal integrity and inflammation in CVL samples from the first sample time point. A total of 46 samples from the HESN (n = 20) and NN (n = 26) study subjects were utilized for these analyses. However, none of the 74 included proteins were here found to provide significantly different levels between the study groups (*p* > 0.05, Mann–Whitney *U* test) (Figure S2).

**Figure 3:**
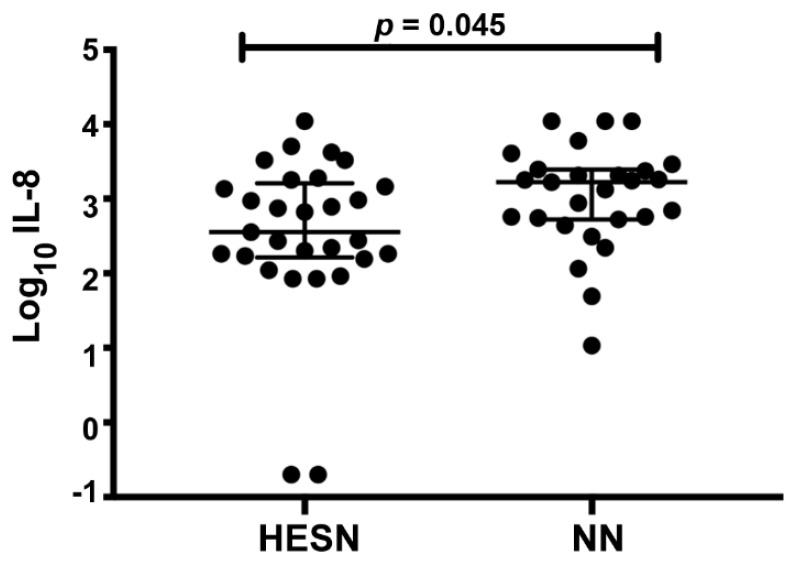
Cytokine measurement in CVL of HESN and NN study subjects. Expression of IL-8 (Log_10_ of pg/mL) with data presented as median and interquartile range. Data were analyzed with the Mann–Whitney *U* test and *p*-values < 0.05 were considered significant. CVL: cervicovaginal lavage.

The protein profiling assay was complemented by a cytokine bead-array immunoassay targeting a total of 14 pre-selected cytokines in CVL (IL-1α, IL-1Ra, IL-1β, IL-8, IL-10, IL-17, IFNγ, TNFα, MCP-1, MIP-1α, MIP-1β, IP-10, MIG, and MIP3α). The HESN (n = 29) and NN (n = 27) study subjects had comparable levels of all cytokines (Figure S3), except for the HESN group having significantly lower levels of IL-8 in a univariate analysis (HESN: median 2.55, IQ range 2.21–3.21; NN: median 3.22, IQ range 2.72–3.39; *p* = 0.045) (Figure 3). Using the univariate regression analysis, age had no influence on this parameter within the respective study group (*p* > 0.05). Furthermore, there was no statistically different number of samples that were above the detection limit when comparing the two study groups for all cytokines (Figure 3, Figure S3).

### 3.5. Comparable Cervicovaginal Microbiome Composition in HESN and NN Women

**Figure 4:**
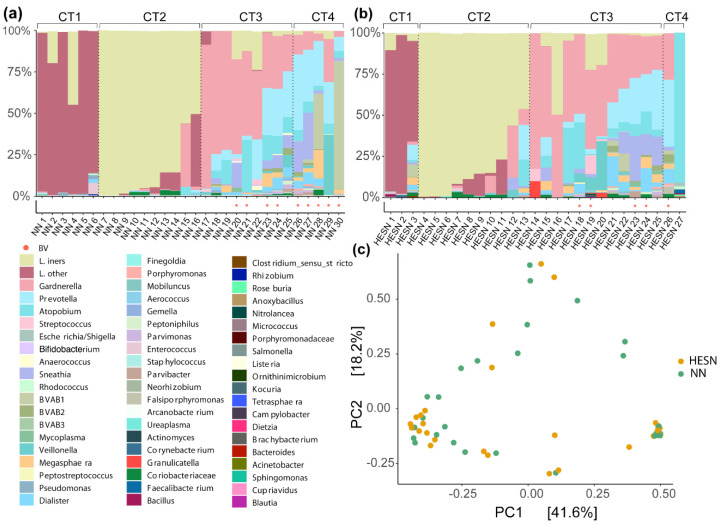
The cervicovaginal microbiome composition in CVL of HESN and NN study participants. (**a**,**b**) The microbiome composition of each sample was analyzed by sequencing of the 16S rRNA variable region 4 and grouped into four cervicotype groups (CT1-4). Each sample is represented by a bar and the relative abundance of each taxa is indicated by color codes. (**c**) A PCoA (Bray-Curtis) was constructed and each dot represents one CVL sample. CVL: cervicovaginal lavage; PCoA: Principal coordinate analysis.

There was no statistically significant difference in BV prevalence between HESN women and controls (BV positive: 21% vs. 28%) (Table 1). To complement this data, the women were grouped based on microbial community composition, as assessed by 16S rRNA gene sequencing. Microbiome composition was measured in DNA extracted from CVL pellets from the first sample time point. Although a few samples lacked material, a total of 27 HESN and 30 NN samples were successfully sequenced with a median gene count of 50,790 (range 10,490–117,183 counts). The samples were divided into four cervicotype (CT) groups based on the microbiome composition: *Lactobacillus* non-*iners* dominant, i.e., mainly *Lactobacillus crispatus* dominated, (CT1), *Lactobacillus iners* dominant (CT2), *Gardnerella* dominant (CT3), and high diverse microbiome (CT4) [33]. No statistically significant differences in the frequencies of CT1-4 were found between the study groups (CT1: HESN:11%, NN:20%; CT2: HESN:37%, NN:33%; CT3: HESN:44%, NN:30%; CT4: HESN:7%, NN:17%; *p* > 0.05 for all pair-wise comparisons using the Mann–Whitney U test). The microbial composition within the study groups is visualized in Figure 4a,b. A principal coordinate analysis (PCoA) was constructed to investigate any microbiome compositional differences between the groups. Correlation analysis was performed using Spearman’s correlation, however no pattern or clustering were explained by the two groups (Figure 4c).

### 3.6. Comparable Ectocervical Epithelial Thickness and HIV Receptor Expression in HESN and NN Women

**Figure 5:**
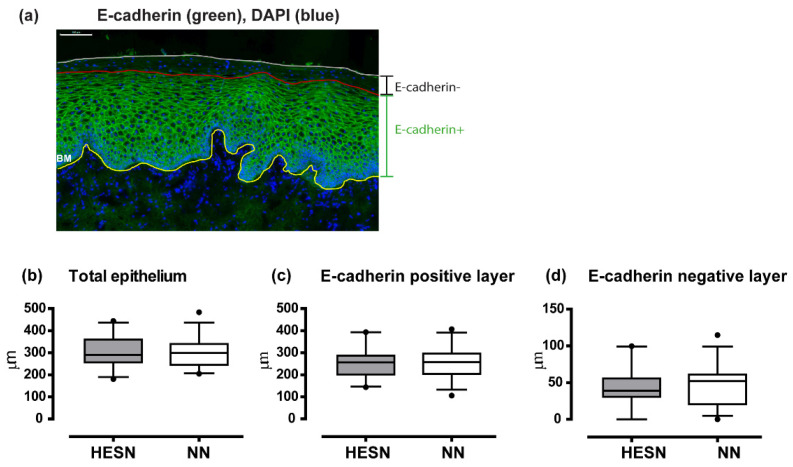
Ectocervical epithelial thickness of tissue samples from HESN and NN study participants. (**a**) Immunofluorescense image of a representative ectocervical tissue section stained for E-cadherin (green). DAPI (blue) was used as a counterstain for visualization of cell nuclei. The total height of the ectocervical epithelium was measured from the basal membrane (BM; indicated by the yellow line) to the last cell layer of the ectocervical epithelium (indicated by the white line). The height of the E-cadherin positive layer was measured from the basal membrane to the apical end of the E-cadherin-expressing layer (indicated by the red line). The E-cadherin negative layer was measured from the red to the white line. The images were collected with a 20× objective. Scale bar = 100 µm. The box plots show the median and the interquartile range, the whiskers show the 5–95% range, of (**b**), the total epithelial thickness, (**c**), the thickness of the E-cadherin-positive layer, (**d**), the thickness of the E-cadherin-negative layer. The Mann–Whitney *U* test was used to assess statistical significance between the two study groups and *p*-values < 0.05 were considered statistically significant.

Immunofluorescence staining for E-cadherin was used to visualize and quantify epithelial thickness. The epithelial thickness of the total, E-cadherin^+^ and E-cadherin^−^ layers was identified by manually drawn lines to indicate the basal membrane as well as the E-cadherin^+^ and E-cadherin^−^ layers of the ectocervical epithelium (Figure 5a). The average epithelial tissue areas analyzed at the first sample time point were similar between the study subjects (HESN: median 1.5 mm^2^, range 0.5–2.9; NN: median 1.6 mm^2^, range 0.5–2.7, *p* > 0.05). The thickness of the total (HESN: median 290 µm, IQ range 257–360; NN: median 299 µm, IQ range 246–340), as well as of the E-cadherin^+^ (HESN: median 257 µm, IQ range 202–287; NN: median 258, IQ range 203–296) and E-cadherin^−^ layers (HESN: median 39 µm, IQ range 31–56; NN: median 51 µm, IQ range 21–61), was also comparable (*p* > 0.05) (Figure 5b–d).

**Figure 6:**
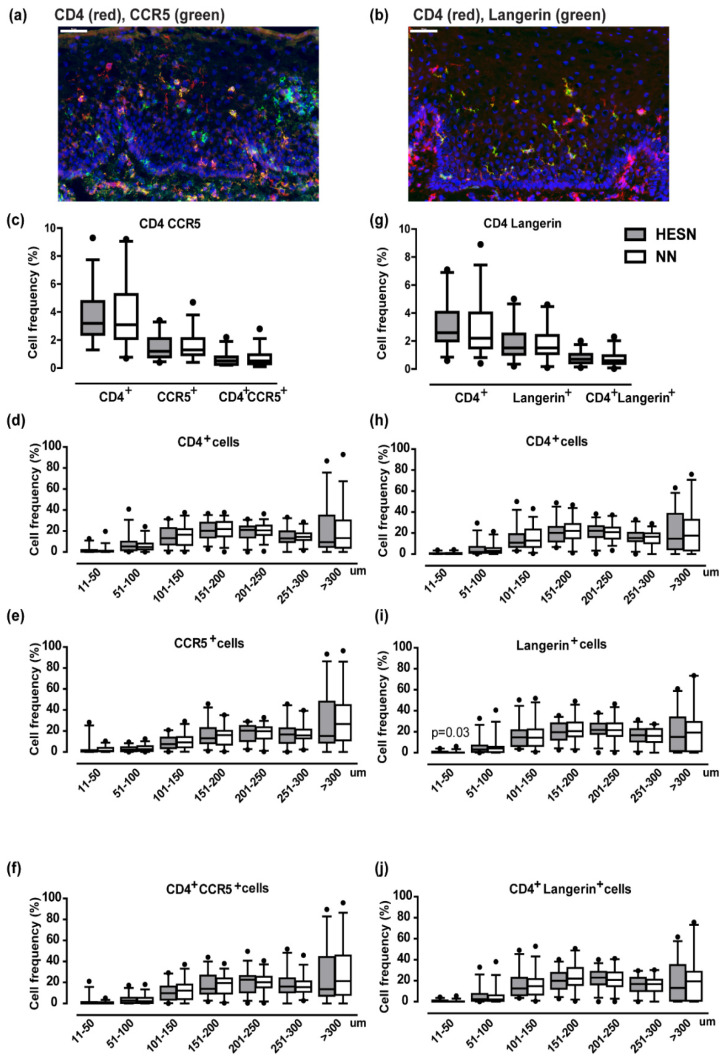
Visualization and enumeration of HIV target cell receptors in the ectocervical epithelium of HESN and NN study participants. Representative immunofluorescence images stained for: (**a**) CD4^+^ (red) together with CCR5^+^ (green), or (**b**) CD4^+^ (red) together with Langerin (green), in ectocervical tissue sections from one representative HESN woman. Double-positive cells are shown in yellow. DAPI (blue) was used as counterstain for visualization of cell nuclei. The images were collected with 40x objectives. Scale bars (=50 µm) are shown in white in the upper left corners of the images. The box plots in (**c**) and (**g**) show percentages of positively stained cell area out of total epithelial tissue area in HESN (n = 29) (grey boxes), and NN (n = 33) (white boxes). The box plots represent the median and interquartile range (whiskers 5–95%) for CD4^+^ (at time of CCR5 staining), CCR5^+^, and CD4^+^CCR5^+^ staining; CD4^+^ (at time of Langerin staining), Langerin^+^, and CD4^+^Langerin^+^ staining. Data from one HESN woman was not available for the Langerin staining experiment. CD4 was thus stained for twice, since the CCR5 and Langerin staining experiments were performed at different days. The box plots in (**d**–**f**) and (**h**–**j**) show percentages of positively stained cell area out of total epithelial tissue area in HESN (n = 29) (grey boxes) and NN (n = 33) (white boxes) and the x-axis represents the epithelial segments as counted from the apical border (in µm). The most apical segment (0–10 µm) is not included due to background autofluorescence. The Mann–Whitney *U* test was used to assess statistical significance between the two study groups and *p*-values < 0.05 were considered statistically significant.

Next, immunofluorescence staining was performed on the same sample set to compare the frequency and spatial distribution of CD4^+^, CCR5^+^, and Langerin^+^ cells (Figure 6a,b). The average epithelial tissue area analyzed for all markers was similar for the study subjects (CD4^+^CCR5^+^ staining: HESN: median 1.7 mm^2^, range 0.7–3.2; NN: median 2.1 mm^2^, range 1.1–3.5, *p* > 0.05; CD4^+^Langerin^+^ staining: HESN: median 1.7 mm^2^, range 0.5–2.9; NN: median 1.6 mm^2^, range 0.4–2.4, *p* > 0.05). The study groups displayed similar expression levels for CD4^+^ (HESN: median 3.2%, IQ range 2.4–4–8; NN: median 3.1%, IQ range 2.1–5.3), CCR5^+^ (HESN: median 1.2%, IQ range 0.6–2.1; NN: median 1.3%, IQ range 1.0–2.1), and CD4^+^CCR5^+^ (HESN: median 0.5%, IQ range 0.3–0.8; NN: median 0.5%, IQ range 0.3–1.0) (Figure 6c). To quantify the spatial distribution of the markers, the images were divided into 50 µm segments as counted from the apical border. The most apical 10 µm were excluded due to background autofluorescence. For samples with epithelium exceeding 300 µm thickness, the area >300 µm from the apical surface was represented in one single segment. There was no difference in spatial localization of CD4^+^, CCR5^+^, and CD4^+^CCR5^+^ cells between the study groups in any of the epithelial segments (Figure 6d–f).

Comparable expression (*p* > 0.05) was also recorded between the study groups for CD4^+^ (HESN: median 2.6%, IQ range 2.0–4.1; NN: median 2.2%, IQ range 1.5–4.0) stained together with Langerin^+^ (HESN: median 1.5%, IQ range 1.1–2.5; NN: median 1.5%, IQ range 1.1–2.4), and for CD4^+^Langerin^+^ (HESN: median 0.7%, IQ range 0.5–1.1; NN: median 0.6%, IQ range 0.4–1.0%) (Figure 6g). The distribution of CD4^+^, Langerin^+^ and CD4^+^Langerin^+^ cells, respectively, was found to be comparable (*p* > 0.05) for all segments, except for Langerin^+^ cells in the most apical layer (HESN: median 0.3%; NN: median 0.1%, *p* = 0.03) (Figure 6h–j).

The image analyses were confirmed in the set of tissue samples collected at the second sample time point (approximately two weeks later). A total of 28 samples from the HESN group, and 31 samples (overlapping with 26 samples from above) from the NN group were available. None of the sociodemographic or other confounding parameters differed significantly from those presented from the first sample time point (*p* > 0.05) (data not shown). Plasma progesterone levels was not measured at this visit. There were no statistical significant differences between the study subjects for neither the total epithelial thickness (HESN: median 317 µm, IQ range 279–425; NN: median 320 µm, IQ range 262–381), nor for the thickness of the E-cadherin^+^ (HESN: median 247 µm, IQ range 217–334; NN: median 237 µm, IQ range 202–318), or E-cadherin^−^ layers (HESN: median 75 µm, IQ range 46–91; NN: median 66 µm, IQ range 51–96). Neither did the total frequency of CD4^+^ cells differ between the HESN and NN groups (HESN: median 1.4%, IQ range 0.7–2.8; NN: median 1.4%, IQ range 0.8–3.0) (Figure S4a–d).

### 3.7. Comparable Transcriptional Activity in Ectocervical Tissue of HESN and NN Women

**Figure 7:**
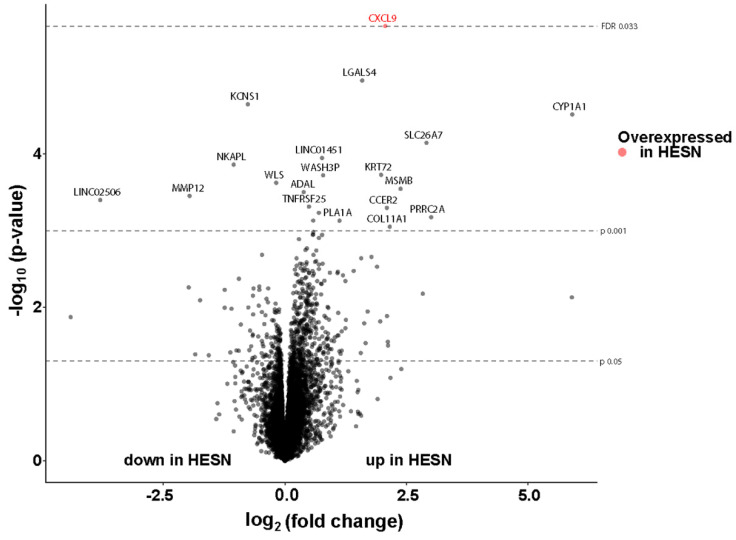
Volcano plot of the differentially expressed genes between the HESN and NN study participants. Each dot represents one of the 15,445 genes identified by RNA sequencing of ectocervical tissue samples. The x-axis represents log_2_ fold change of gene expression. Negative values represent under-expressed genes and positive values represent over-expressed genes in the HESN group as compared to the NN group. The Y-axis represents the −log_10_ *p*-values. One statistically significant differentially expressed gene (CXCL9), as defined by a FDR < 0.05, is marked in red.

RNA sequencing was performed on RNAlater-preserved tissue samples from the second sample time point. Demographic parameters were similar in the two groups despite two additional samples from the HESN group and one from the NN group at this second sample time point as described above (Section 3.6). All ectocervical biopsies displayed good quality of isolated RNA and generated >10 million reads each. The transcriptional activity was comparable (FDR > 0.05) between the HESN and NN study groups. Of the total 15,445 genes identified by RNA-sequencing, only CXCL9 (log_2_ fold change 2.1, FDR adj. *p* = 0.03) was upregulated in the HESN group (Figure 7).

## 4. Discussion

We here investigated the genital mucosal microenvironment of female sex workers in a unique collection of samples to identify factors defining HESN individuals. Previous correlates to the HESN phenotype in this cohort [2,3] were confirmed and extended by novel phenotypic markers, including increased proportions of Tregs in blood and lower proportions of CMC-derived activated T cells. By assessing genital secretions, neither protein levels nor the distribution of the microbiome community types differed significantly between the study groups. In addition to these results, the main aim of the study was to investigate the corresponding unique ectocervical tissue samples for novel mucosal correlates to the HESN phenotype. These factors included ectocervical epithelial tissue structure, expression of E-cadherin, spatial distribution of HIV target cell receptors and the mRNA transcriptome. However, all these factors were found to be comparable (*p* > 0.05) in the HESN group compared to the control group. Thus, the HESN phenotype of female sex workers is predominantly defined by an intact ectocervical mucosa.

Systemic aberrant immune activation is a strong correlate of HIV disease progression, and immune activation in the genital mucosa is a strong risk factor for acquiring sexually transmitted HIV [21,43,44,45]. Tregs can suppress cellular immune activation and may have a role in maintaining a non-inflammatory status in the genital mucosa of HESN individuals [46]. Previously, it was observed that blood-derived classical Tregs (CD4^+^CD25^+^FoxP3^+^) were elevated in HESN relative to high-risk HIV-negative controls and to HIV-infected individuals [2]. Here, these results were confirmed and extended by using a broader panel of CD4^+^ Treg markers including B7, a part of the integrin α4β7 molecule, and the transcription factors FoxP3 and Helios, in combination with CD39 and CD127. We thus observed a higher proportion of Tregs expressing B7 in HESN versus control individuals. The expression of B7 is associated with a subset of Tregs that has a better capacity to induce suppressive T cells and produce high levels of IL-10, as well as being specialized to migrate to mucosal tissue to prevent inflammation [42]. This higher proportion of Tregs expressing B7 was also observed when looking at Tregs expressing FoxP3 and Helios. Helios is a key transcription factor that stabilizes Tregs in the face of inflammatory responses [47]. Here we observed that the Helios^+^ Tregs in the HESN individuals had a higher level of CD39 on a per cell basis. Human CD39^hi^ Tregs present stronger stability and function under inflammatory conditions [48]. Although the overall low numbers of CMCs that were available did not allow the analysis on Treg activity, it was shown that the CMCs from the HESN group had significantly lower T-cell activation (CD4^+^CCR5^+^CD69^+^ and CD8^+^CCR5^+^CD69^+^ cells) than the control group. T cells upregulate CCR5 and CD69 upon activation and CD69 is also a marker for tissue-retention and is expressed on tissue-residing cells [49,50]. Together the results of our study showed that the HESN group, compared to the control group, had a higher frequency of systemic Tregs that possessed markers of suppressive activity to control inflammation. Although a causal link cannot be proven, it can be speculated that the Treg activity in blood, expressing the mucosal homing marker B7, could contribute to the lower levels of activated CMCs in the HESN group.

By assessing protein levels representing a broad panel of markers for inflammation and genital epithelial barrier structure, we did not confirm previous data on an increased cervicovaginal anti-inflammatory protein profile reported in HESN individuals [8,9]. A significant difference in sampling between this study and others was that our protein profiling focused on luteal phase sampling rather than on unselected stages of the menstrual cycle. In addition, a panel of 14 pre-defined cytokines was assessed, revealing comparable protein levels (*p* > 0.05) except for a significantly lower level of the pro-inflammatory marker IL-8 in the HESN group as compared with the control group. Several studies indicate that the endogenous microflora influence the genital proteome [24,51]. Here, the study groups were comparable for prevalence of both clinical and Nugent score-defined BV, as well as for a pre-defined classification [33] of cervicovaginal microbial community types. This classification defined *Lactobacillus*-dominant non-*iners* and *iners* types as well as *Gardnerella*-dominant and polymicrobial community types. Previous observations on HESN female commercial sex workers have also shown comparable cervicovaginal microbiota compared to controls [52]. Thus, neither the cervicovaginal protein levels nor the microbiome composition correlated with the HESN phenotype in the present study population.

Following assessments of PBMC, CMC and cervicovaginal secretion samples, we next studied the tissue microenvironment in detail. The epithelial structure was visually inspected on all prepared tissue slides followed by objective measurements of the height of the epithelium as a structural marker of the mucosal barrier. We could thereby demonstrate that both the E-cadherin positive and negative layers, respectively, were found comparable (*p* > 0.05) between HESN and control women. A thick epithelium with few and distantly located HIV target cells could in theory hinder or limit HIV infectious particles to reach the submucosa. Exogenous factors such as hormonal contraceptive use have been correlated to a thinner apical ectocervical epithelial layer, proving the feasibility of such measurements for evaluating factors of importance for HIV susceptibility [37]. Next, the expression and spatial localization of the potential HIV target cell populations CD4^+^CCR5^+^ and CD4^+^Langerin^+^ cells were examined and found comparable (*p* > 0.05) between the groups. Further phenotyping of the CD4^+^CCR5^+^ cells in our study are needed to determine whether they primarily represent CD3^+^ T cells or cells of myeloid origin. CD4^+^Langerin^+^ cells may contribute to both binding and degrading of HIV, and to the opposite function, binding and further spread of infectious HIV to other cell populations [53]. The ectocervical tissue samples, including both the epithelium and submucosa, from the HESN and control study subjects were further characterized by their transcriptional profile at the mRNA level. An intact tissue microenvironment of the ectocervical mucosa in HESN individuals was thereby further defined by the lack of any statistically significant differential gene expression between the study groups, except for the chemokine CXCL9. It is however not relevant to speculate about the functional implication of a single differentially expressed gene, among the more than 15,000 identified genes, at this level of significance.

The strengths of this study were that it was performed ex vivo using genital mucosal samples from a well-defined group of HESN participants. To control for the influence of sex hormones, only women who were not using hormonal contraceptives were included since sex hormone levels and use of hormonal contraceptives can significantly affect the apical (E-cadherin^−^) epithelial layer, as well as the genital proteome and transcriptome [22,23,25,54,55,56,57]. As the study was controlled for menstrual cycle stage, our study groups had comparable progesterone levels. Limitations of the study included not controlling for HSV-2 serostatus and other sociodemographic factors that may have impacted the results [58,59,60,61]. Due to the low numbers of cells available in CMC samples, the study was not able to assess Treg levels in mucosal samples. Furthermore, as these sex workers were sampled more recently, they may be less exposed to HIV than individuals assessed in previous reports from the same cohort [16]. The reported condom use in the cohort has increased during the last decades (own unpublished data), the clients who are HIV-infected are more likely to be on anti-retroviral medication and the HIV-seroprevalence is lower in Kenya [1]. To partly compensate for these factors, the present HESN population was defined as being active in sex work for 7 years as compared to the previous definition of 3 years [16,62]. In both of these studies, HIV acquisition was significantly associated with a shorter base line duration of sex work. As sex work itself may induce changes to the vaginal immune milieu, we here selected newly recruited sex workers as controls as they would have similar sexual practices. Previous HESN studies have used HIV-seronegative women who were not sex workers as controls which may also have yielded more pronounced differences in mucosal biomarkers between cases and controls.

Correlates of protection against HIV infection in HESN individuals may include other immune cell populations and soluble factors that were not studied here. Nevertheless, the novel approach to characterize mucosal immune correlates of relative HIV resistance in tissue compartments of the female genital tract is an important step toward understanding HIV susceptibility factors and mucosal immunology and will hopefully contribute to an extended evaluation of new prevention strategies.

## 5. Conclusions

The identification of an intact ectocervical microenvironment in HESN individuals add new data to current knowledge about natural resistance to sexual transmission of HIV.

## Figures and Tables

**Table 1:** Clinical characterization of study subjects at enrolment.

Parameter	HESN (n = 29)	NN (n = 33)	*p*-Value
	Median or Number (Range or %)	Median or Number (Range or %)	
Age (years)	37 (30–50)	31 (21–47)	0.001
Years since last pregnancy ^a^	7 (1–19)	8 (5–22)	ns
Progesterone levels (ng/mL) ^b^	2.6 (0.1–17)	3.9 (0.1–16.4)	ns
BV ^c^			
Negative (0–3)	14 (48%)	17 (53%)	ns
Intermediate (4–6)	9 (31%)	6 (19%)	ns
Positive (7–10)	6 (21%)	9 (28%)	ns
Yeast infection	1 (3%)	1 (3%)	ns
No. of clients last 7 days	5 (0–50)	4.5 (0–30)	ns
Unprotected sex acts last 7 days ^d^	0 (0–6)	0 (0–1)	ns
Vaginal douching ^e^	13 (45%)	8 (24%)	ns

ns = non-significant (*p* < 0.05), Mann–Whitney *U* test and Fisher’s exact test; ^a^ Years since last pregnancy, including abortions; Data not available from 4 HESN and 1 NN participants, respectively; ^b^ Progesterone levels at the first sample time point; ^c^ BV: Bacterial vaginosis (Nugent’s score, 1–10; negative BV = 0–3; Intermediate BV = 4–6; Positive BV = 7–10); ^d^ No. of unprotected sex acts with clients last 7 days. Calculated based on self-reported use of condom with clients; ^e^ Any douching performed by inserting water, or water and soap, in the vagina since last study visit (approximately 2 weeks).

## Data Availability

Processed data files and transcriptomics meta data are available at Gene Expression Omnibus (GEO) under accession number GSE165132.

## Supplementary Materials

The following are available online at https://www.mdpi.com/2076-393X/9/3/217/s1, Table S1: Gene description. Table S2: Clinical characterization of study subjects (CMC analyses). Figure S1: Gating strategies. Figure S2: Protein measurement in CVL. Figure S3: Cytokine measurement in CVL. Figure S4: Ectocervical epithelial thickness and frequency of CD4 cells (the second sample time-point).
