# Supplementary material for: HIV-Exposed Seronegative Sex Workers Express Low T-Cell Activation and an Intact Ectocervical Tissue Microenvironment
Source: Vaccines (Basel). 2021 Mar 4;9(3):217. doi: 10.3390/vaccines9030217 (PMC7998094; doi:10.3390/vaccines9030217)
Supplement: Supplementary file 1 [file vaccines-09-00217-s001.zip › vaccines-1102333-new-supplementary/Fig legends S1-S4.pdf]

Figure S1. *Phenotyping of PBMC and CMC by flow cytometry*
Gating strategies of cells were done as follows: singlets; lymphocyte gating; CD3+ live; CD4+/CD8+; different markers out of the CD4+ or CD8+ parent populations.

Figure S2. *Protein measurement in CVL*
Expression of proteins in CVL in the two study groups (HESN and NN). The log-transformed data is presented as fluorescence intensity (arbitrary units) per sample and bead identity. The differences in protein levels between the two sample groups were evaluated using the Mann-Whitney *U* test, and p-values <0.05 were regarded significant. A total of 74 proteins, represented by 90 antibodies (Table S1), were sorted from the lowest to the highest p-value (all p-values>0.05).

Figure S3. *Cytokine measurement in CVL*
Expression of cytokines in the two study groups (HESN and NN) with data presented as median and interquartile range. The values are given as pg/mL. None of the cytokines differed significantly between the groups (p>0.05). CVL: cervicovaginal lavage.

Figure S4. *Ectocervical epithelial thickness and frequency of CD4^{+} cells*
Analysis of the second sample time point; HESN (n=29) vs. NN (n=31). The boxplots show the median and interquartile range (whiskers 5-95%) of (a), the total epithelial thickness, (b) the thickness of the E-cadherin positive layer, (c) the thickness of E-cadherin negative layer and (d**)** percentage of positively stained cell area for CD4^{+} (at time of CCR5 staining) out of total epithelial tissue area. The Mann-Whitney *U* test was used to assess statistical significance between the two study groups and p-values <0.05 were considered statistically significant.
