# Supplementary material for: HIV-Exposed Seronegative Sex Workers Express Low T-Cell Activation and an Intact Ectocervical Tissue Microenvironment
Source: Vaccines (Basel). 2021 Mar 4;9(3):217. doi: 10.3390/vaccines9030217 (PMC7998094; doi:10.3390/vaccines9030217)
Supplement: Supplementary file 1 [file vaccines-09-00217-s001.zip › vaccines-1102333-new-supplementary/Fig S2 proteins.pdf]

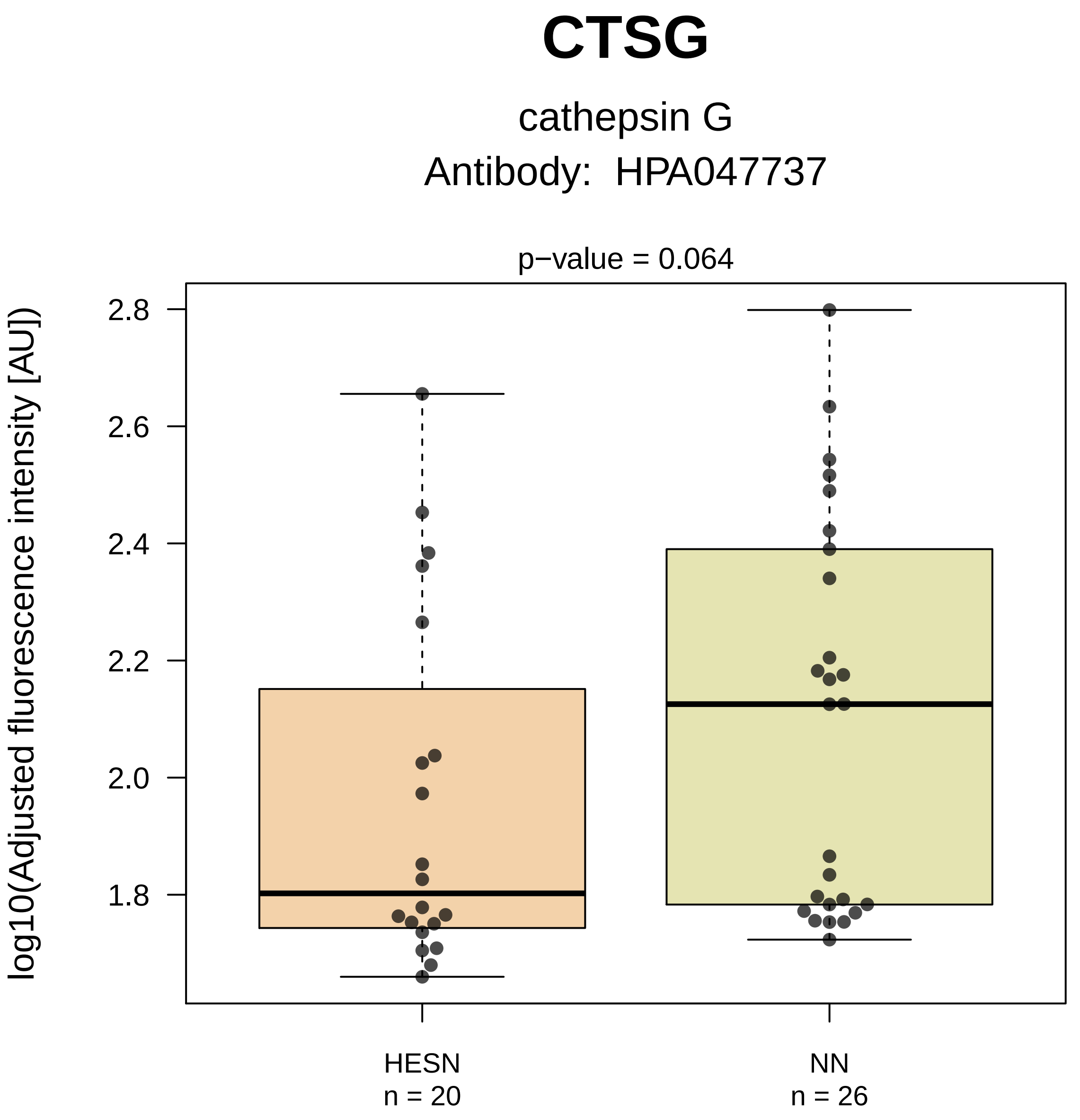
CTSG
cathepsin G
Antibody: HPA047737
p−value = 0.064
log10(Adjusted fluorescence intensity [AU])
2.8
2.6
2.4
2.2
2.0
1.8
HESN
n = 20
NN
n = 26

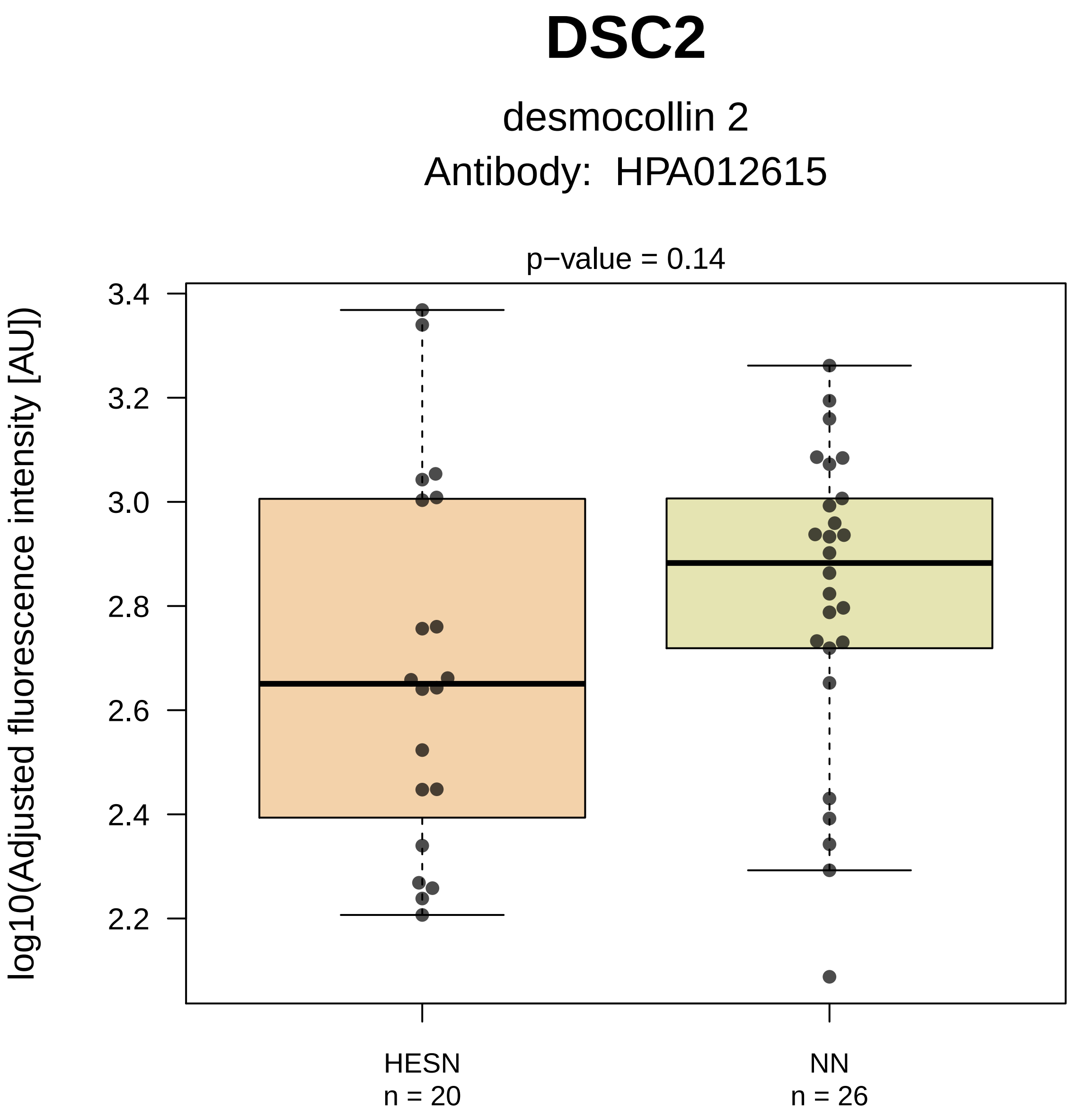
DSC2
desmocollin 2
Antibody: HPA012615
p−value = 0.14
log10(Adjusted fluorescence intensity [AU])
3.4
3.2
3.0
2.8
2.6
2.4
2.2
HESN
n = 20
NN
n = 26

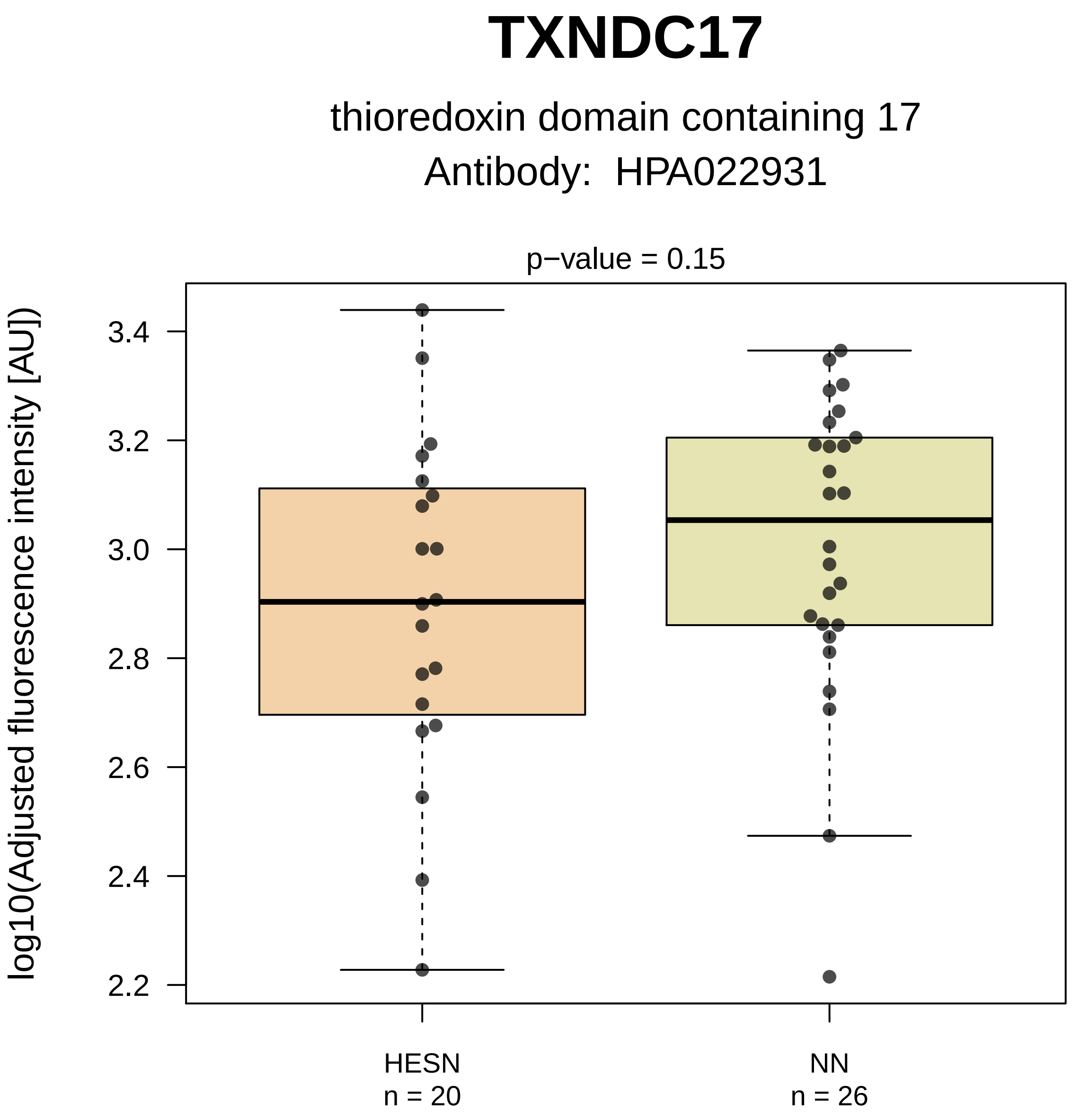
TXNDC17
thioredoxin domain containing 17
Antibody: HPA022931
p−value = 0.15
log10(Adjusted fluorescence intensity [AU])
3.4
3.2
3.0
2.8
2.6
2.4
2.2
HESN
n = 20
NN
n = 26

FGA

fibrinogen alpha chain
Antibody: HPA064755

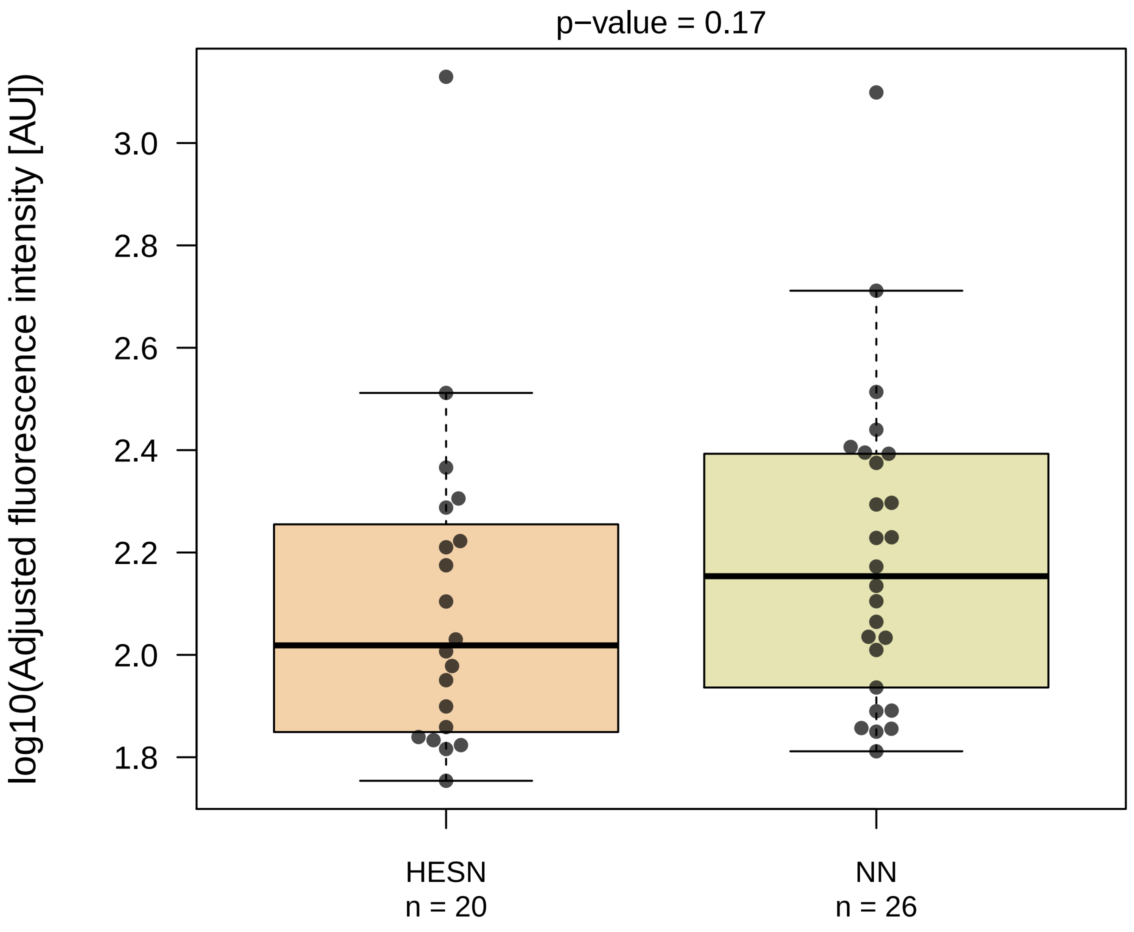

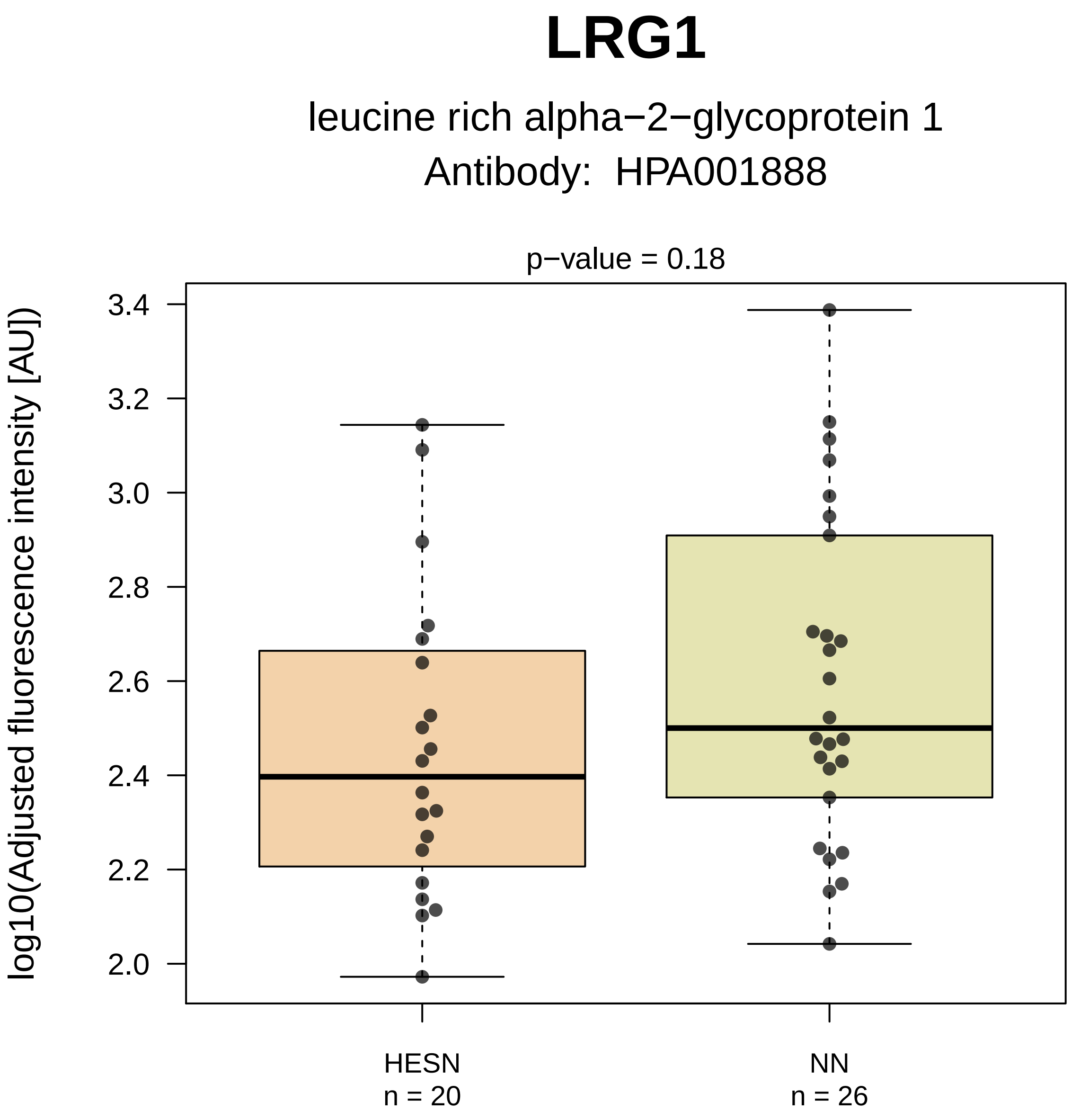
LRG1
leucine rich alpha−2−glycoprotein 1
Antibody: HPA001888
p−value = 0.18
log10(Adjusted fluorescence intensity [AU])
3.4
3.2
3.0
2.8
2.6
2.4
2.2
2.0
HESN
n = 20
NN
n = 26

FGA

fibrinogen alpha chain
Antibody: HPA051370

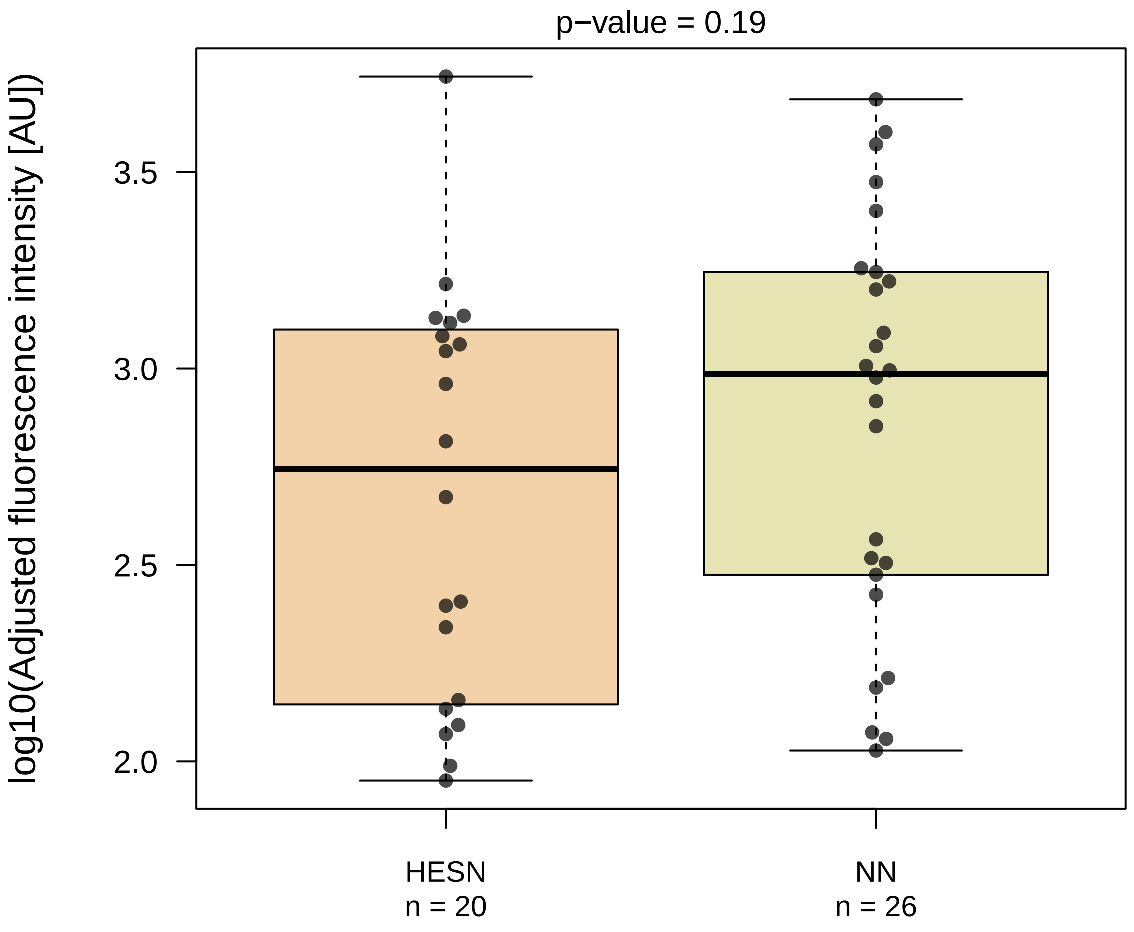

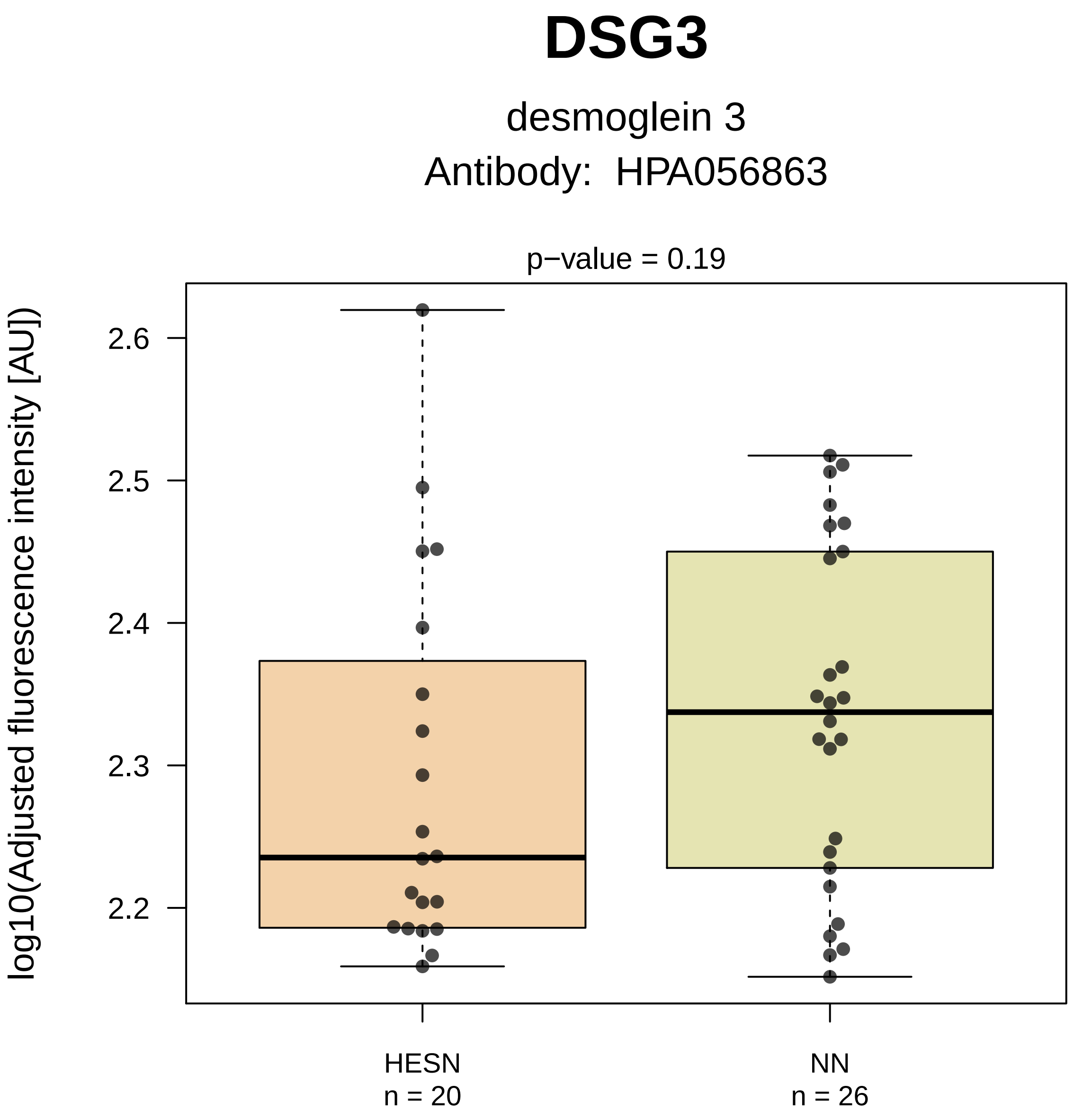
DSG3
desmoglein 3
Antibody: HPA056863
p−value = 0.19
log10(Adjusted fluorescence intensity [AU])
2.2
2.3
2.4
2.5
2.6
HESN
n = 20
NN
n = 26

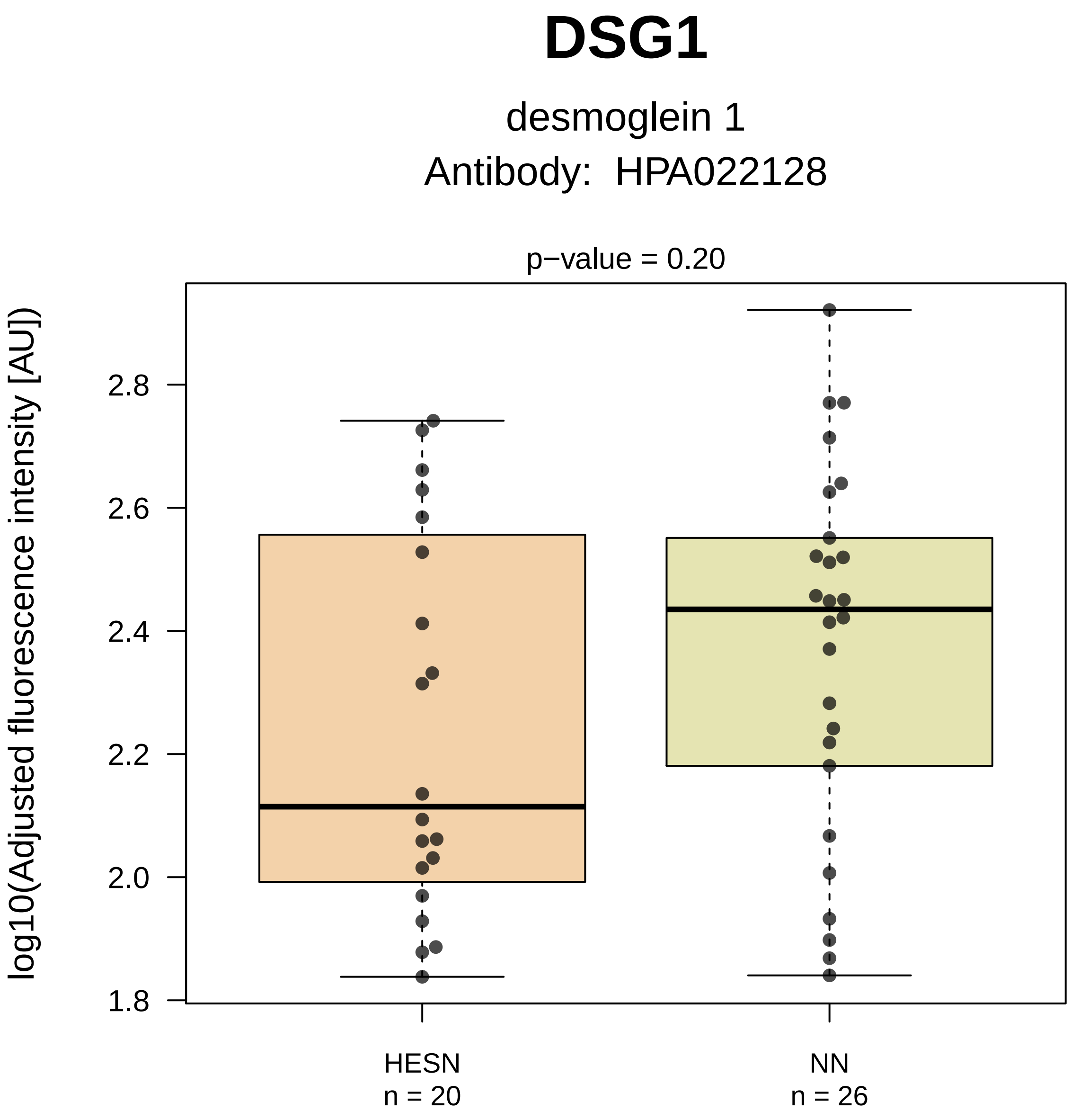
DSG1
desmoglein 1
Antibody: HPA022128
p−value = 0.20
log10(Adjusted fluorescence intensity [AU])
2.8
2.6
2.4
2.2
2.0
1.8
HESN
n = 20
NN
n = 26

CD5L

CD5 molecule like

Antibody: HPA026432

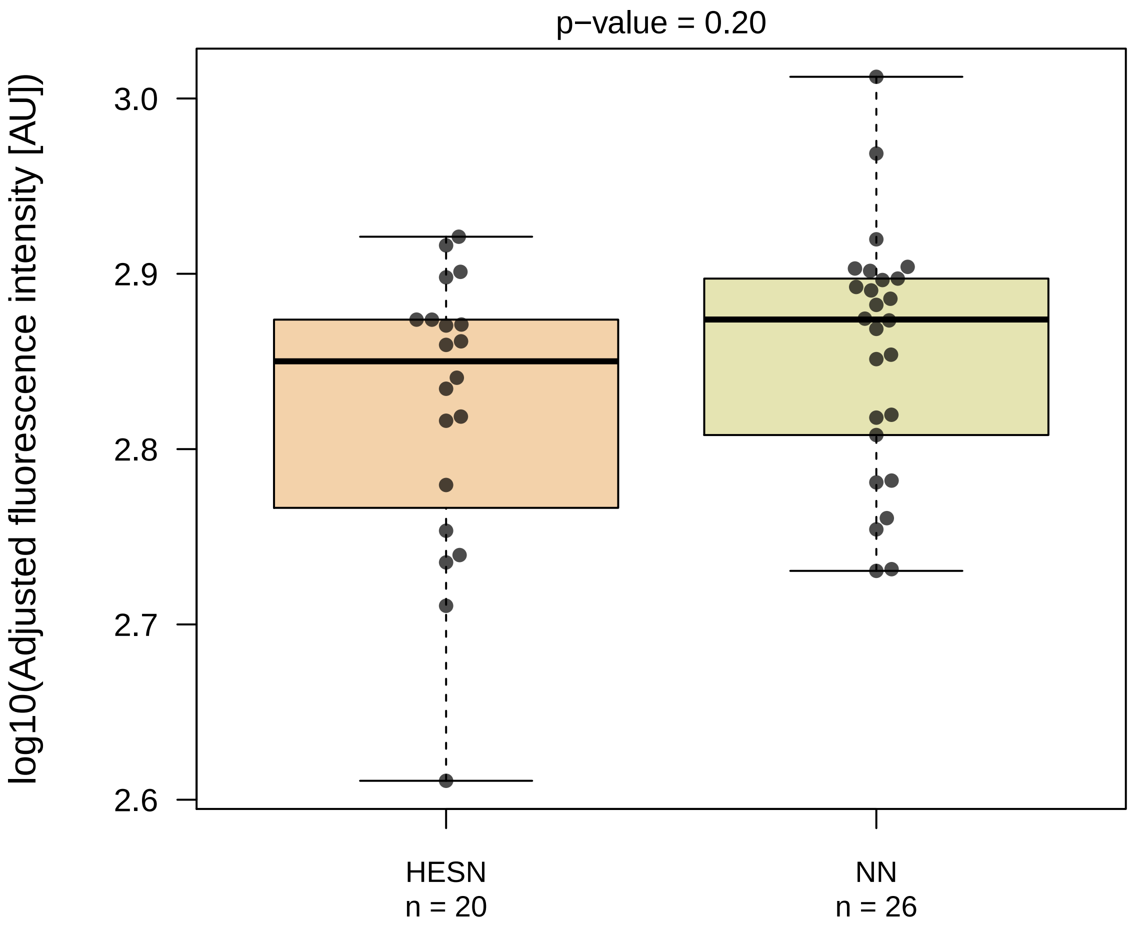

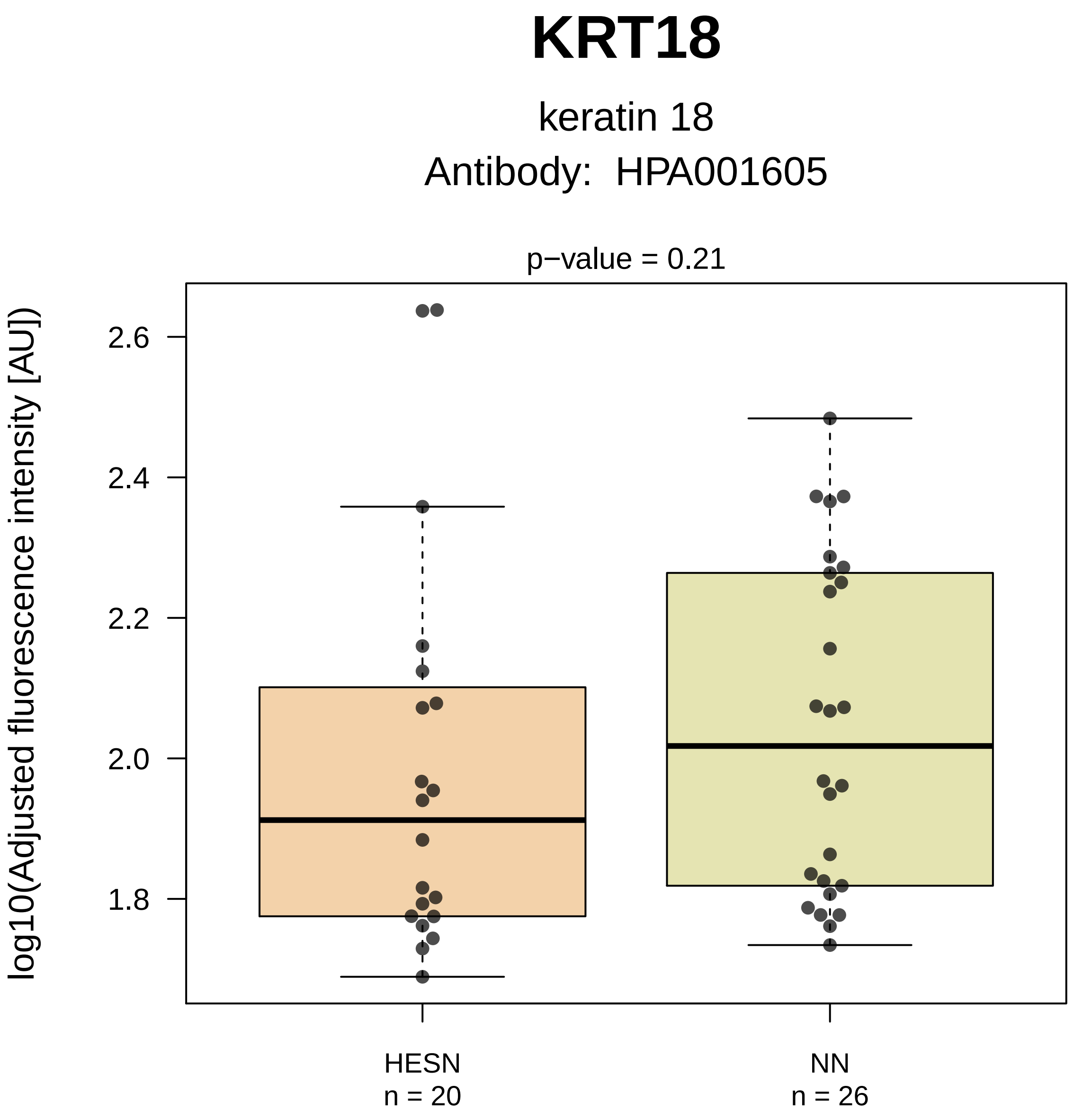
KRT18
keratin 18
Antibody: HPA001605
p−value = 0.21
log10(Adjusted fluorescence intensity [AU])
2.6
2.4
2.2
2.0
1.8
HESN
n = 20
NN
n = 26

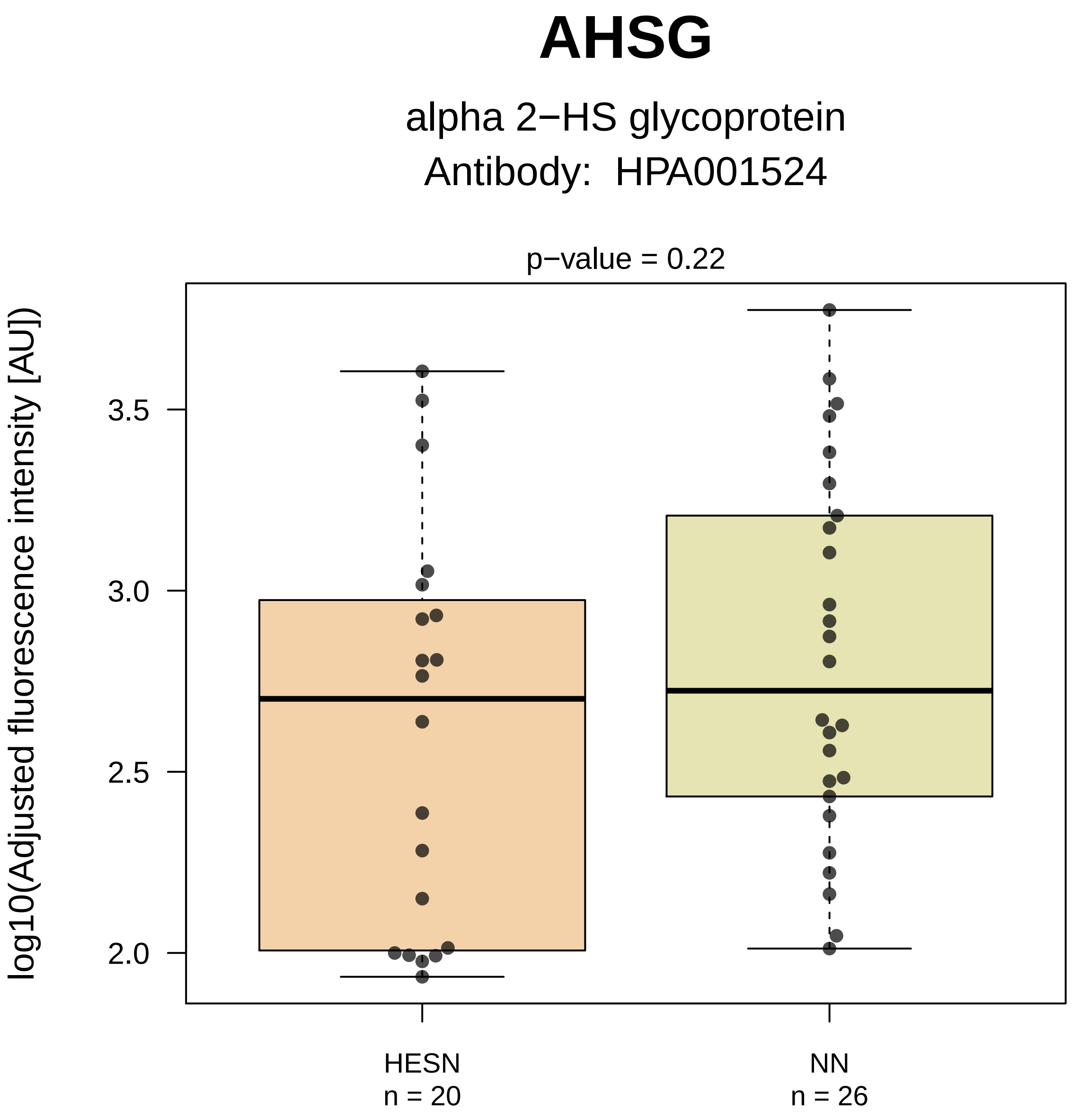
AHSG
alpha 2−HS glycoprotein
Antibody: HPA001524
p−value = 0.22
log10(Adjusted fluorescence intensity [AU])
3.5
3.0
2.5
2.0
HESN
n = 20
NN
n = 26

SPRR3

small proline rich protein 3
Antibody: HPA024330

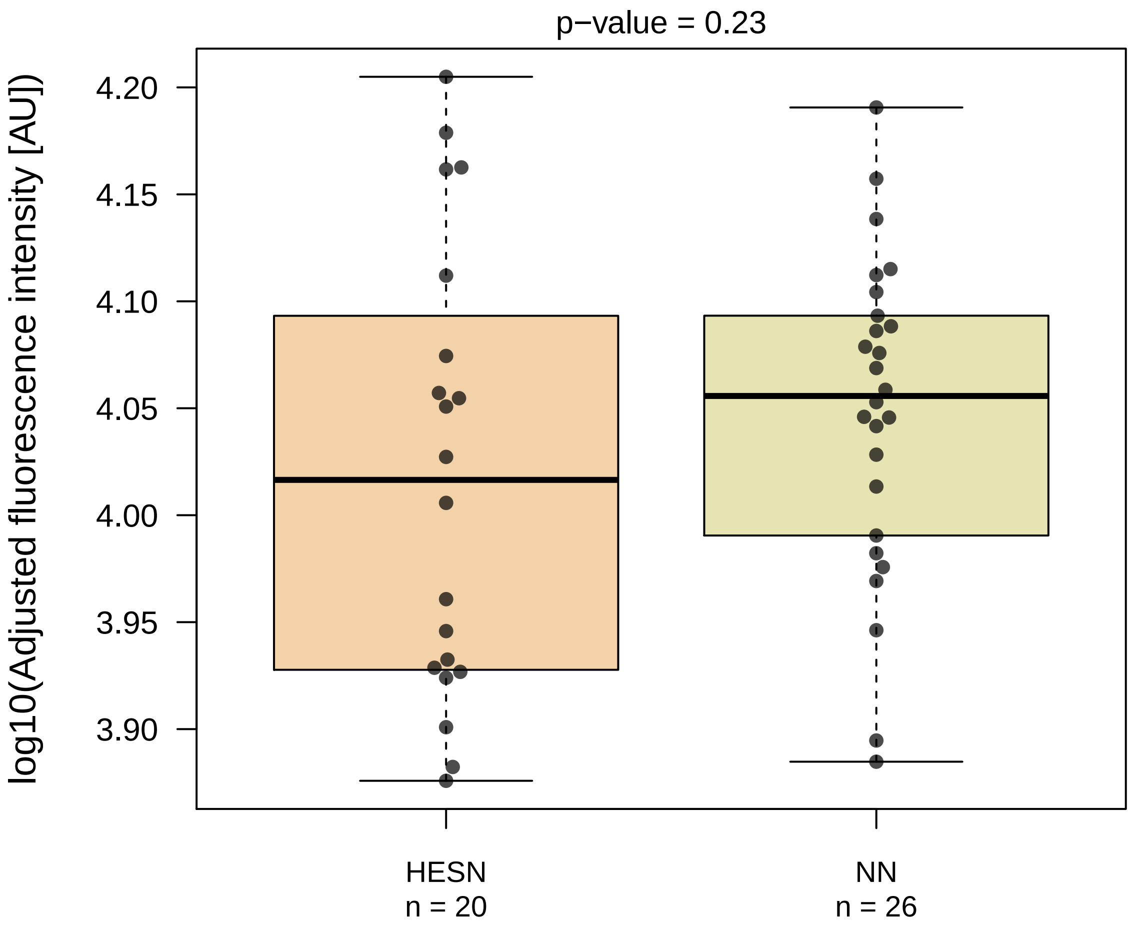

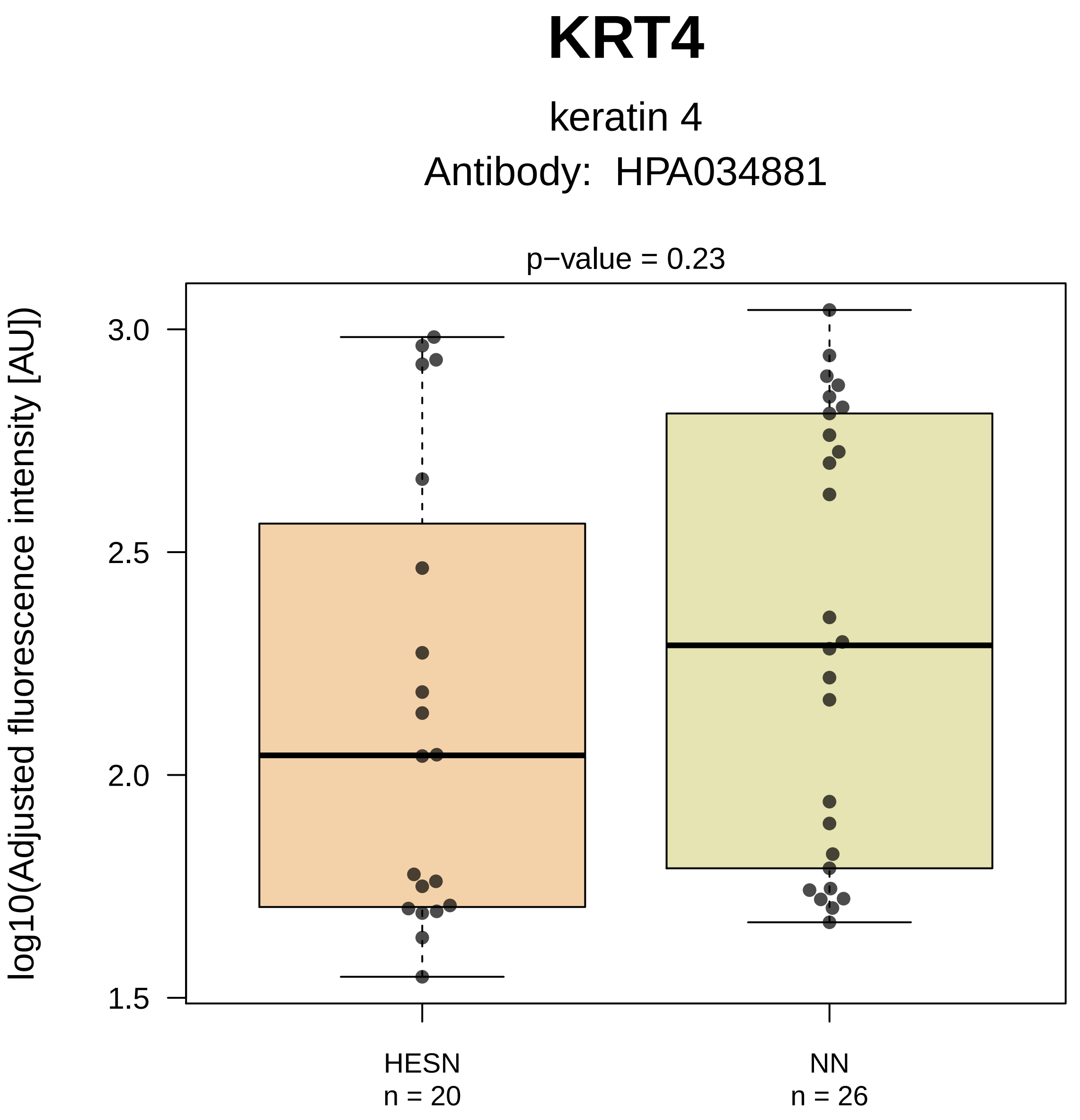
KRT4
keratin 4
Antibody: HPA034881
p−value = 0.23
log10(Adjusted fluorescence intensity [AU])
3.0
2.5
2.0
1.5
HESN
n = 20
NN
n = 26

S100A2

S100 calcium binding protein A2

Antibody: HPA034651

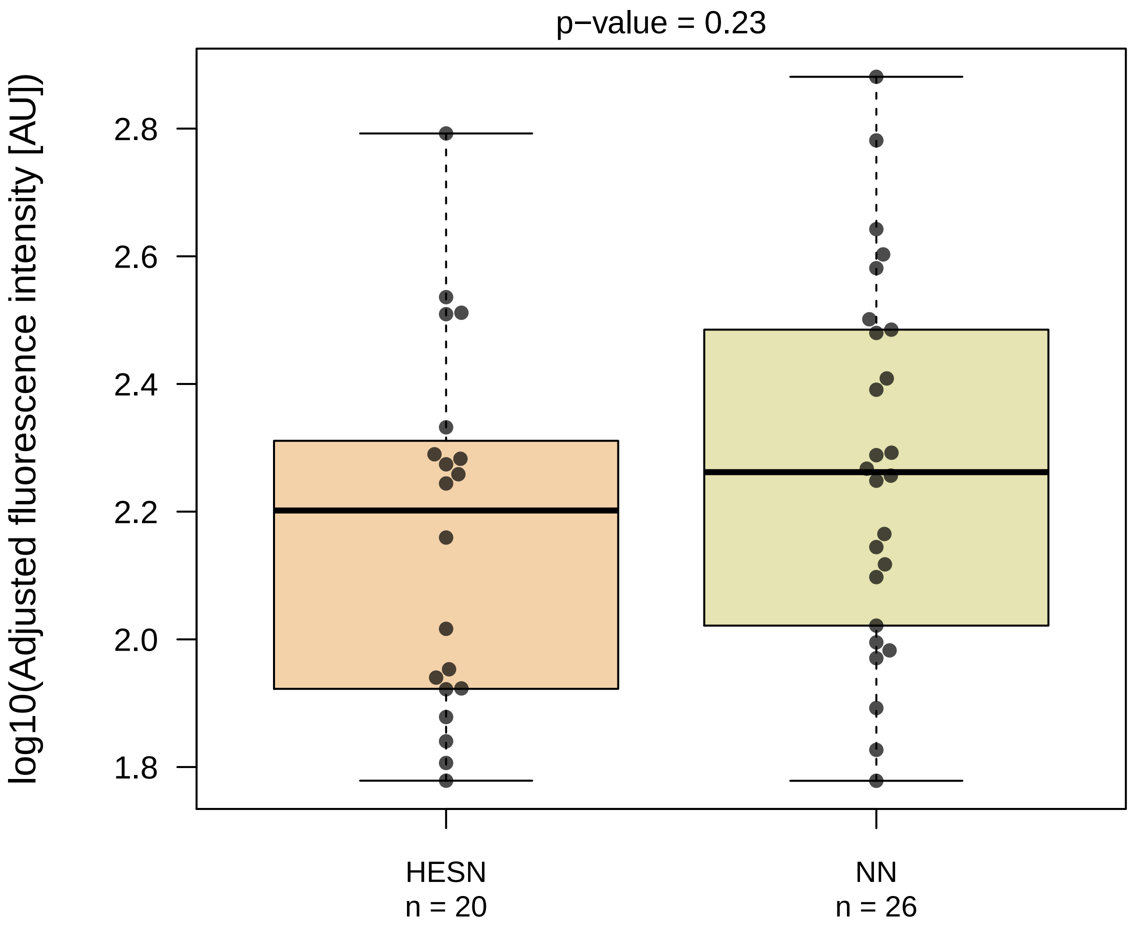

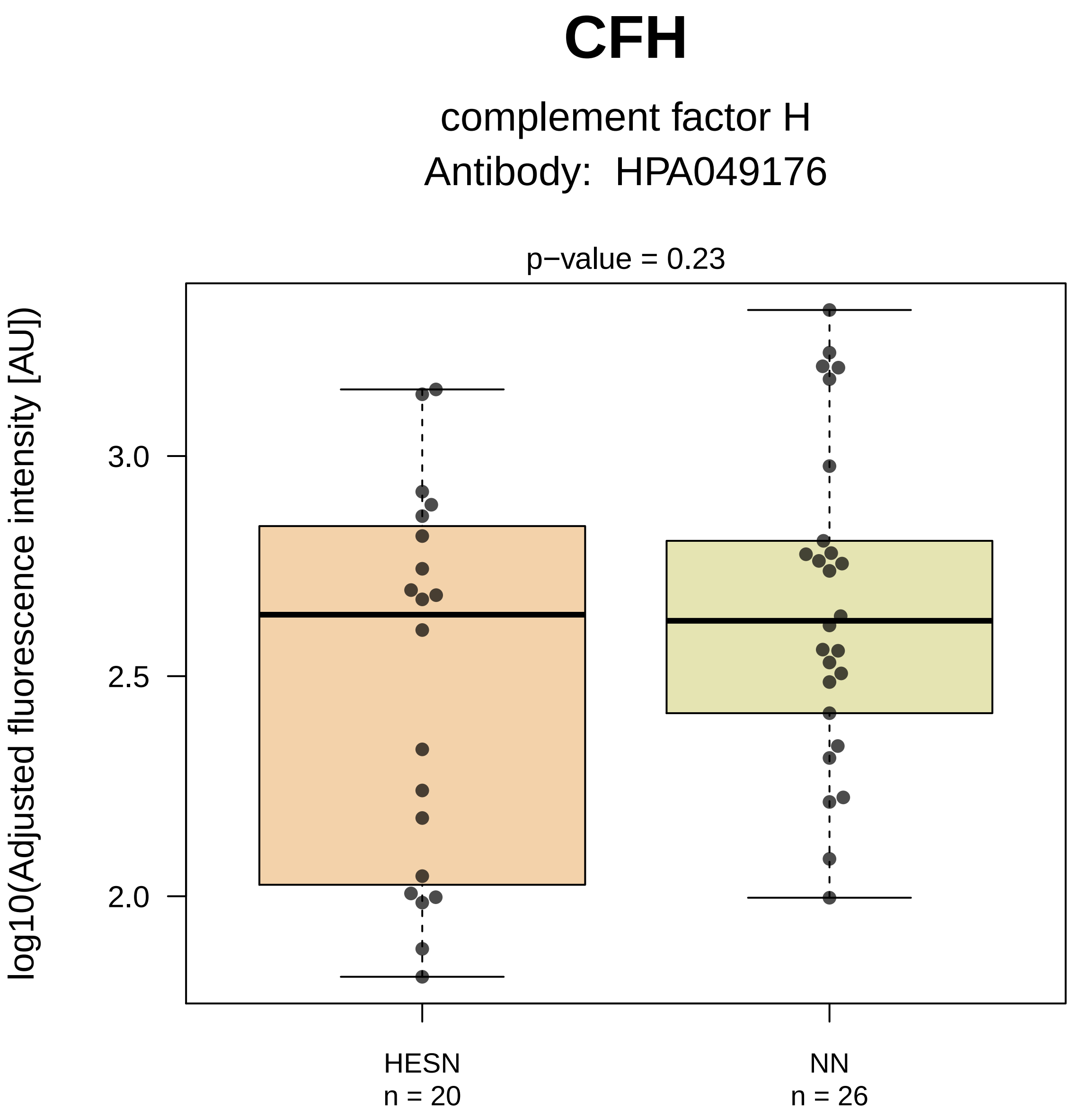
CFH
complement factor H
Antibody: HPA049176
p−value = 0.23
log10(Adjusted fluorescence intensity [AU])
3.0
2.5
2.0
HESN
n = 20
NN
n = 26

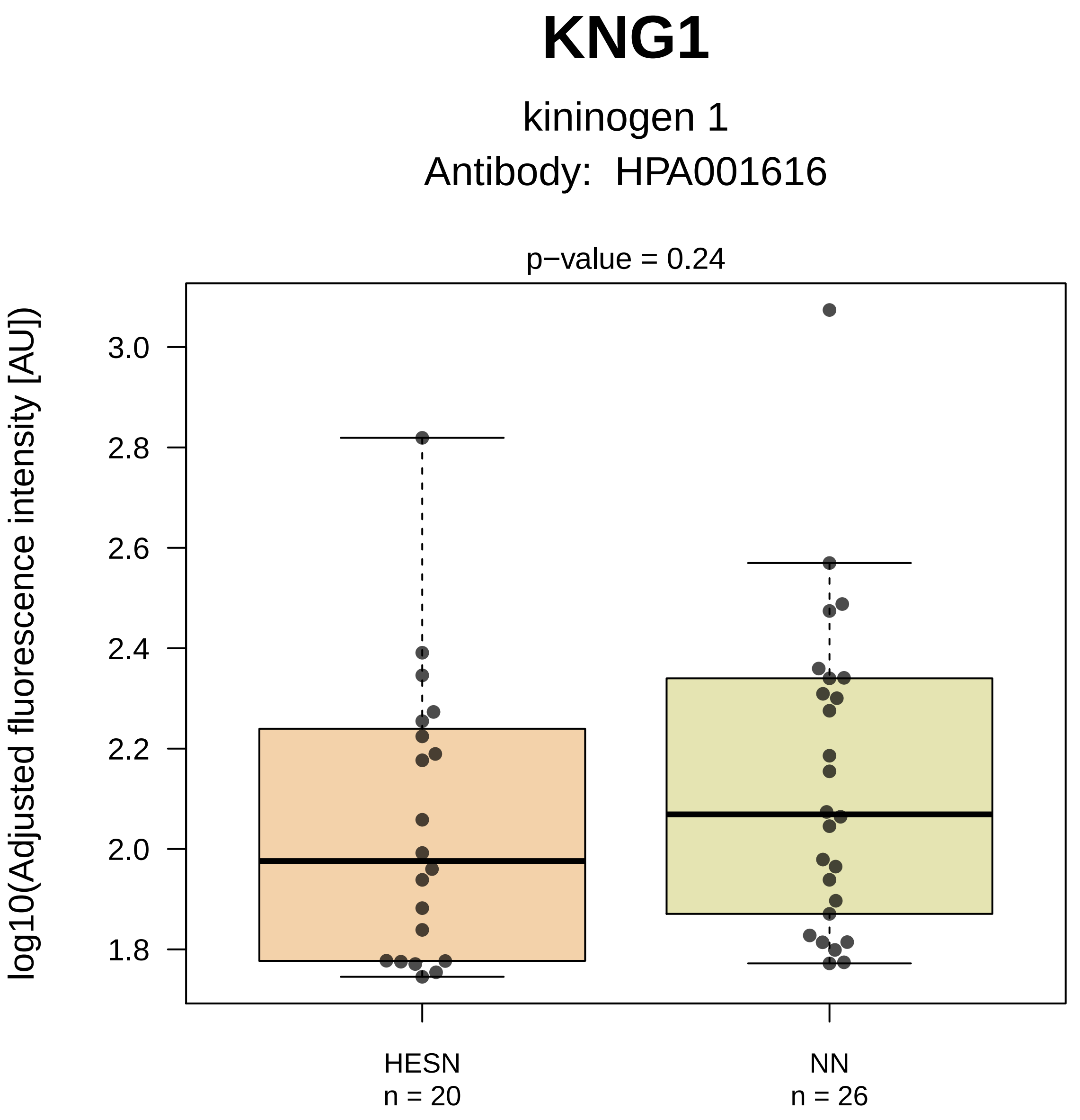

KNG1
kininogen 1
Antibody: HPA001616
p−value = 0.24
log10(Adjusted fluorescence intensity [AU])
3.0
2.8
2.6
2.4
2.2
2.0
1.8
HESN
n = 20
NN
n = 26

FLNA

filamin A

Antibody: HPA002925

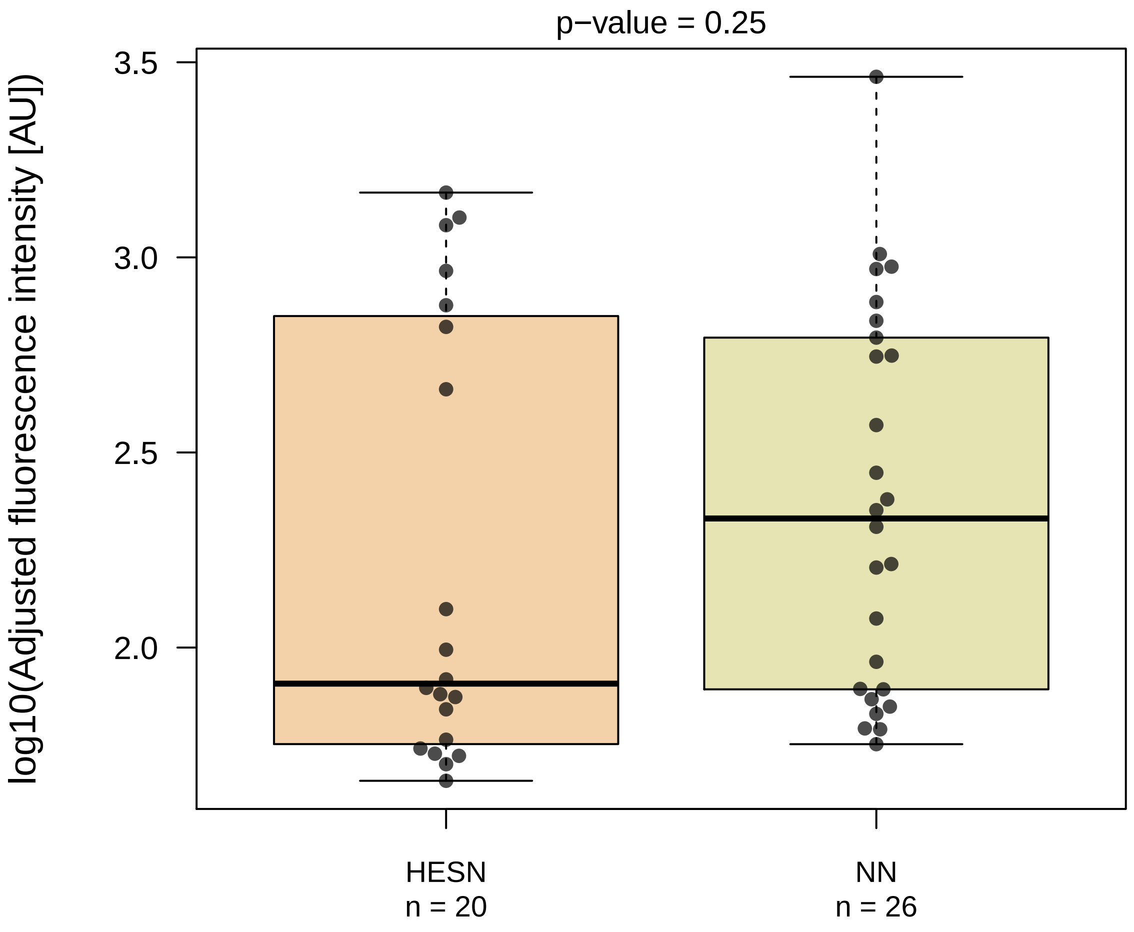

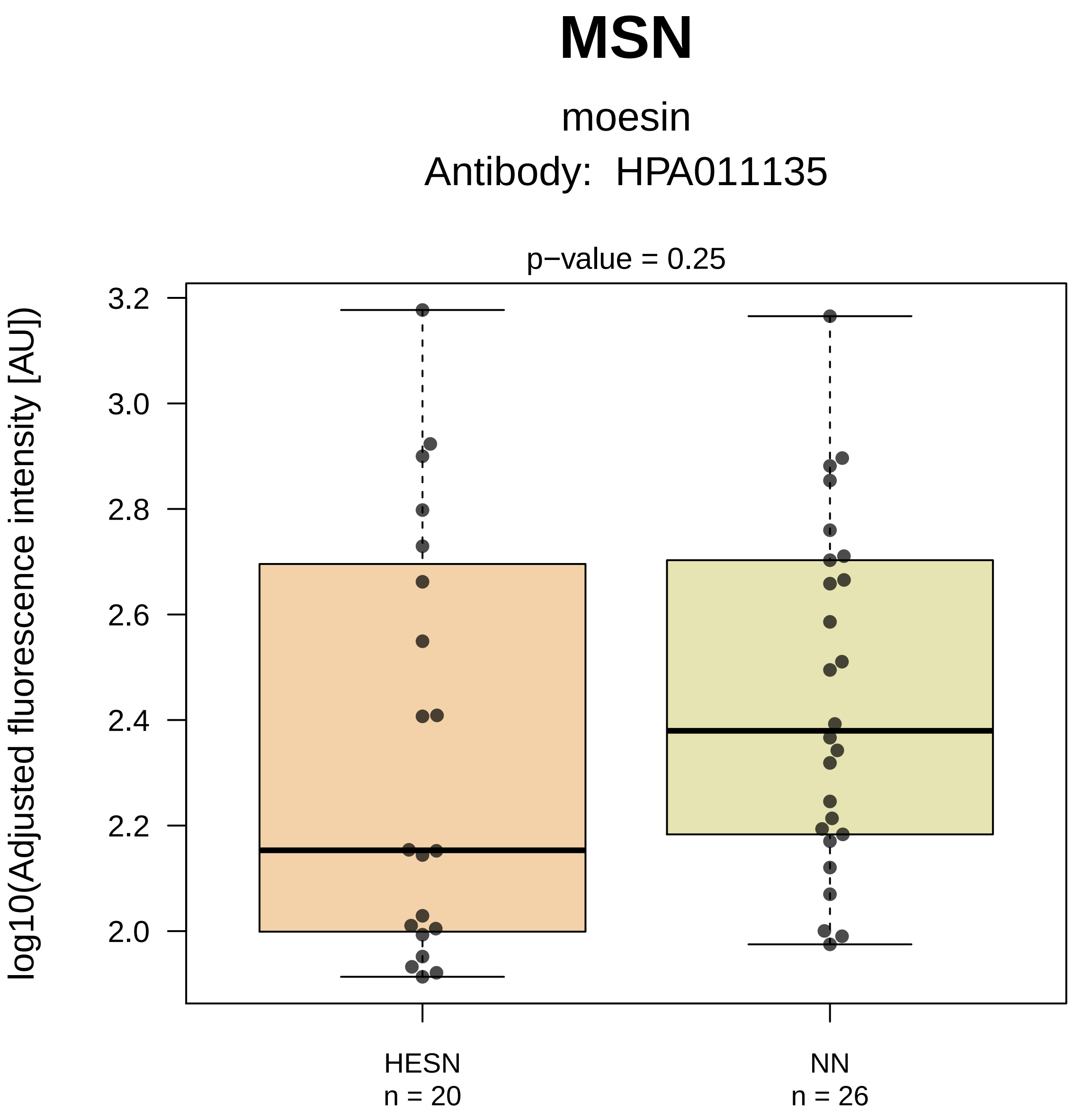
MSN
moesin
Antibody: HPA011135
p−value = 0.25
log10(Adjusted fluorescence intensity [AU])
3.2
3.0
2.8
2.6
2.4
2.2
2.0
HESN
n = 20
NN
n = 26

SPRR3

small proline rich protein 3
Antibody: HPA044467

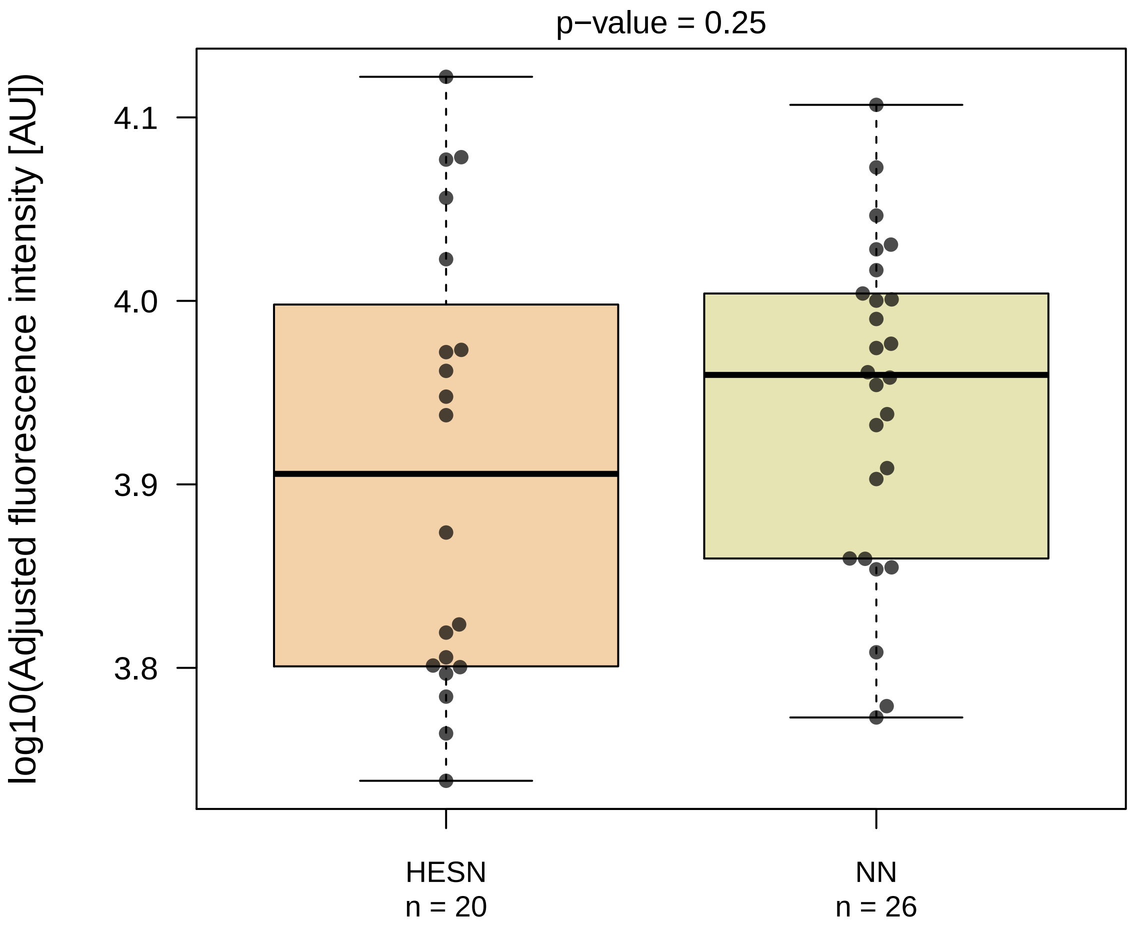

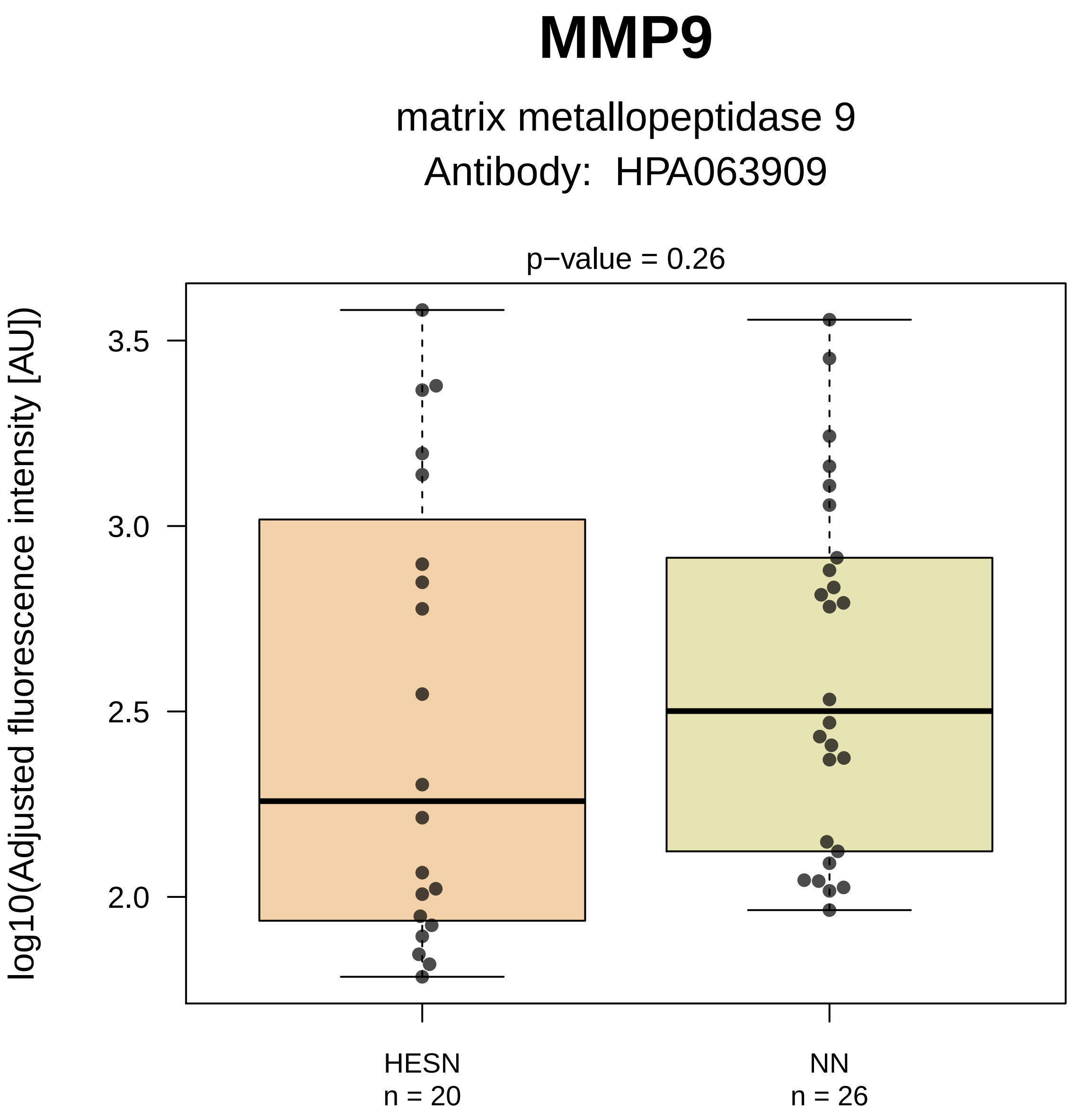

MMP9
matrix metallopeptidase 9
Antibody: HPA063909
p−value = 0.26
log10(Adjusted fluorescence intensity [AU])
3.5
3.0
2.5
2.0
HESN
n = 20
NN
n = 26

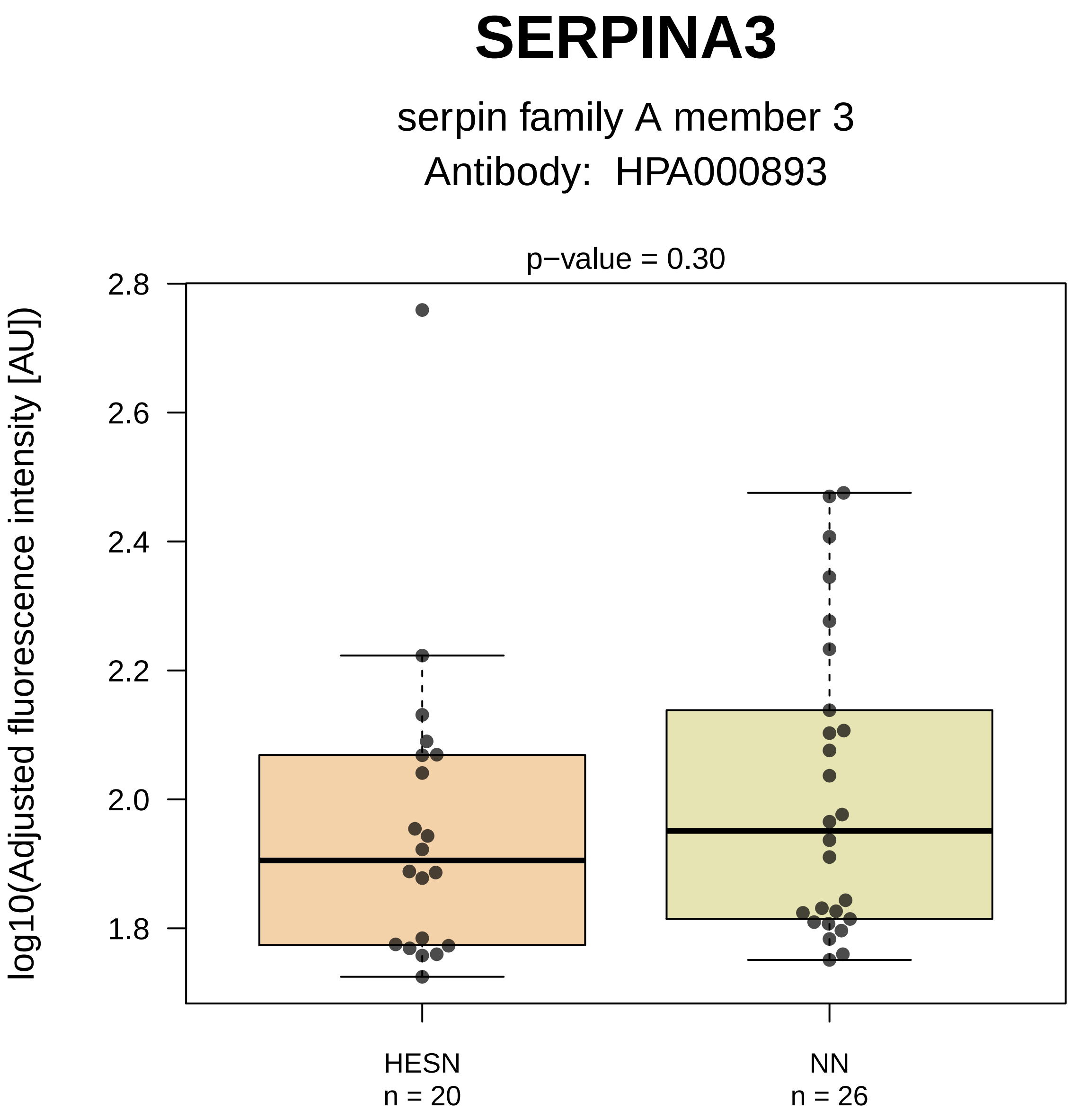

SERPINA3
serpin family A member 3
Antibody: HPA000893
p−value = 0.30
log10(Adjusted fluorescence intensity [AU])
2.8
2.6
2.4
2.2
2.0
1.8
HESN
n = 20
NN
n = 26

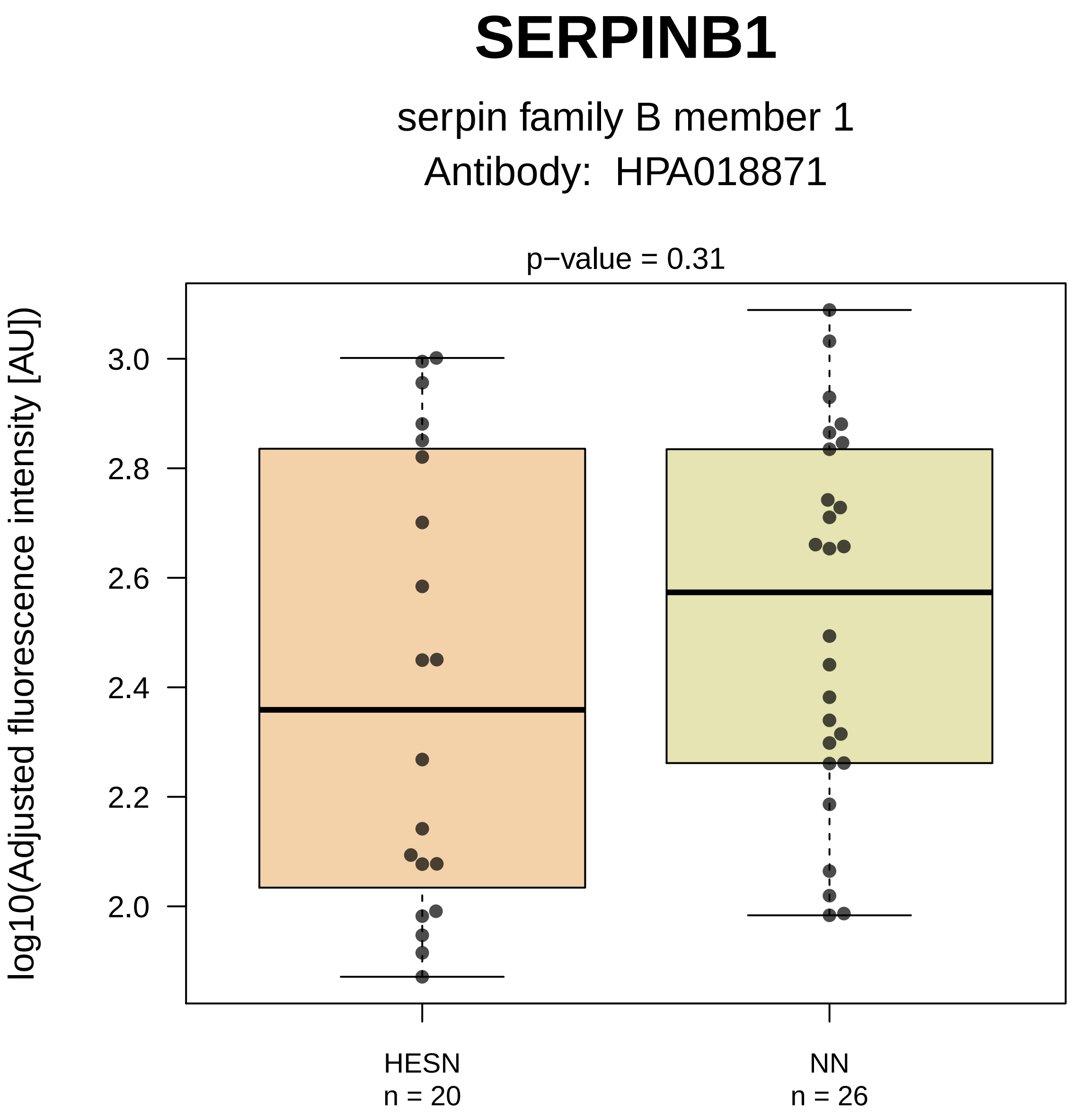
SERPINB1
serpin family B member 1
Antibody: HPA018871
p−value = 0.31
log10(Adjusted fluorescence intensity [AU])
3.0
2.8
2.6
2.4
2.2
2.0
HESN
n = 20
NN
n = 26

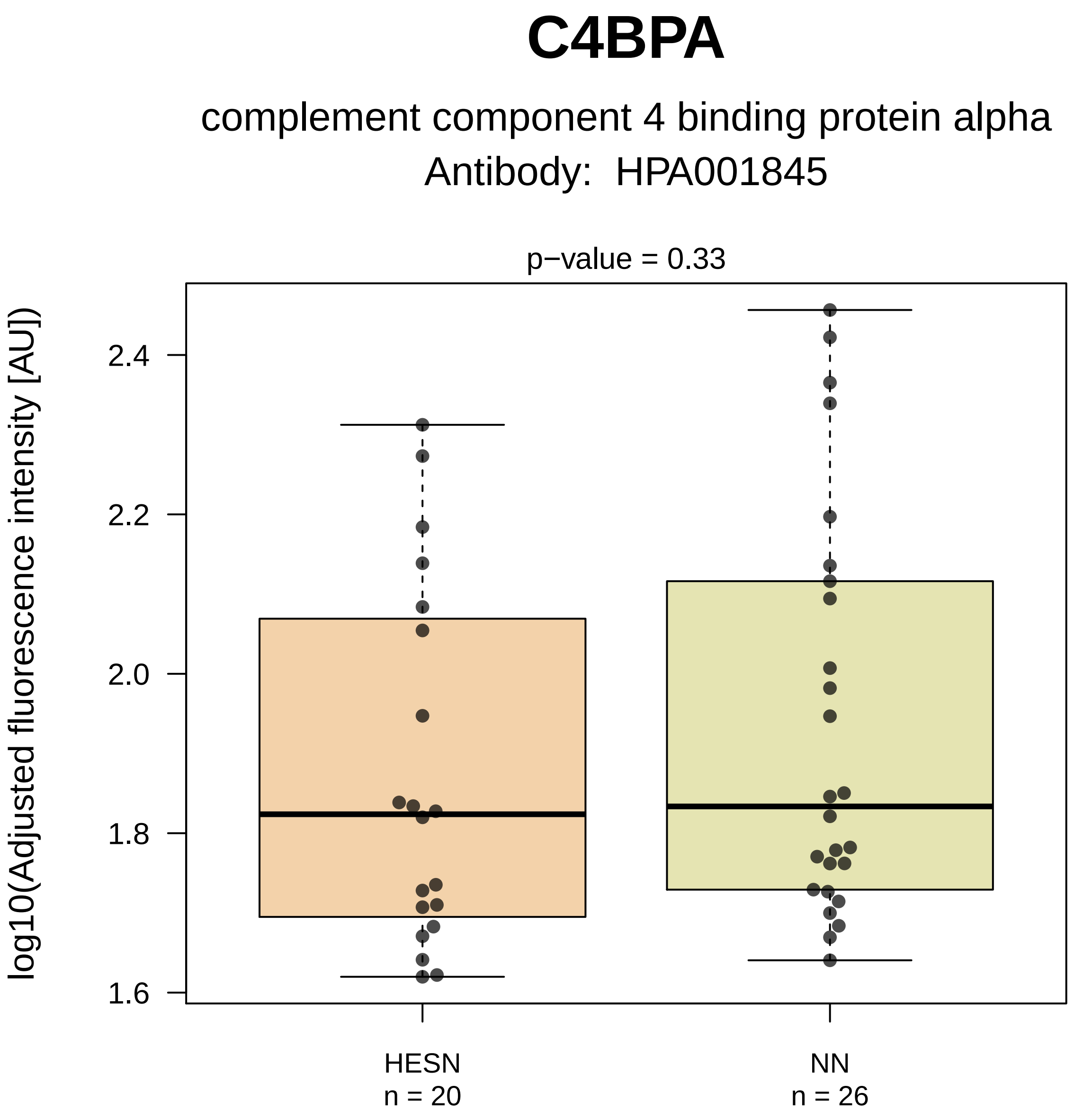

C4BPA
complement component 4 binding protein alpha
Antibody: HPA001845
p−value = 0.33
log10(Adjusted fluorescence intensity [AU])
2.4
2.2
2.0
1.8
1.6
HESN
n = 20
NN
n = 26

NCF2

neutrophil cytosolic factor 2

Antibody: HPA002327

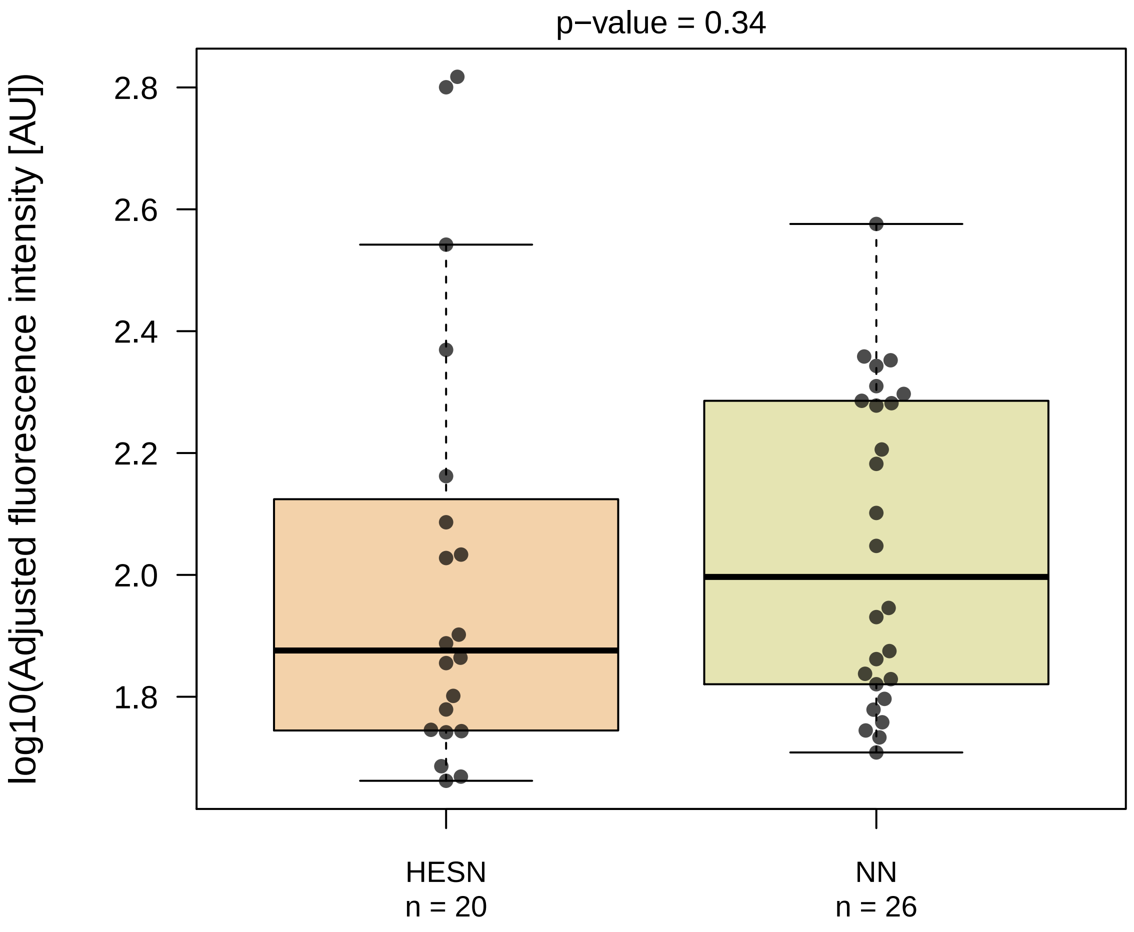

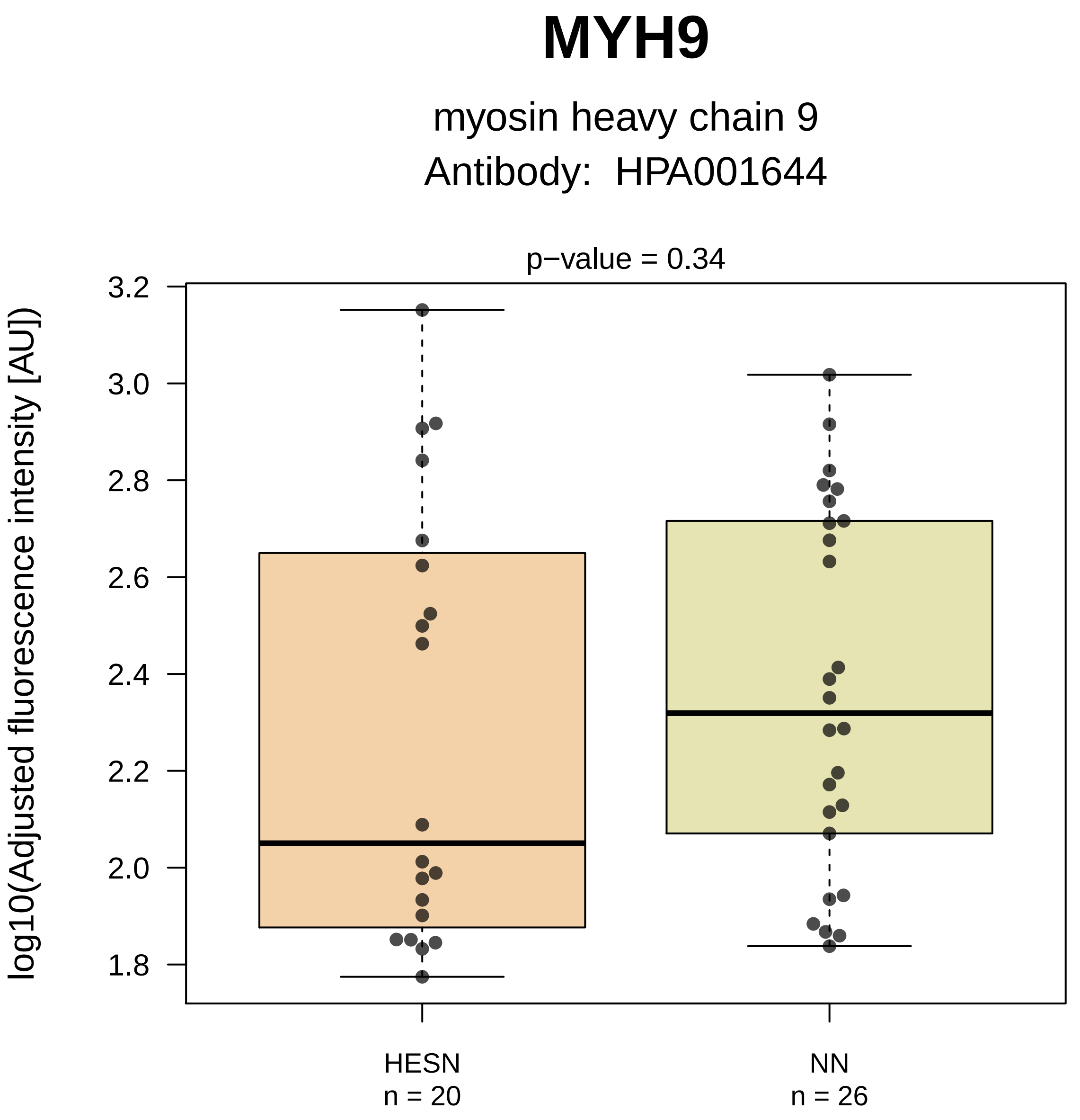
MYH9
myosin heavy chain 9
Antibody: HPA001644
p−value = 0.34
log10(Adjusted fluorescence intensity [AU])
3.2
3.0
2.8
2.6
2.4
2.2
2.0
1.8
HESN
n = 20
NN
n = 26

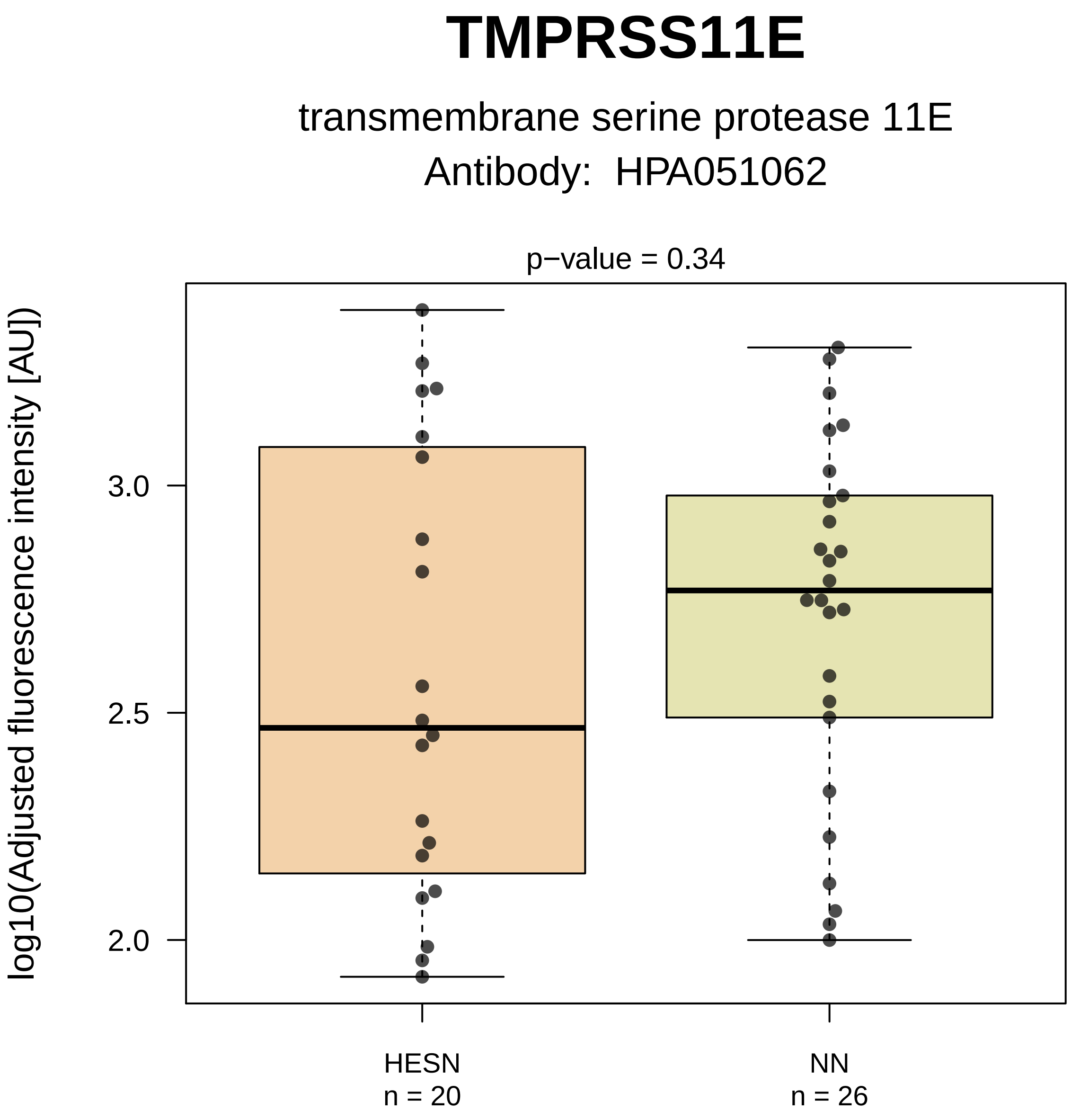
TMPRSS11E
transmembrane serine protease 11E
Antibody: HPA051062
p−value = 0.34
log10(Adjusted fluorescence intensity [AU])
3.0
2.5
2.0
HESN
n = 20
NN
n = 26

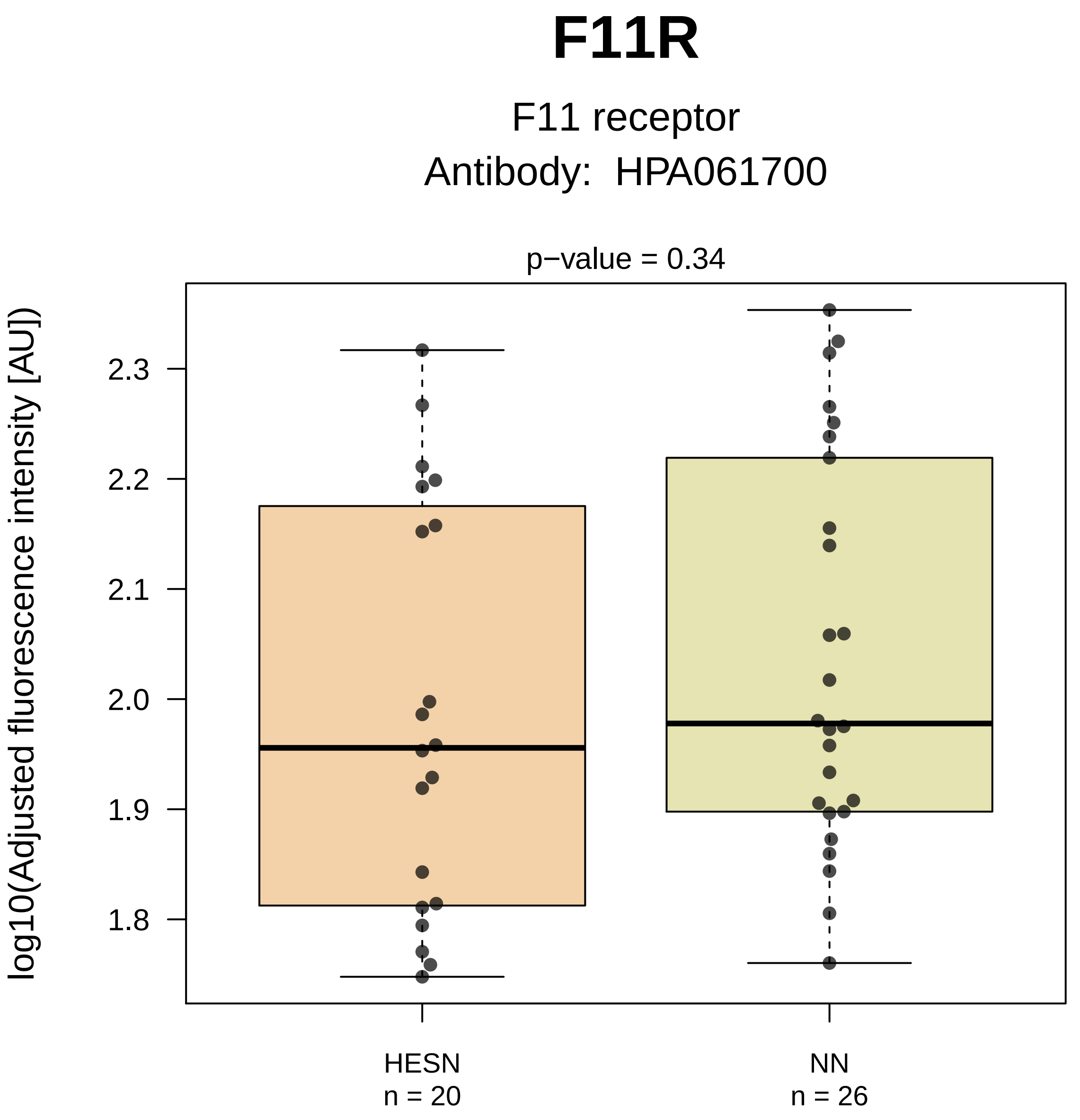
F11R
F11 receptor
Antibody: HPA061700
p−value = 0.34
log10(Adjusted fluorescence intensity [AU])
2.3
2.2
2.1
2.0
1.9
1.8
HESN
n = 20
NN
n = 26

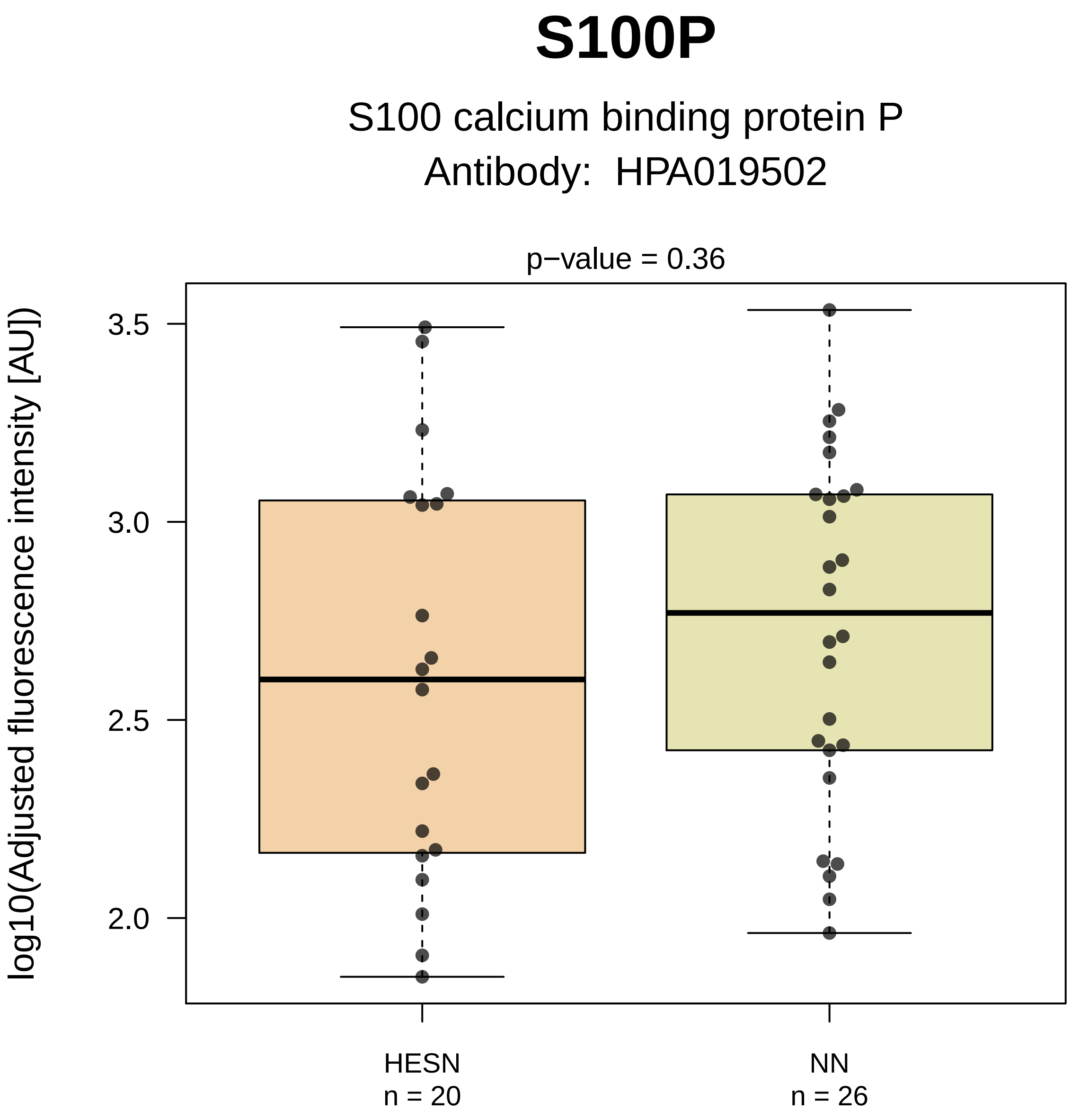

S100P
S100 calcium binding protein P
Antibody: HPA019502
p−value = 0.36
log10(Adjusted fluorescence intensity [AU])
3.5
3.0
2.5
2.0
HESN
n = 20
NN
n = 26

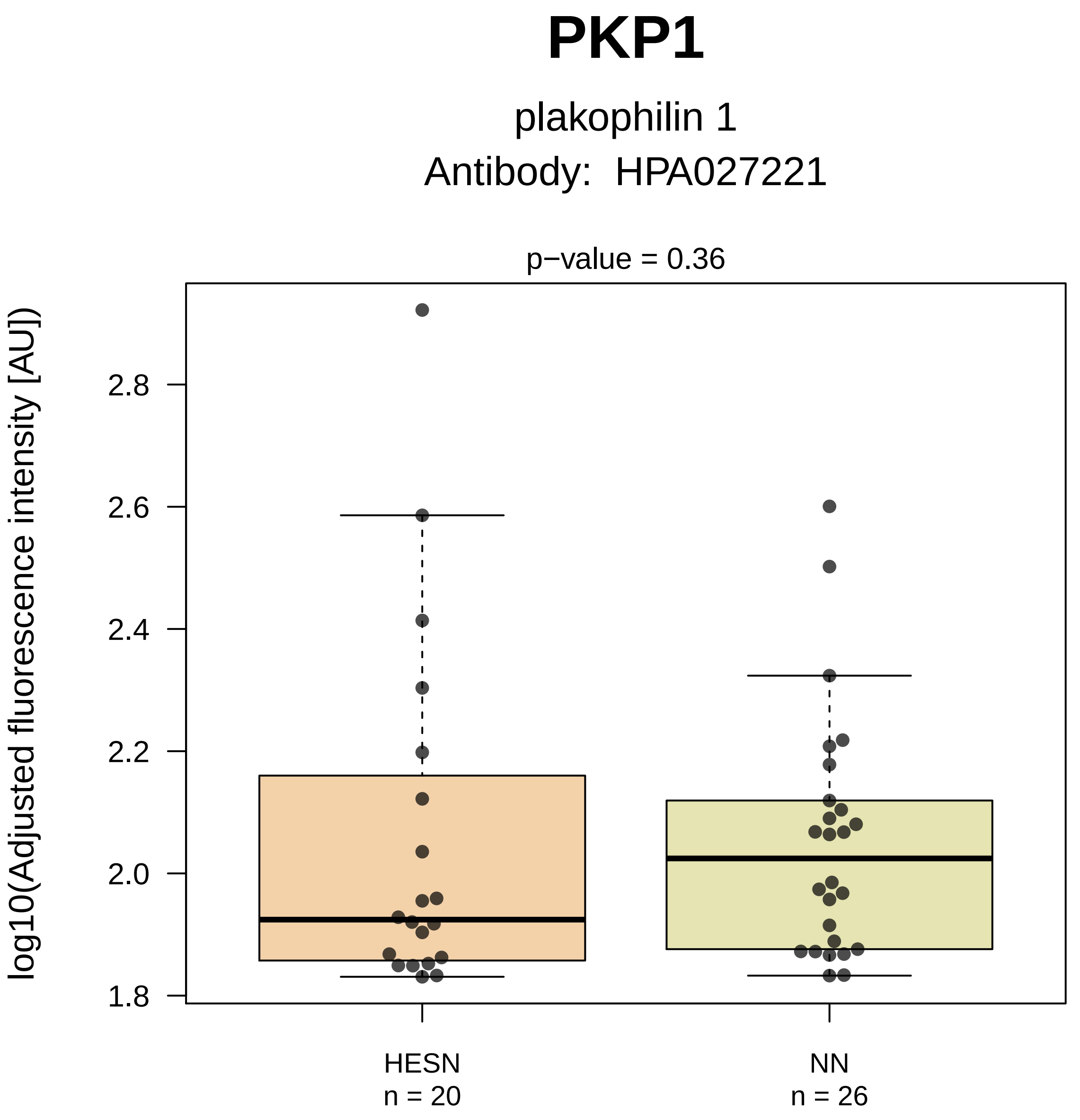
PKP1
plakophilin 1
Antibody: HPA027221
p−value = 0.36
log10(Adjusted fluorescence intensity [AU])
2.8
2.6
2.4
2.2
2.0
1.8
HESN
n = 20
NN
n = 26

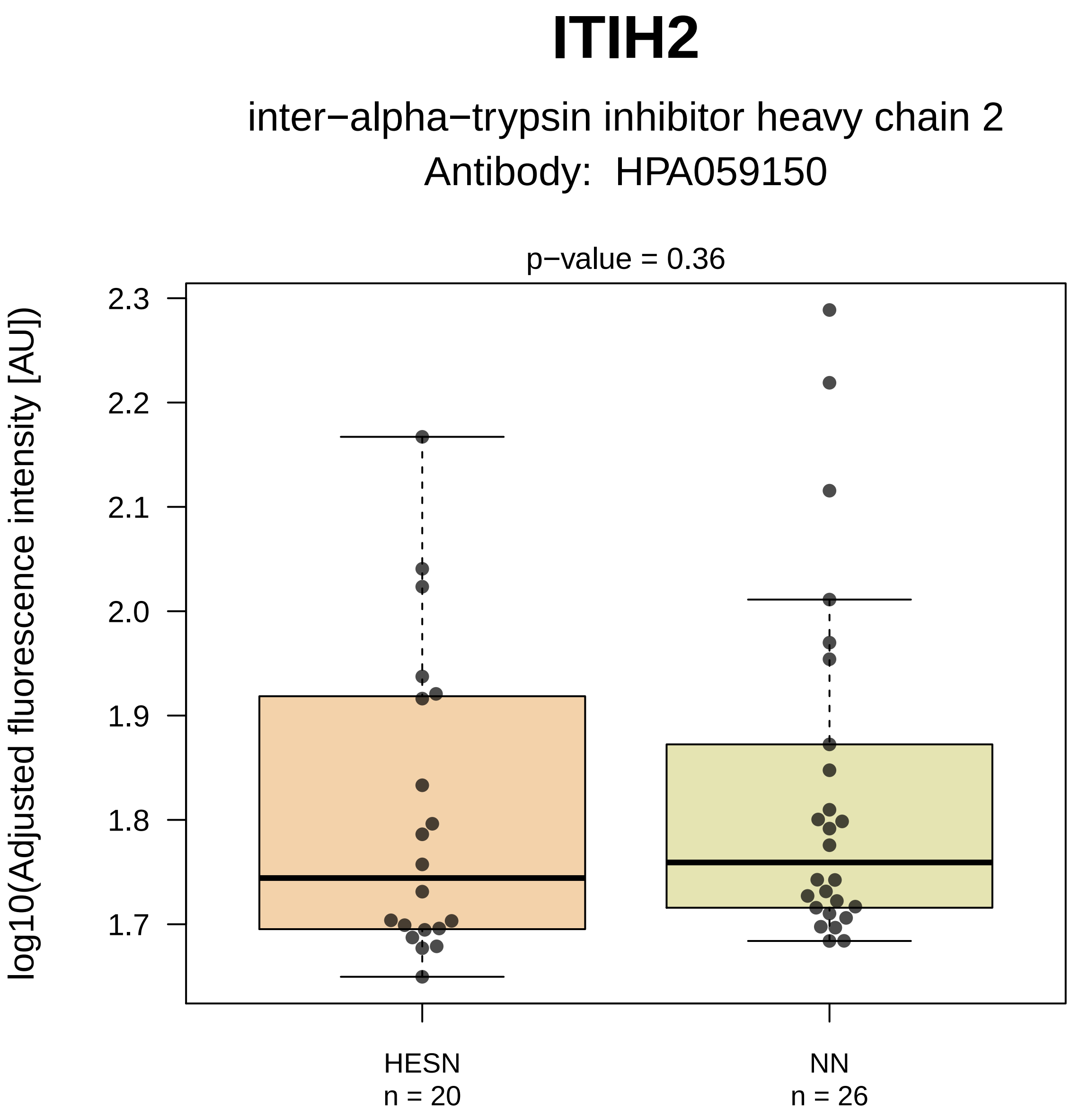
ITIH2
inter−alpha−trypsin inhibitor heavy chain 2
Antibody: HPA059150
p−value = 0.36
log10(Adjusted fluorescence intensity [AU])
2.3
2.2
2.1
2.0
1.9
1.8
1.7
HESN
n = 20
NN
n = 26

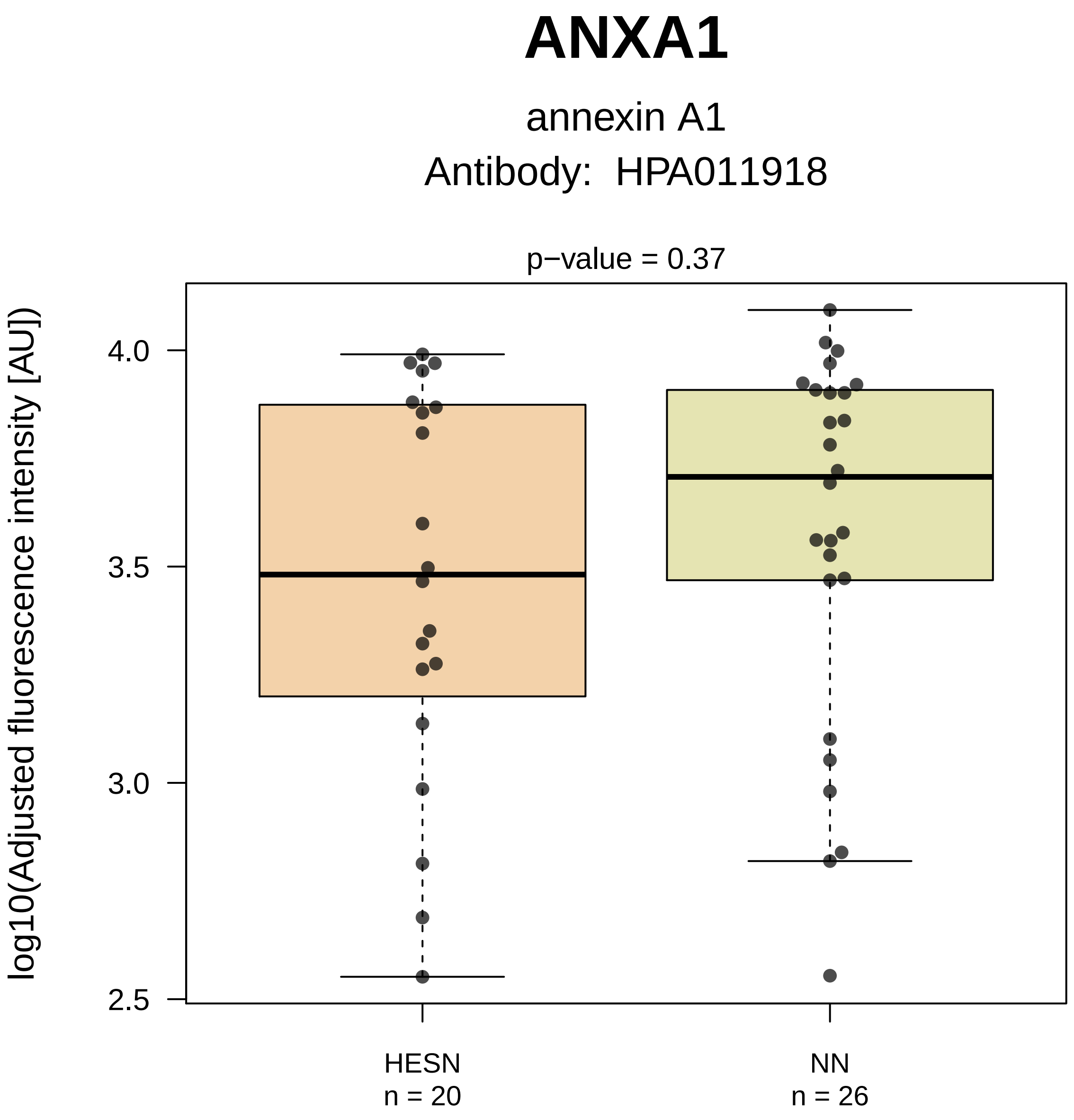
ANXA1
annexin A1
Antibody: HPA011918
p−value = 0.37
log10(Adjusted fluorescence intensity [AU])
4.0
3.5
3.0
2.5
HESN
n = 20
NN
n = 26

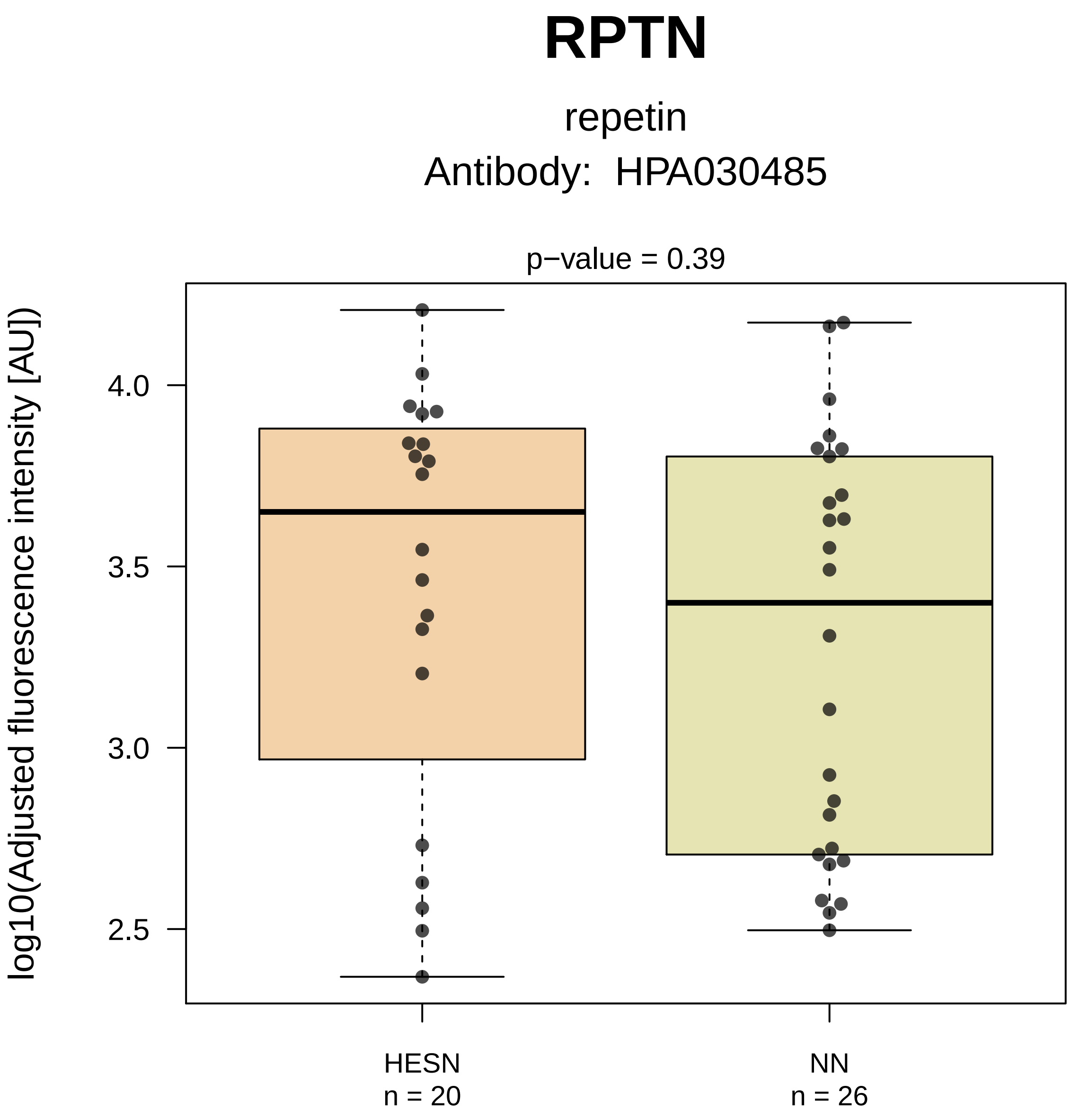
RPTN
repetin
Antibody: HPA030485
p−value = 0.39
log10(Adjusted fluorescence intensity [AU])
4.0
3.5
3.0
2.5
HESN
n = 20
NN
n = 26

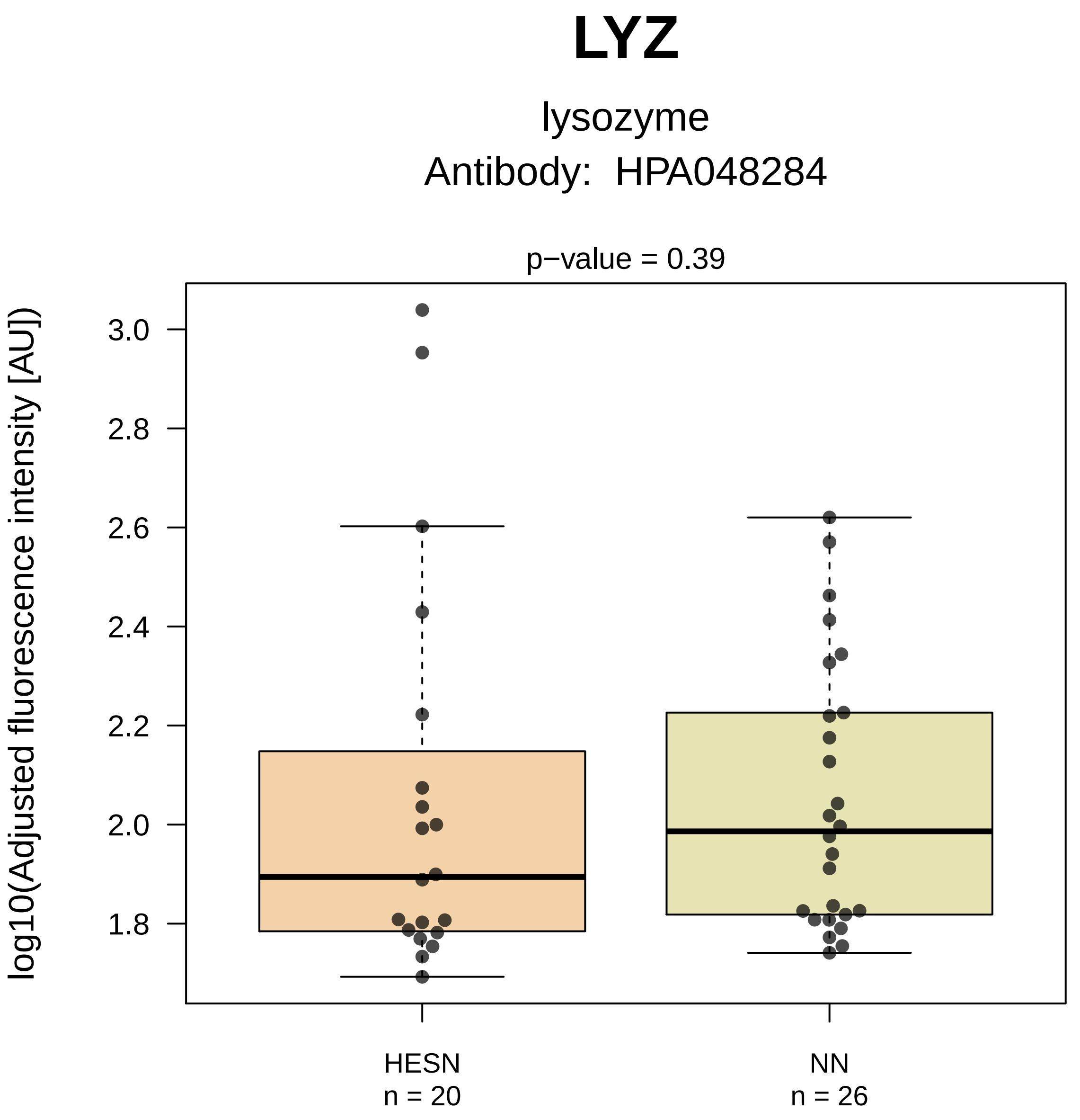

LYZ
lysozyme
Antibody: HPA048284
p−value = 0.39
log10(Adjusted fluorescence intensity [AU])
3.0
2.8
2.6
2.4
2.2
2.0
1.8
HESN
n = 20
NN
n = 26

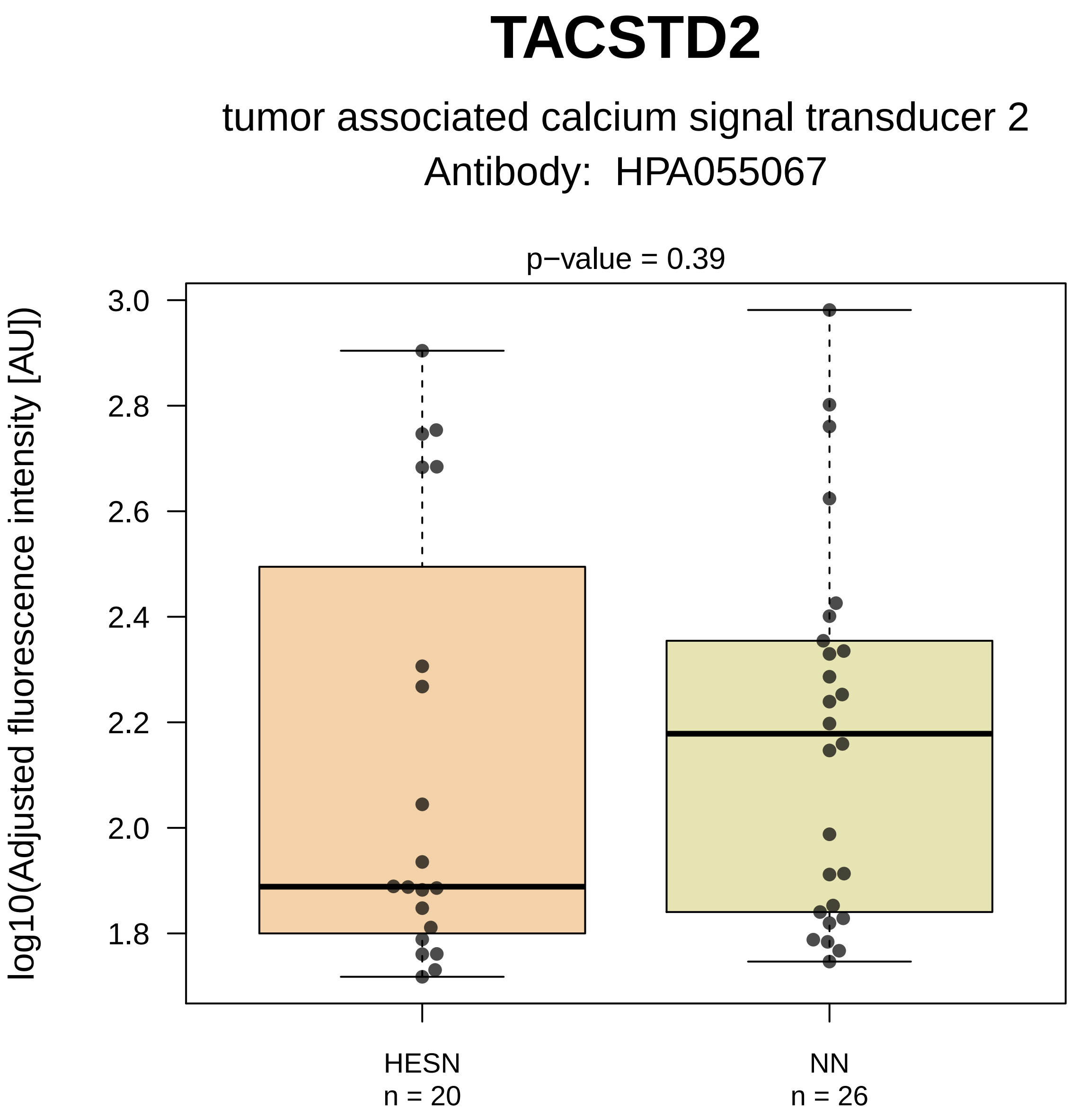
TACSTD2
tumor associated calcium signal transducer 2
Antibody: HPA055067
p−value = 0.39
log10(Adjusted fluorescence intensity [AU])
3.0
2.8
2.6
2.4
2.2
2.0
1.8
HESN
n = 20
NN
n = 26

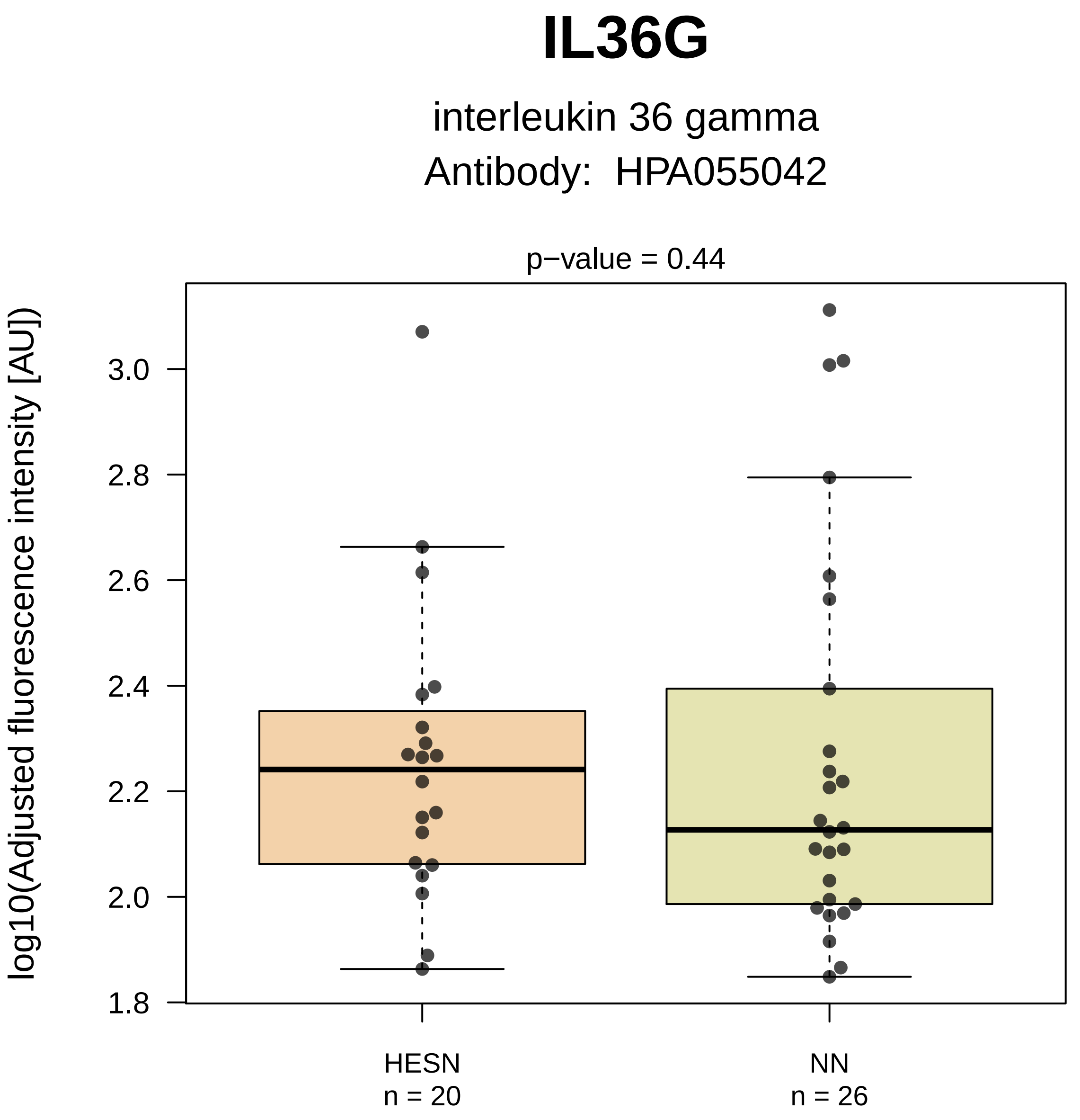
IL36G
interleukin 36 gamma
Antibody: HPA055042
p−value = 0.44
log10(Adjusted fluorescence intensity [AU])
3.0
2.8
2.6
2.4
2.2
2.0
1.8
HESN
n = 20
NN
n = 26

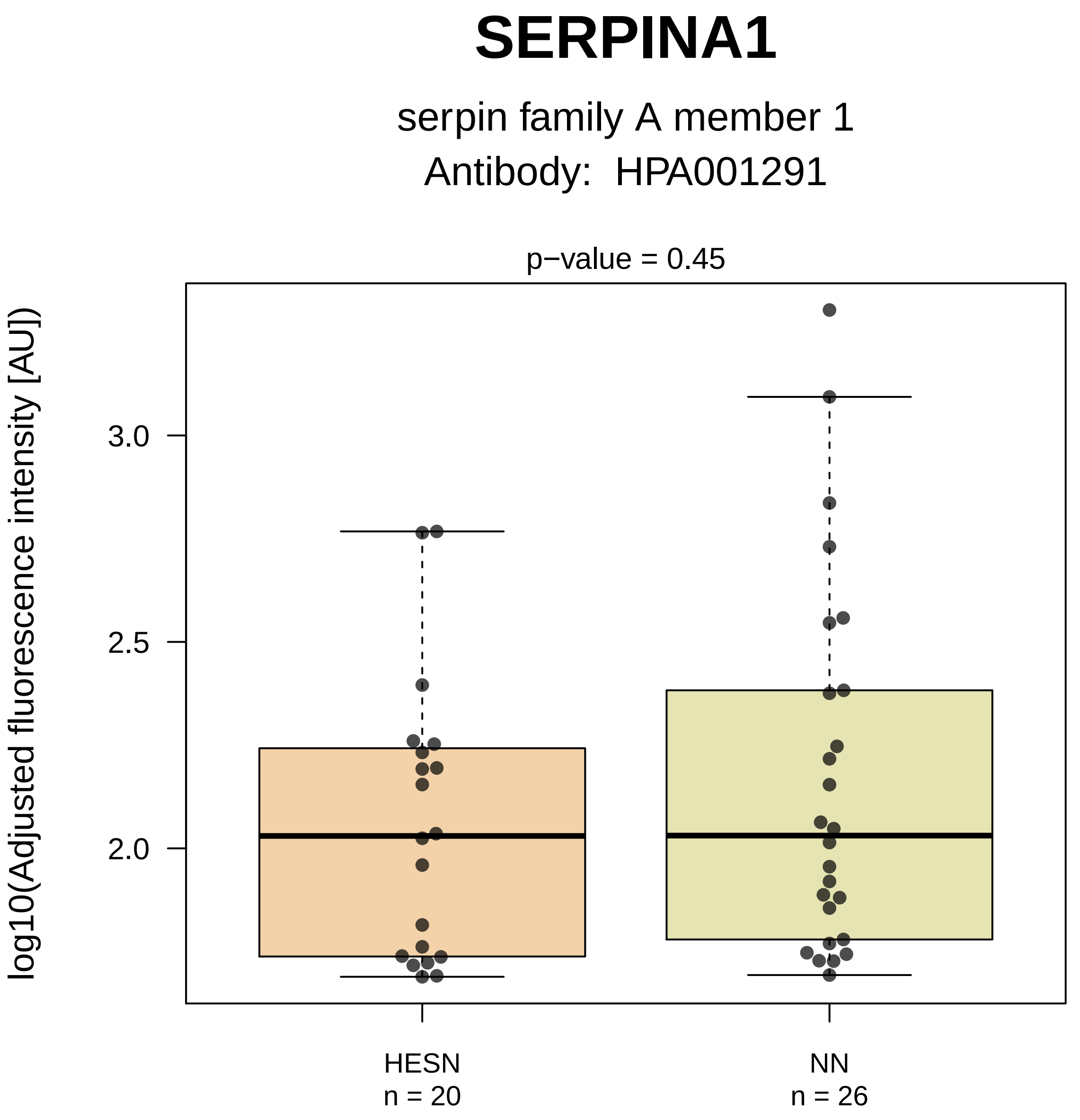
SERPINA1
serpin family A member 1
Antibody: HPA001291
p−value = 0.45
log10(Adjusted fluorescence intensity [AU])
3.0
2.5
2.0
HESN
n = 20
NN
n = 26

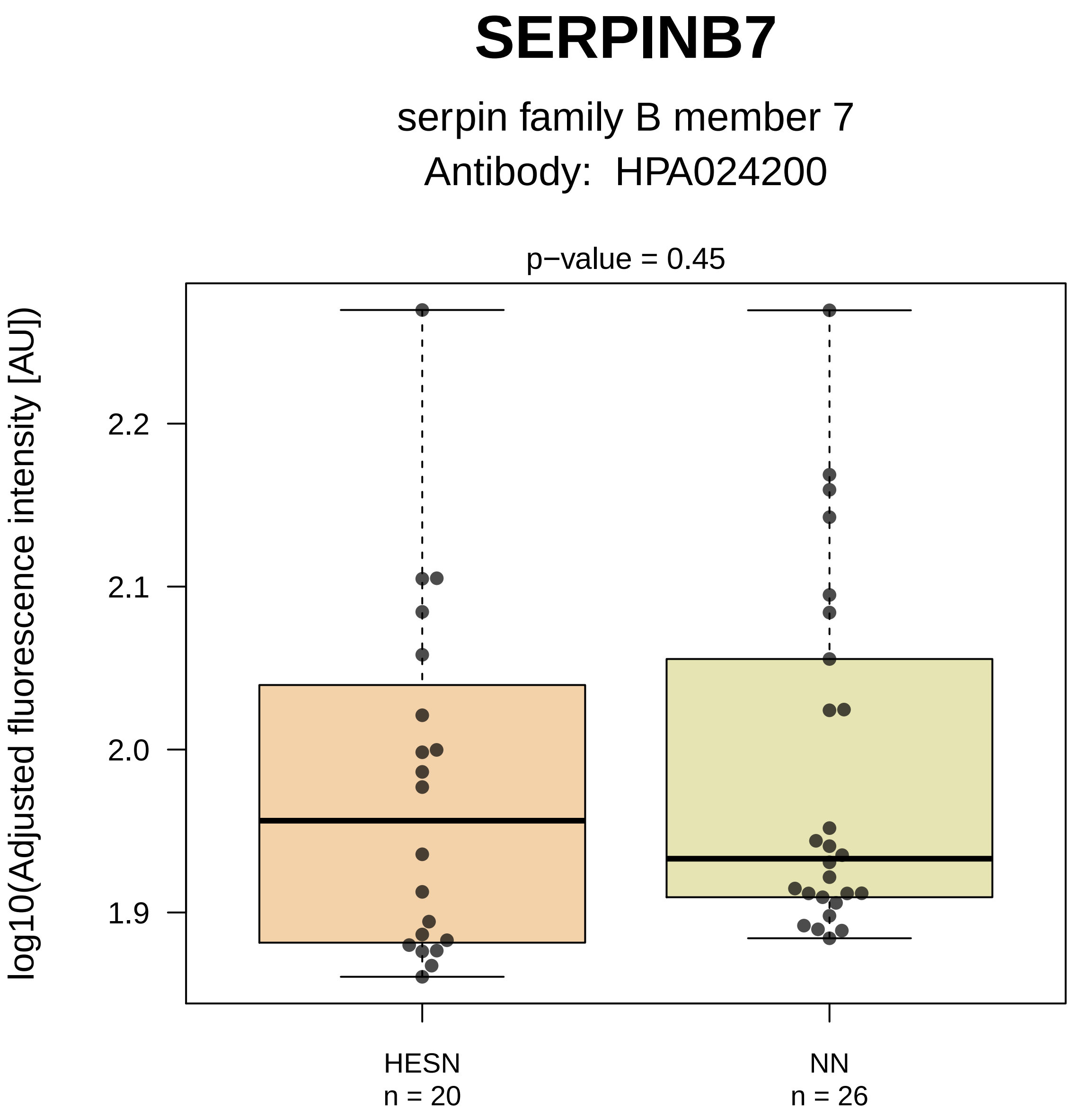
SERPINB7
serpin family B member 7
Antibody: HPA024200
p−value = 0.45
log10(Adjusted fluorescence intensity [AU])
2.2
2.1
2.0
1.9
HESN
n = 20
NN
n = 26

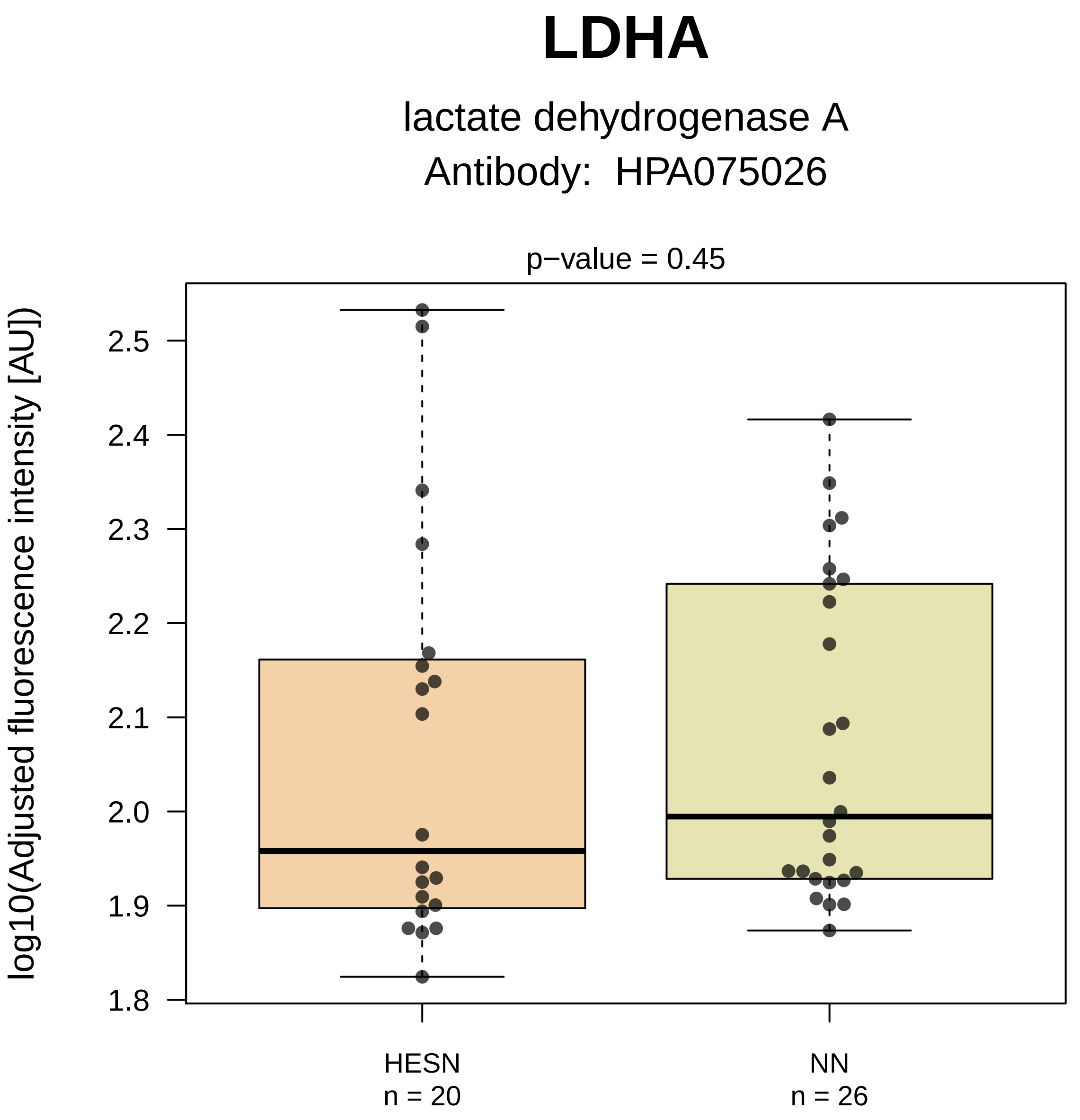
LDHA
lactate dehydrogenase A
Antibody: HPA075026
p−value = 0.45
log10(Adjusted fluorescence intensity [AU])
2.5
2.4
2.3
2.2
2.1
2.0
1.9
1.8
HESN
n = 20
NN
n = 26

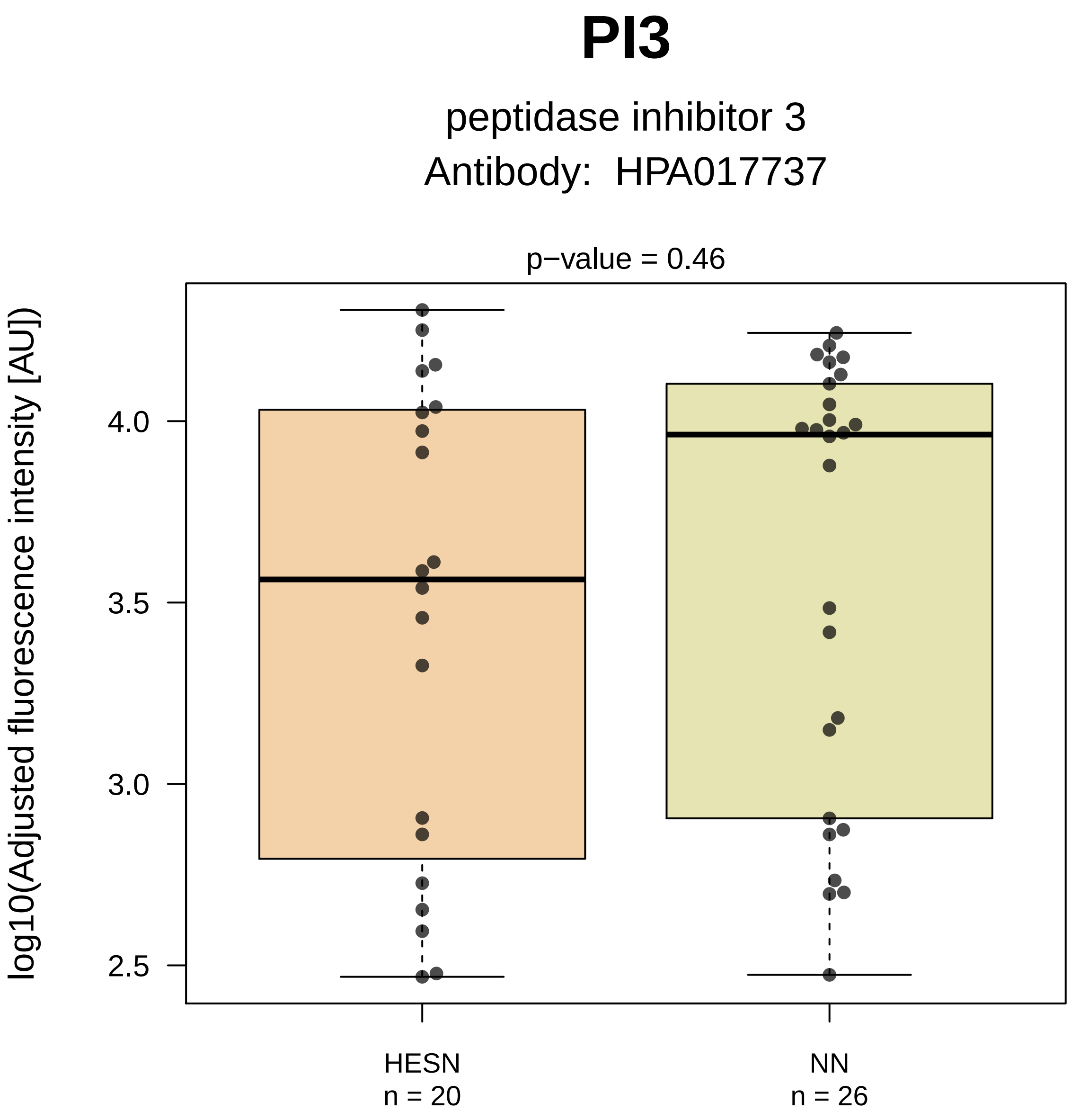
PI3
peptidase inhibitor 3
Antibody: HPA017737
p−value = 0.46
log10(Adjusted fluorescence intensity [AU])
4.0
3.5
3.0
2.5
HESN
n = 20
NN
n = 26

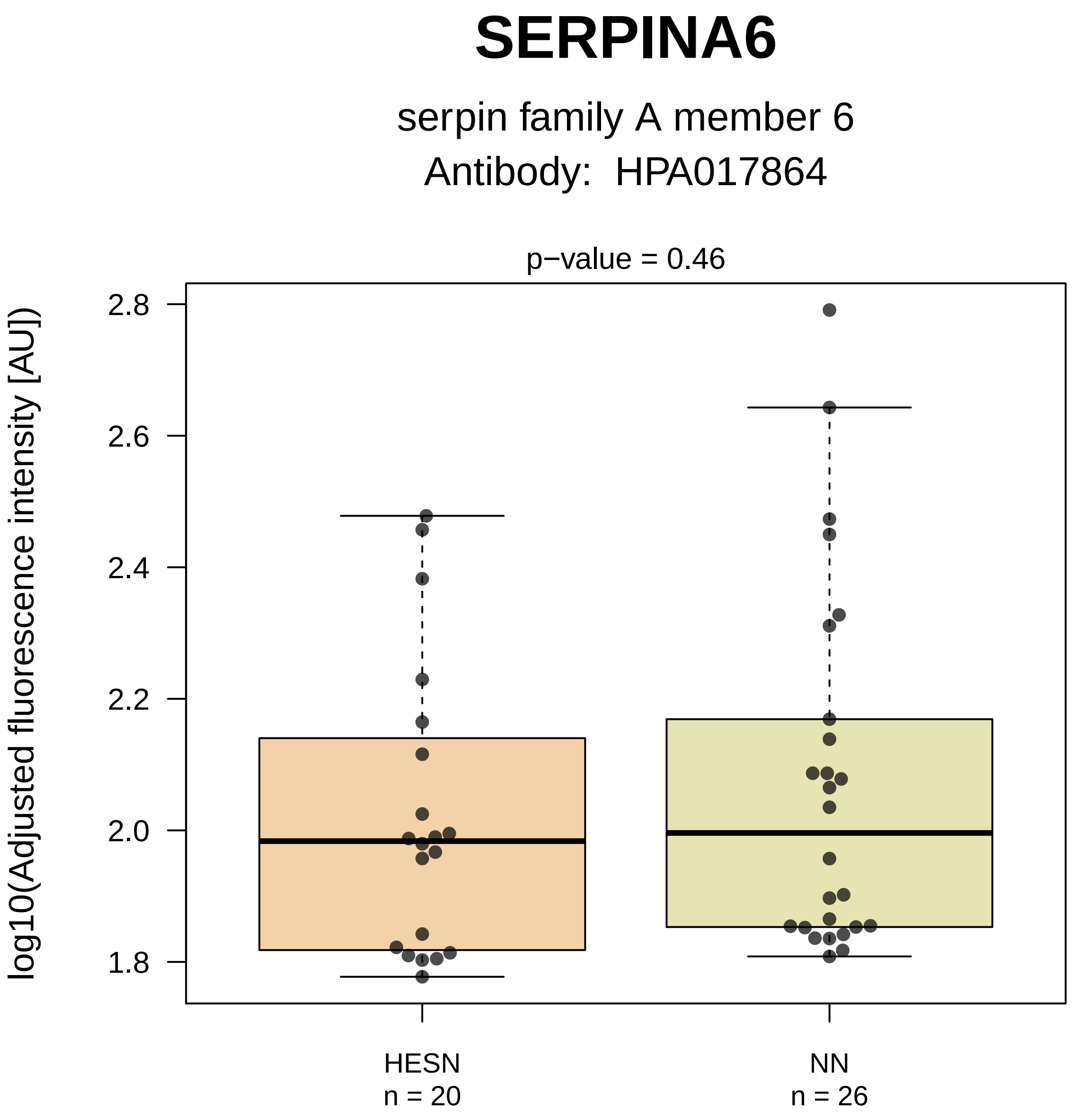
SERPINA6
serpin family A member 6
Antibody: HPA017864
p−value = 0.46
log10(Adjusted fluorescence intensity [AU])
2.8
2.6
2.4
2.2
2.0
1.8
HESN
n = 20
NN
n = 26

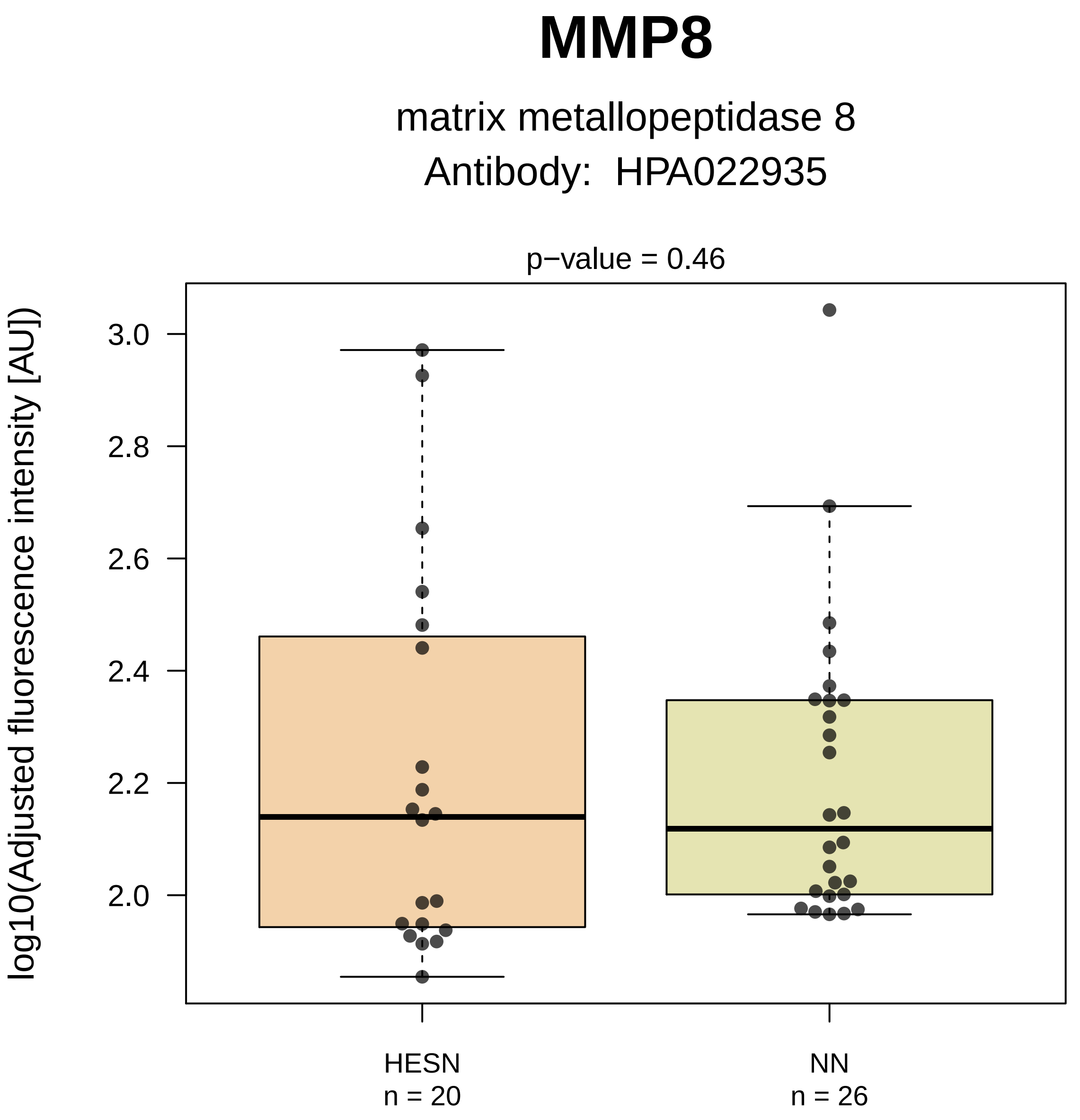
MMP8
matrix metallopeptidase 8
Antibody: HPA022935
p−value = 0.46
log10(Adjusted fluorescence intensity [AU])
3.0
2.8
2.6
2.4
2.2
2.0
HESN
n = 20
NN
n = 26

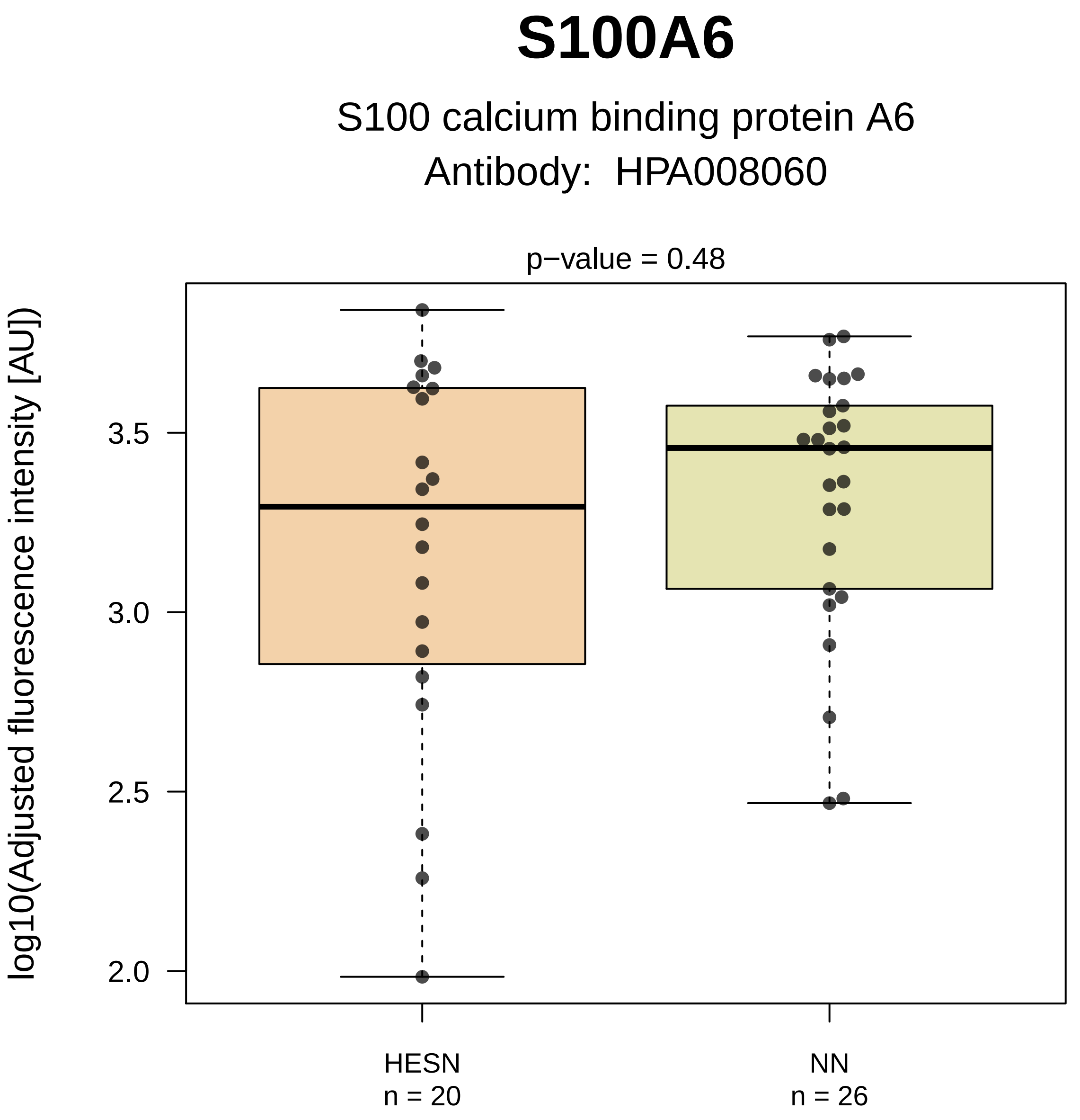
S100A6
S100 calcium binding protein A6
Antibody: HPA008060
p−value = 0.48
log10(Adjusted fluorescence intensity [AU])
3.5
3.0
2.5
2.0
HESN
n = 20
NN
n = 26

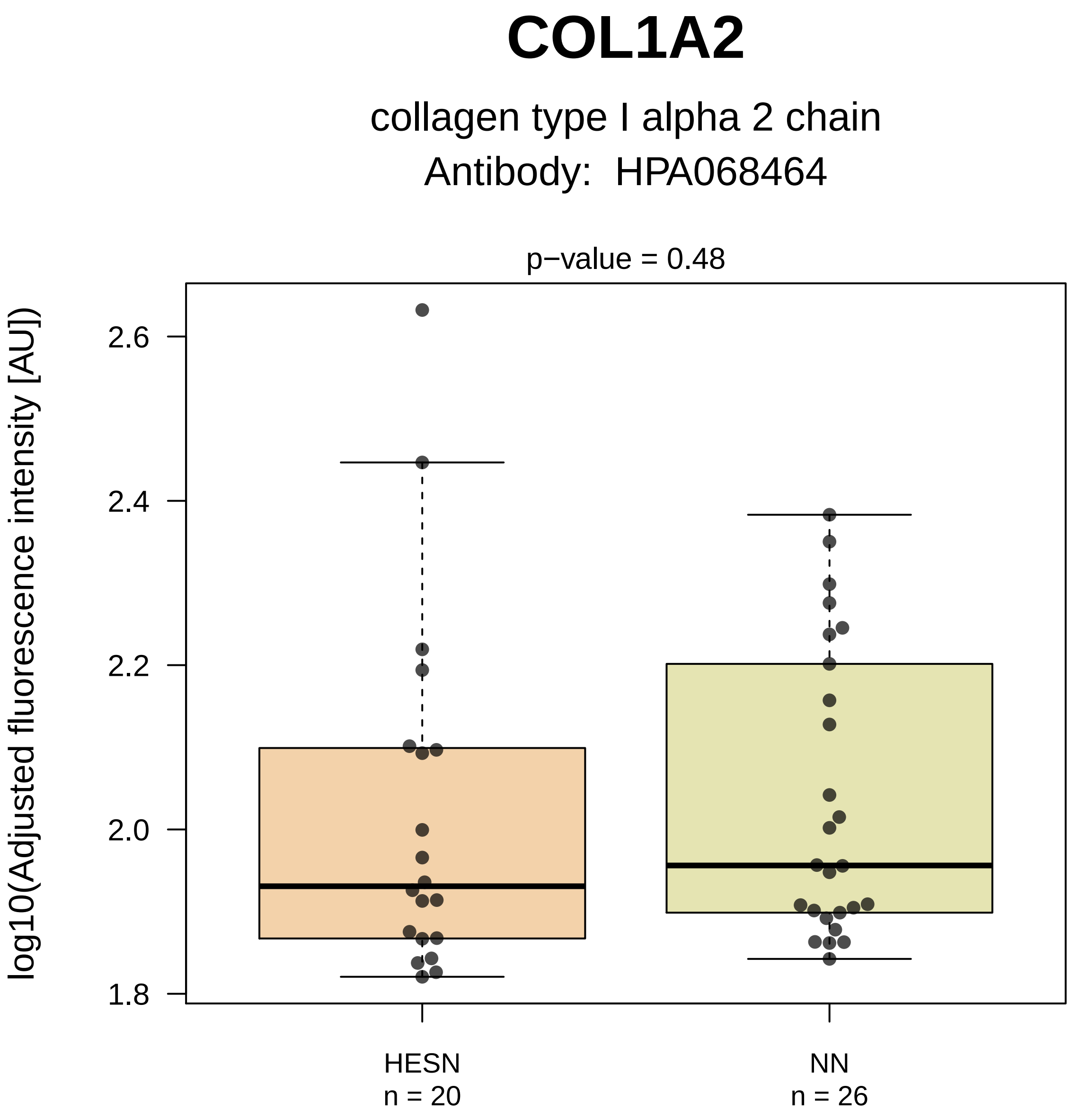

COL1A2
collagen type I alpha 2 chain
Antibody: HPA068464
p−value = 0.48
log10(Adjusted fluorescence intensity [AU])
2.6
2.4
2.2
2.0
1.8
HESN
n = 20
NN
n = 26

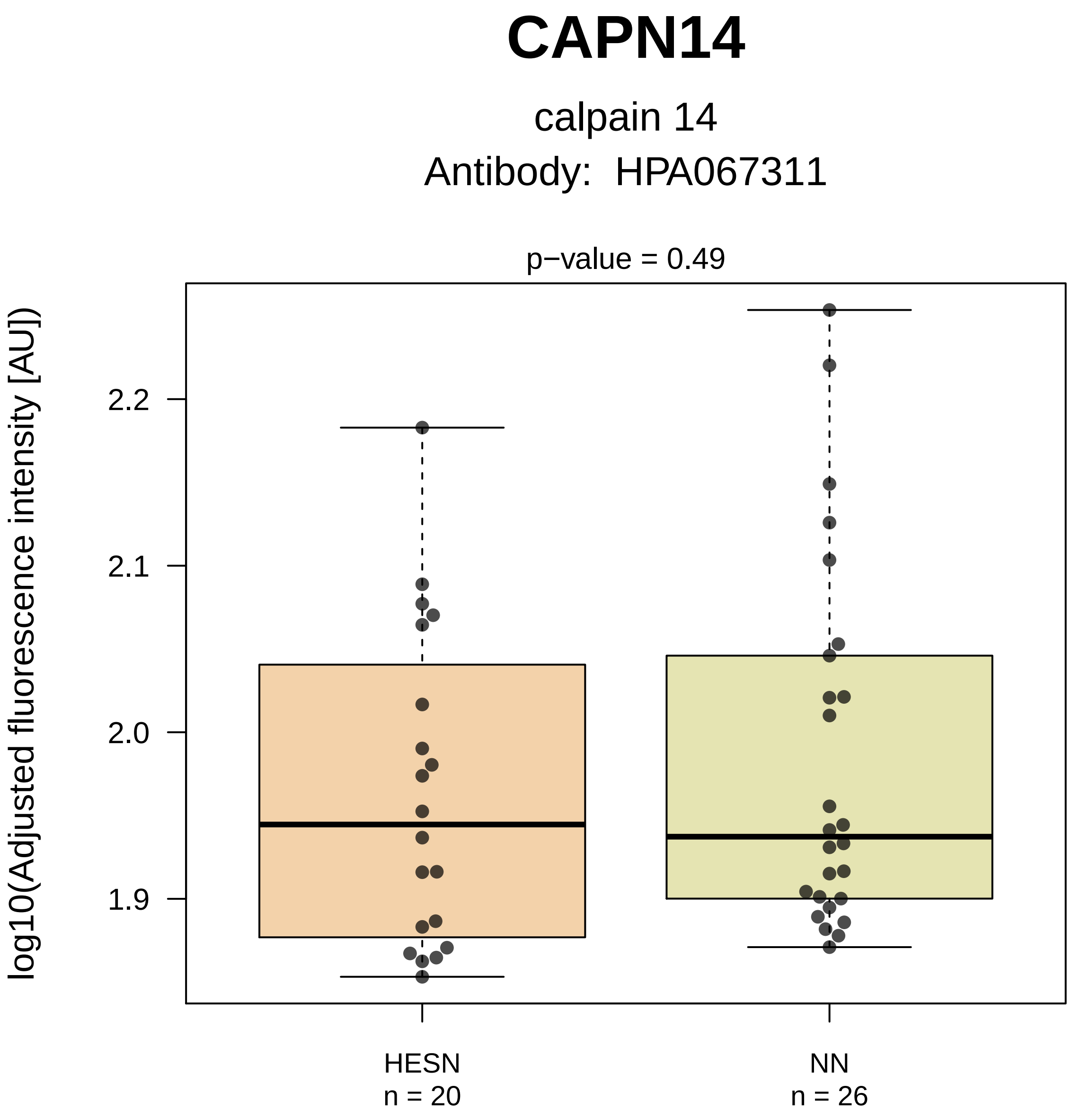
CAPN14
calpain 14
Antibody: HPA067311
p−value = 0.49
log10(Adjusted fluorescence intensity [AU])
2.2
2.1
2.0
1.9
HESN
n = 20
NN
n = 26

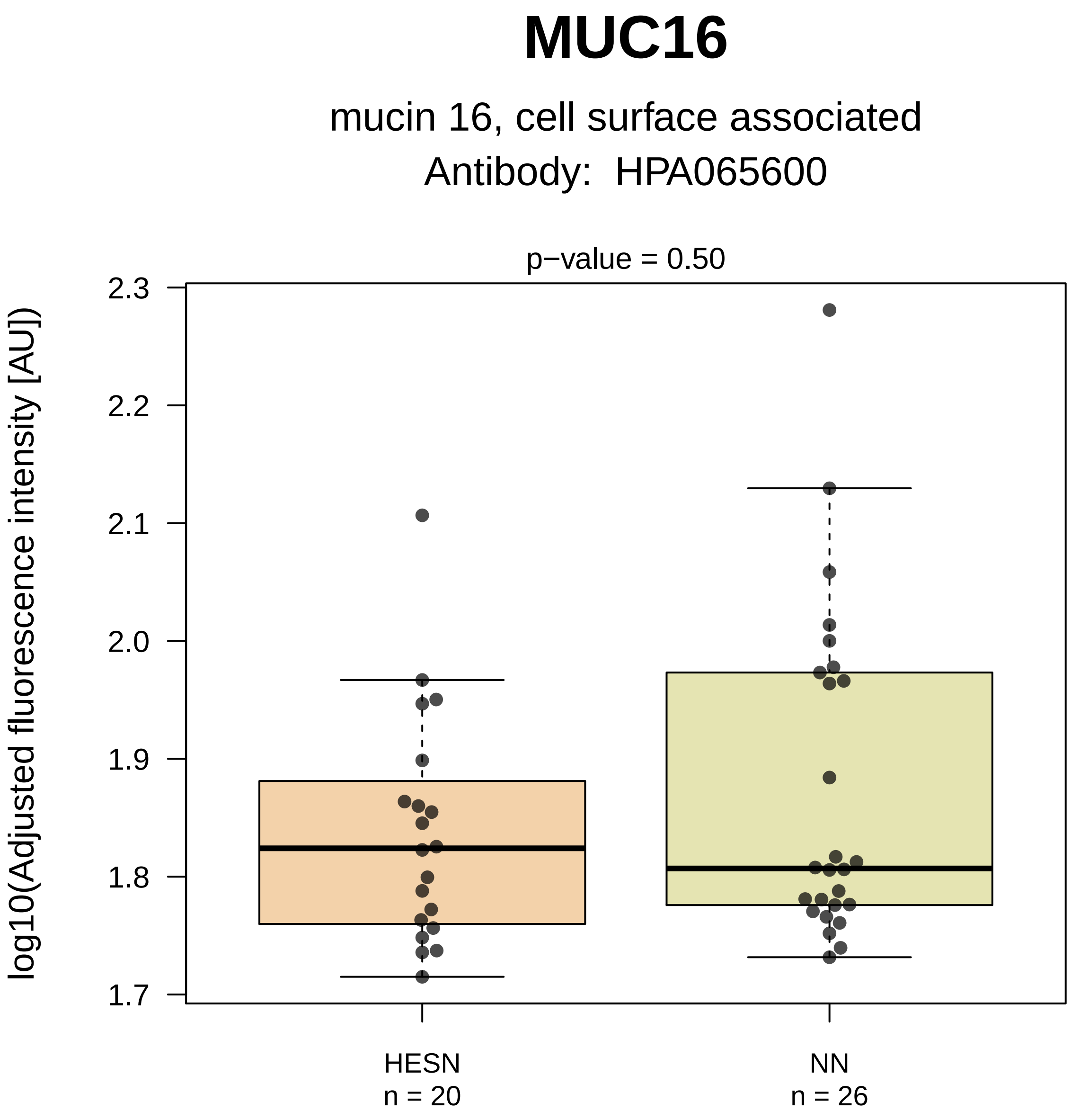

MUC16
mucin 16, cell surface associated
Antibody: HPA065600
p−value = 0.50
log10(Adjusted fluorescence intensity [AU])
2.3
2.2
2.1
2.0
1.9
1.8
1.7
HESN
n = 20
NN
n = 26

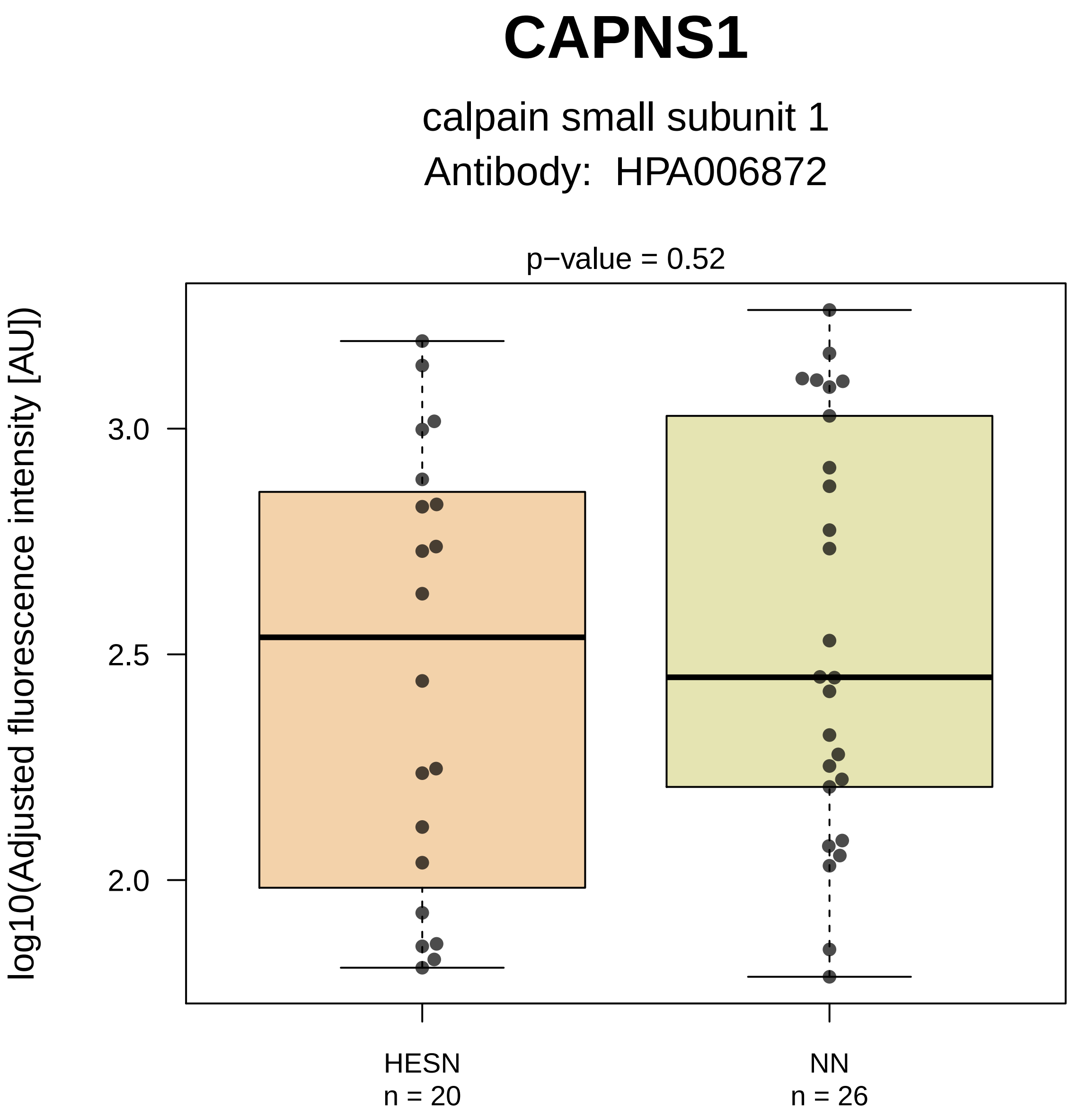
CAPNS1
calpain small subunit 1
Antibody: HPA006872
p−value = 0.52
log10(Adjusted fluorescence intensity [AU])
3.0
2.5
2.0
HESN
n = 20
NN
n = 26

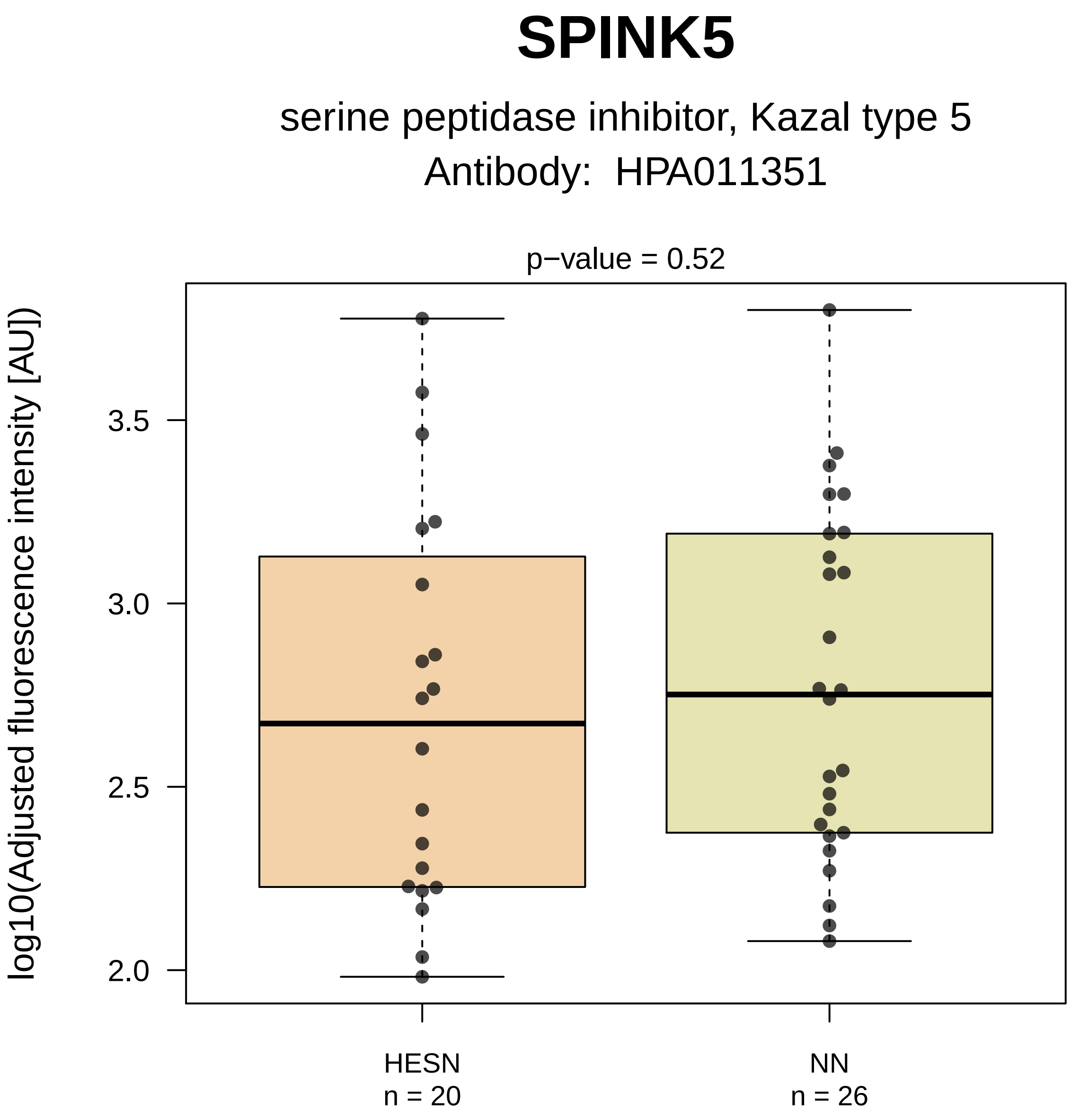

SPINK5
serine peptidase inhibitor, Kazal type 5
Antibody: HPA011351
p−value = 0.52
log10(Adjusted fluorescence intensity [AU])
3.5
3.0
2.5
2.0
HESN
n = 20
NN
n = 26

CXCL10

C−X−C motif chemokine ligand 10

Antibody: HPA029858

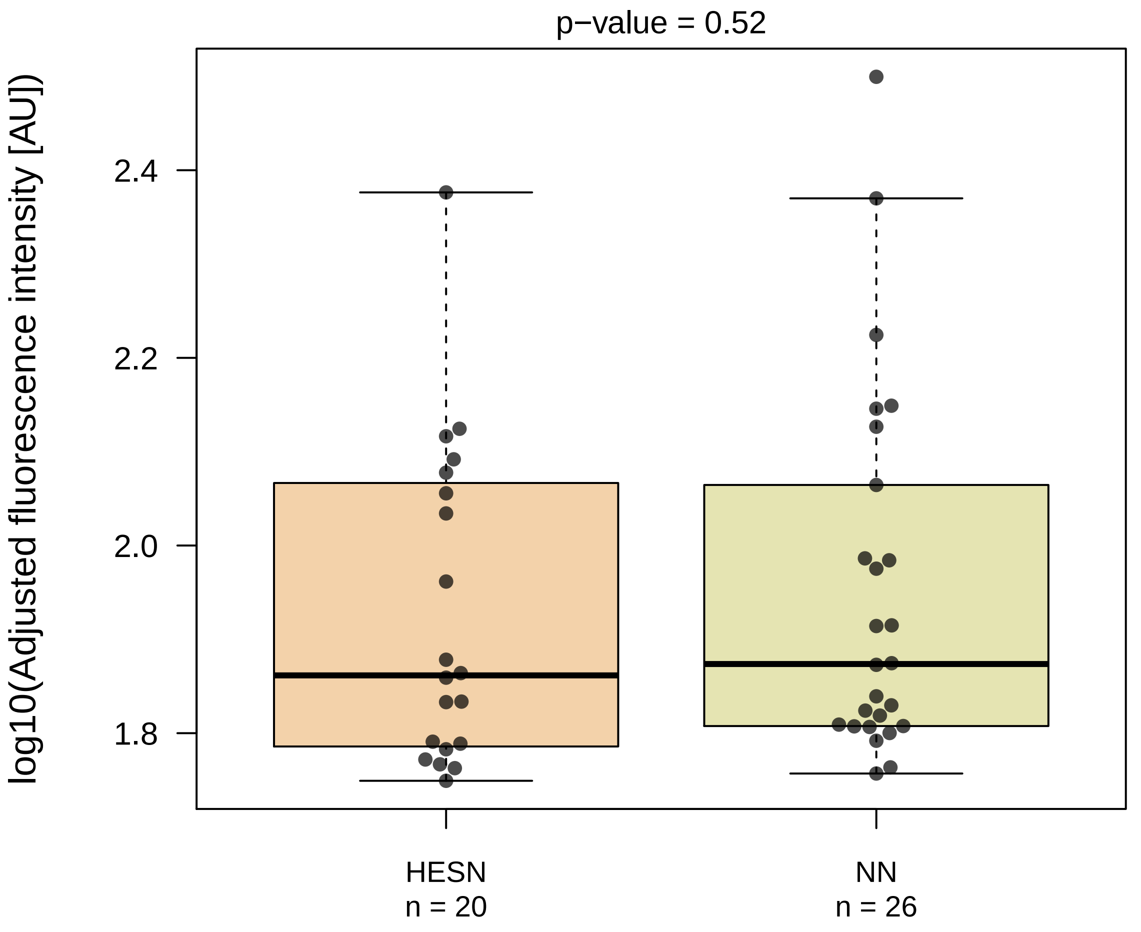

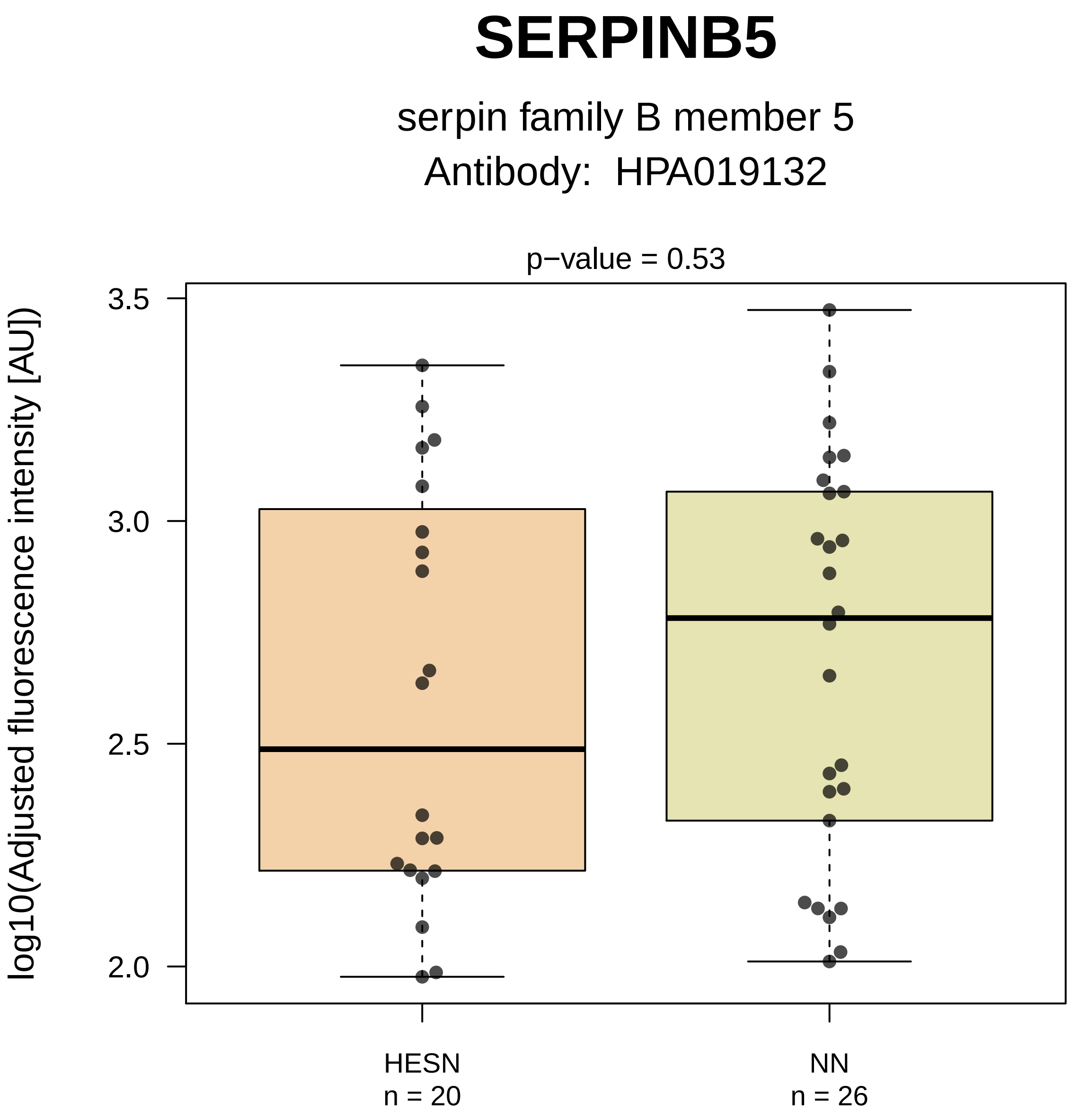

SERPINB5
serpin family B member 5
Antibody: HPA019132
p−value = 0.53
log10(Adjusted fluorescence intensity [AU])
3.5
3.0
2.5
2.0
HESN
n = 20
NN
n = 26

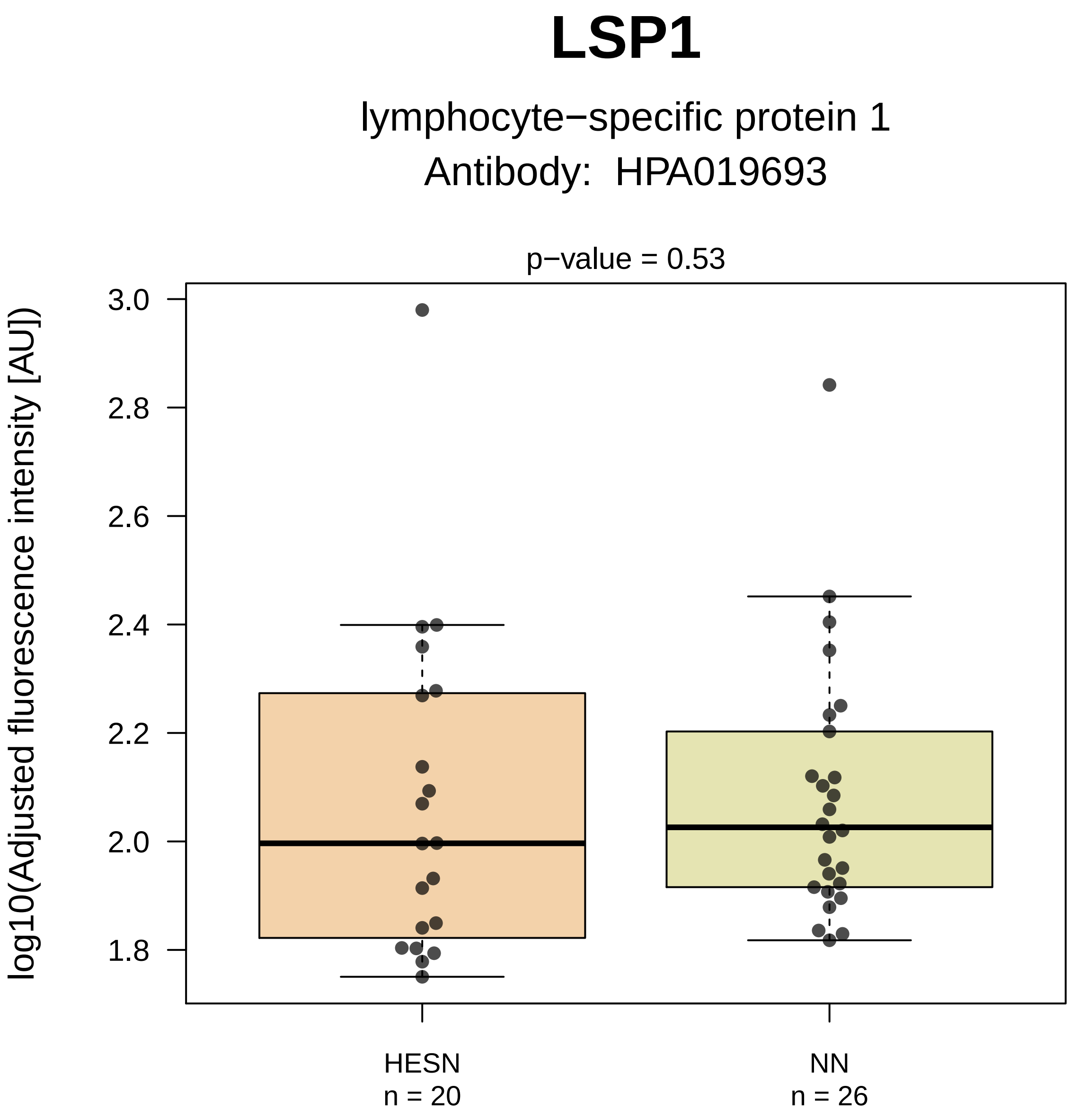
LSP1
lymphocyte−specific protein 1
Antibody: HPA019693
p−value = 0.53
log10(Adjusted fluorescence intensity [AU])
3.0
2.8
2.6
2.4
2.2
2.0
1.8
HESN
n = 20
NN
n = 26

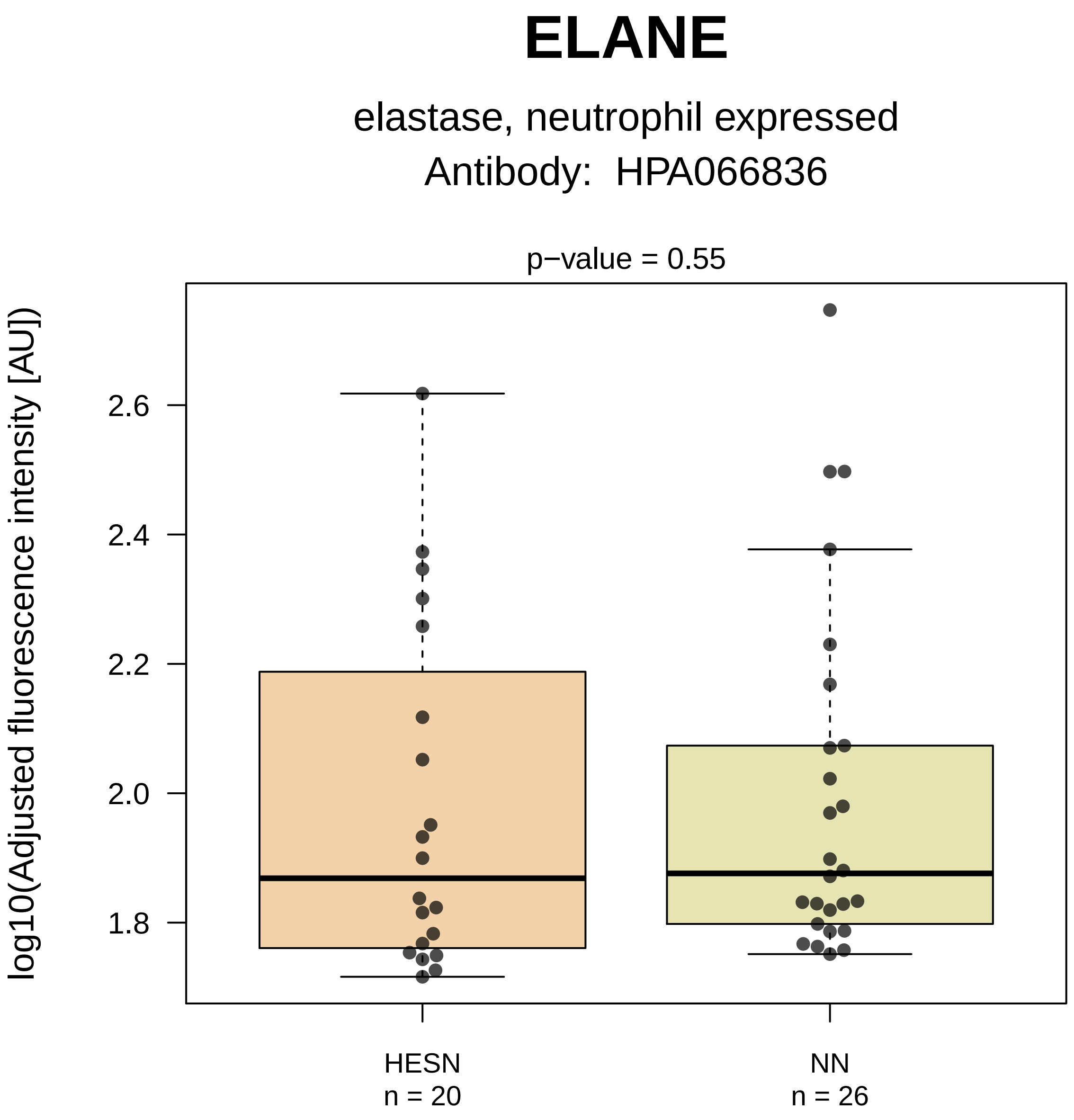

ELANE
elastase, neutrophil expressed
Antibody: HPA066836
p−value = 0.55
log10(Adjusted fluorescence intensity [AU])
2.6
2.4
2.2
2.0
1.8
HESN
n = 20
NN
n = 26

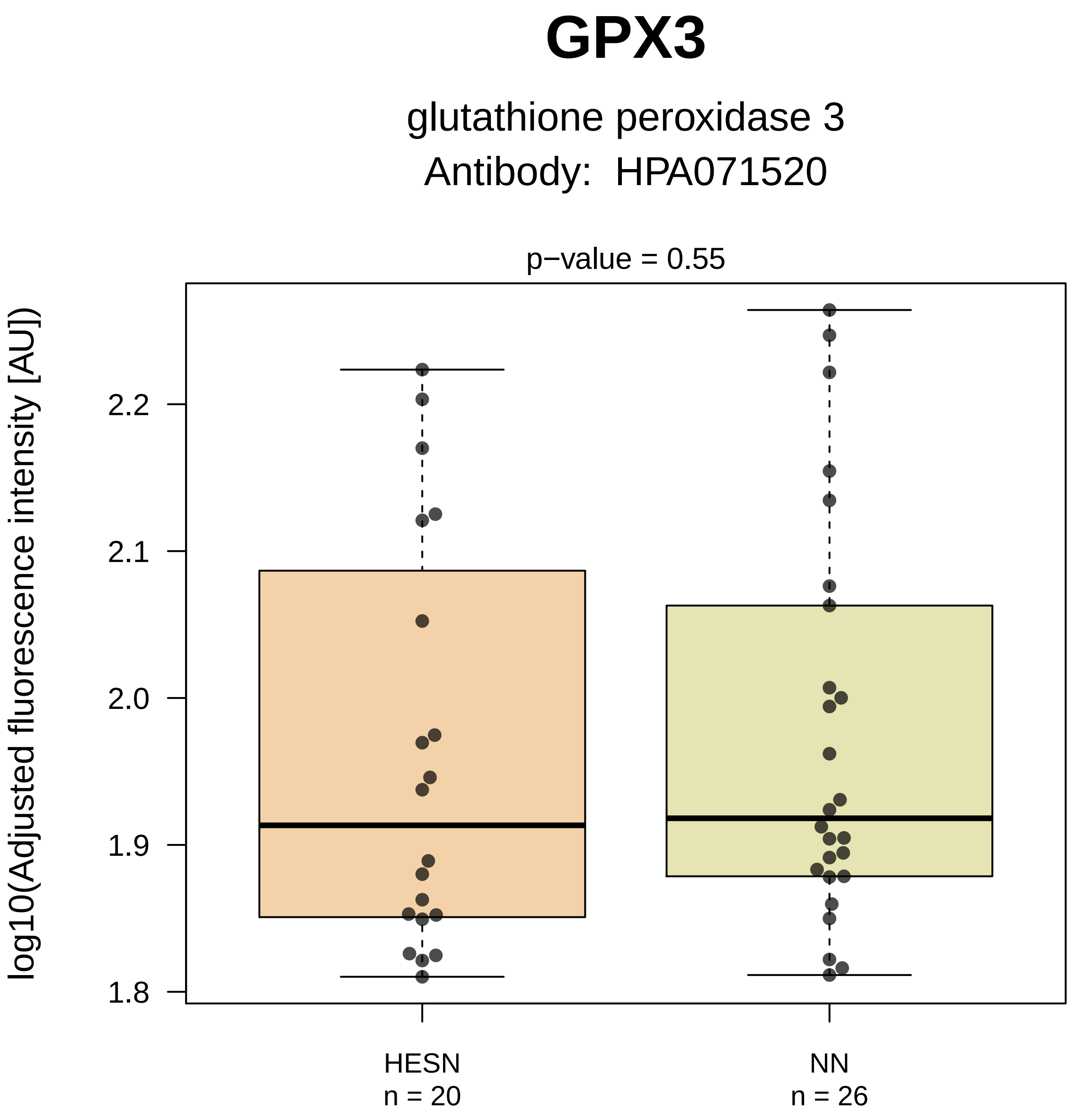
GPX3
glutathione peroxidase 3
Antibody: HPA071520
p−value = 0.55
log10(Adjusted fluorescence intensity [AU])
2.2
2.1
2.0
1.9
1.8
HESN
n = 20
NN
n = 26

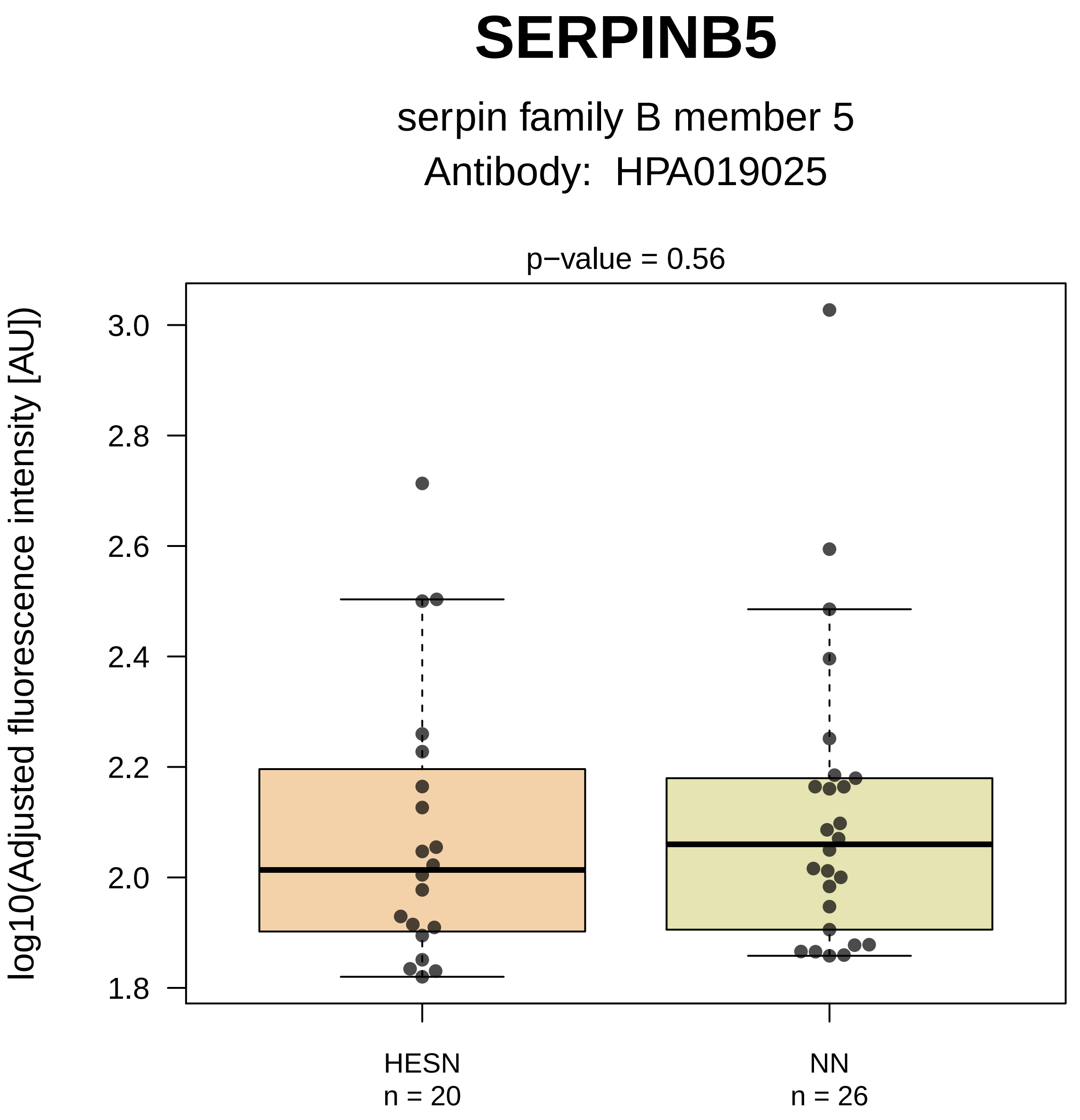

SERPINB5
serpin family B member 5
Antibody: HPA019025
p−value = 0.56
log10(Adjusted fluorescence intensity [AU])
3.0
2.8
2.6
2.4
2.2
2.0
1.8
HESN
n = 20
NN
n = 26

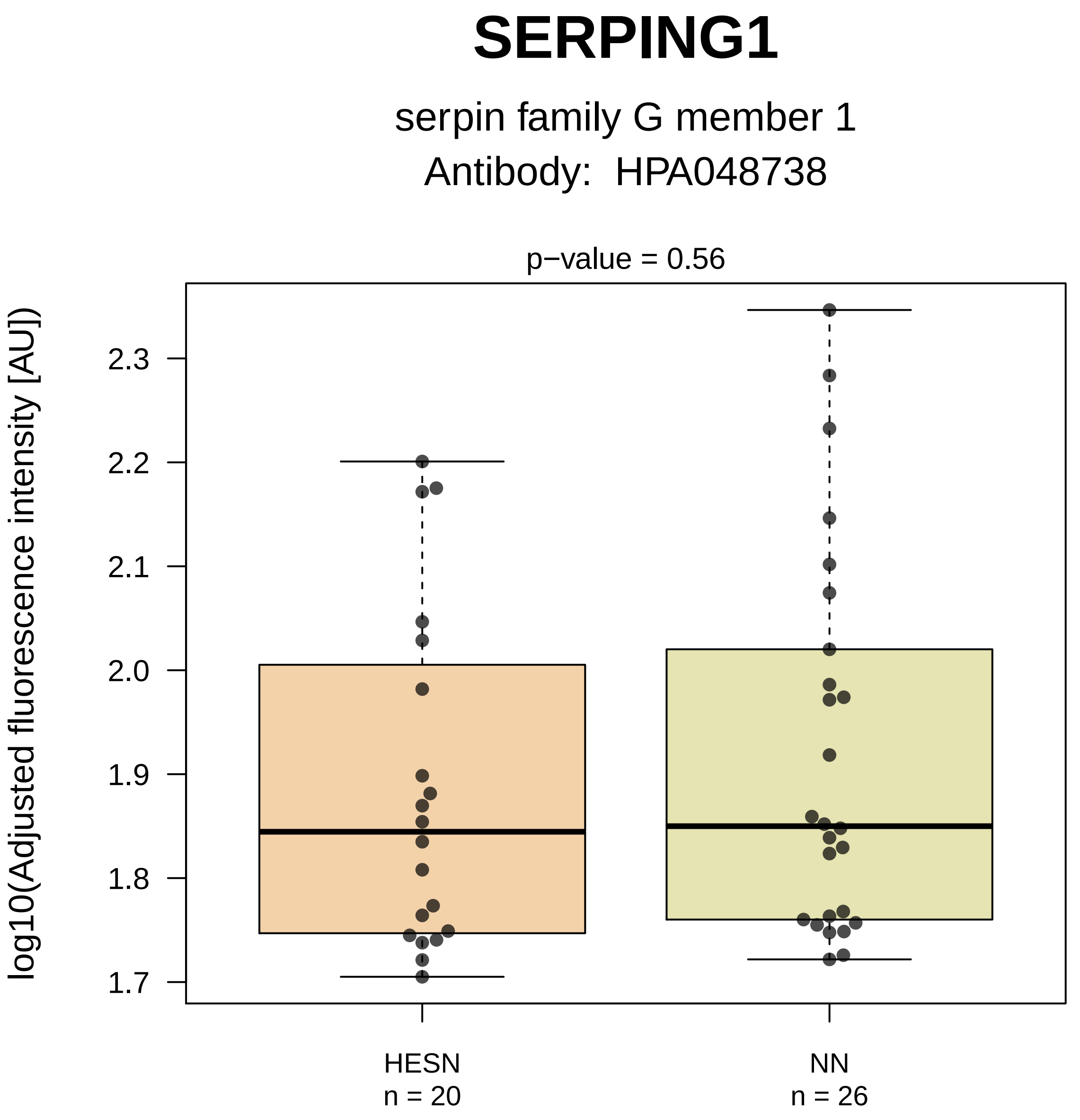
SERPING1
serpin family G member 1
Antibody: HPA048738
p−value = 0.56
log10(Adjusted fluorescence intensity [AU])
2.3
2.2
2.1
2.0
1.9
1.8
1.7
HESN
n = 20
NN
n = 26

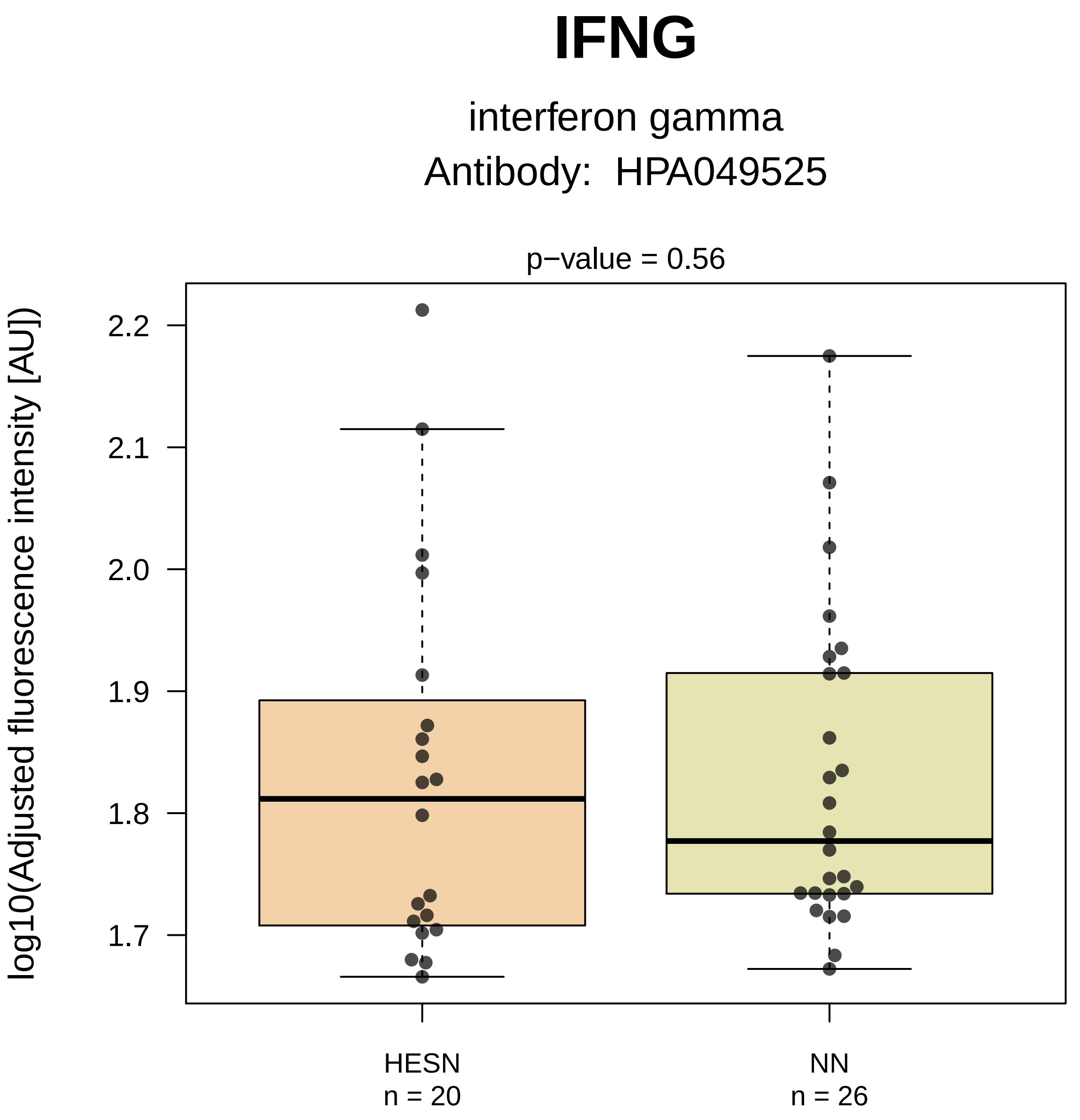

IFNG
interferon gamma
Antibody: HPA049525
p−value = 0.56
log10(Adjusted fluorescence intensity [AU])
2.2
2.1
2.0
1.9
1.8
1.7
HESN
n = 20
NN
n = 26

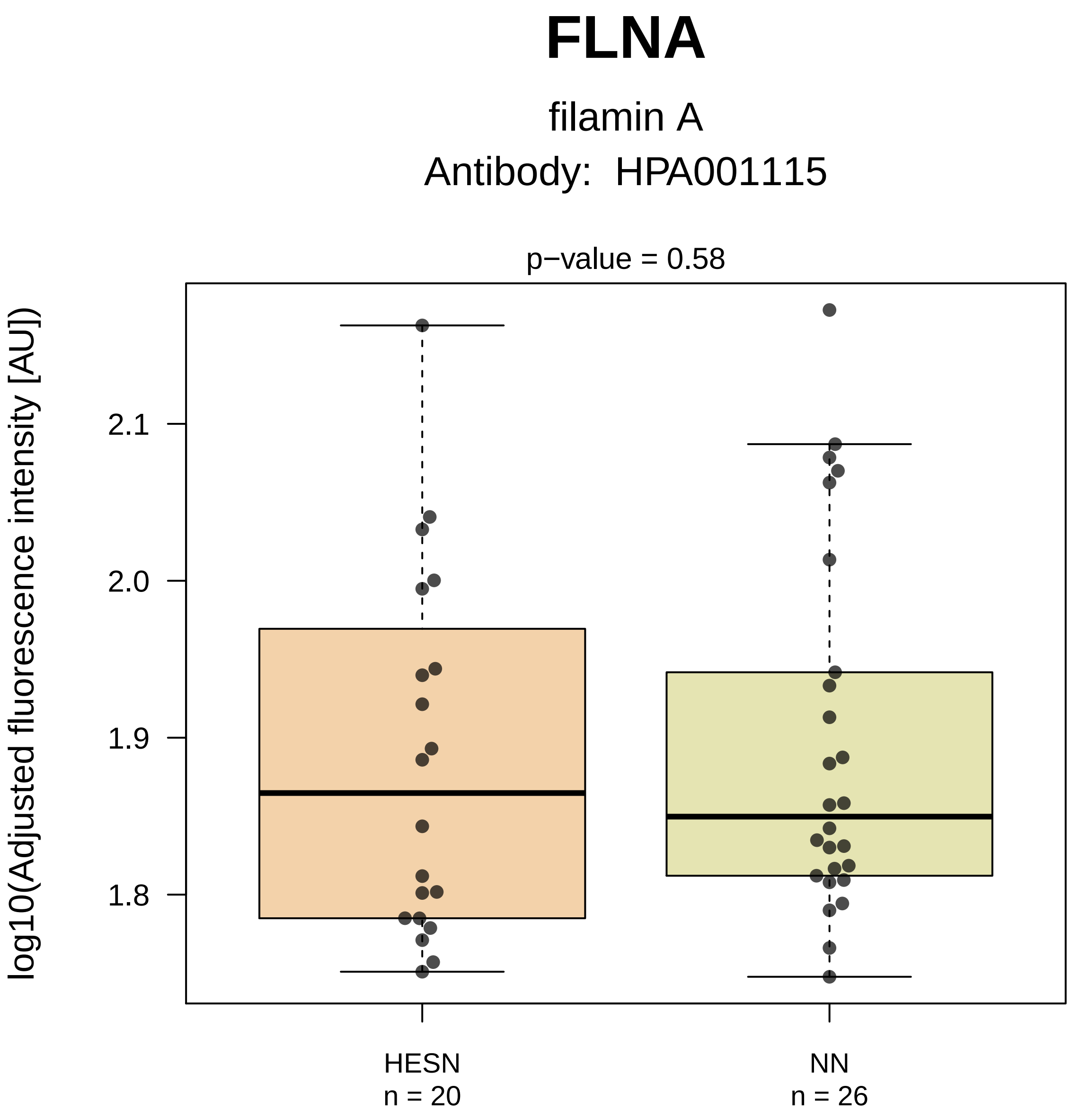
FLNA
filamin A
Antibody: HPA001115
p−value = 0.58
log10(Adjusted fluorescence intensity [AU])
2.1
2.0
1.9
1.8
HESN
n = 20
NN
n = 26

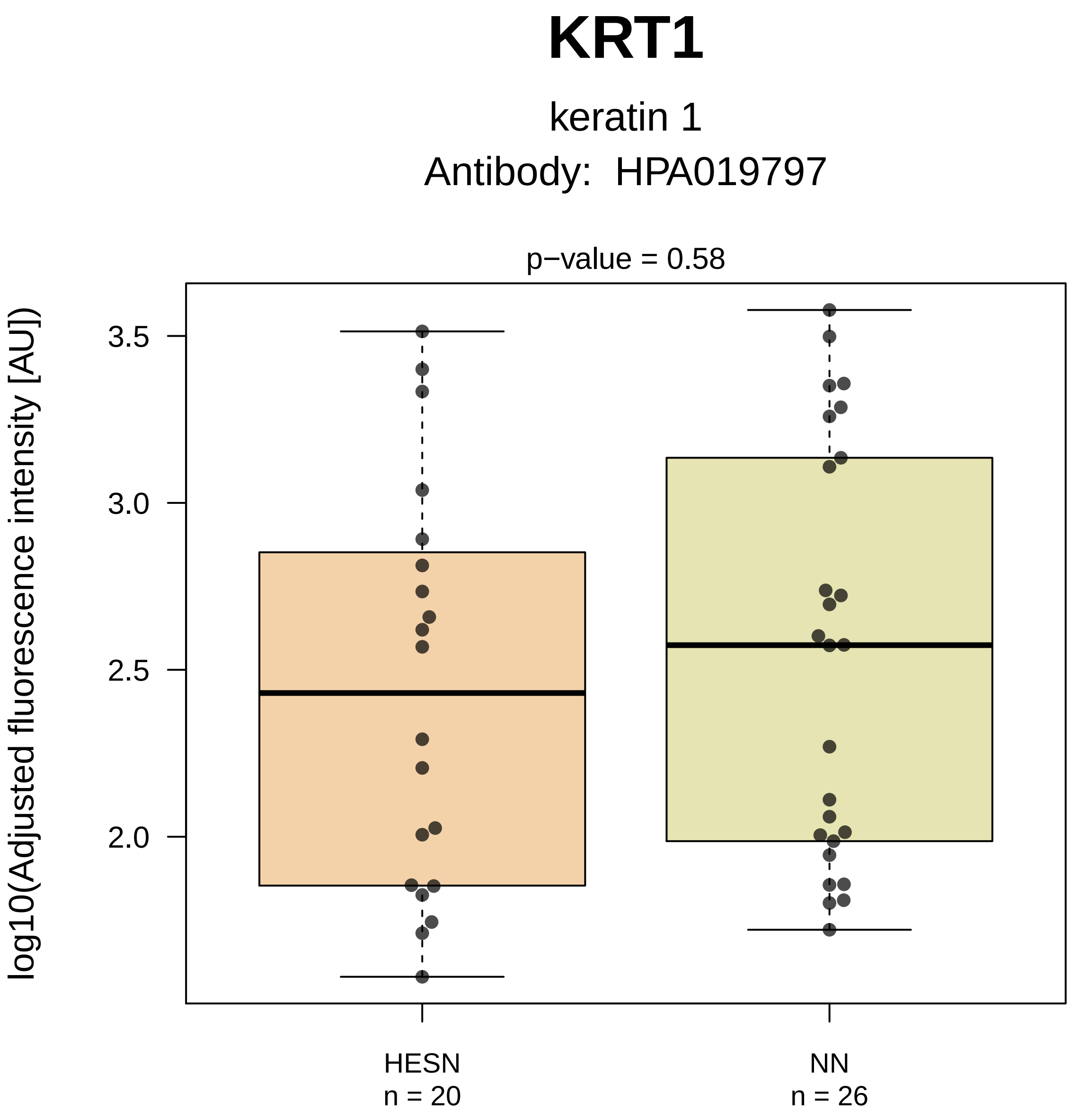
KRT1
keratin 1
Antibody: HPA019797
p−value = 0.58
log10(Adjusted fluorescence intensity [AU])
3.5
3.0
2.5
2.0
HESN
n = 20
NN
n = 26

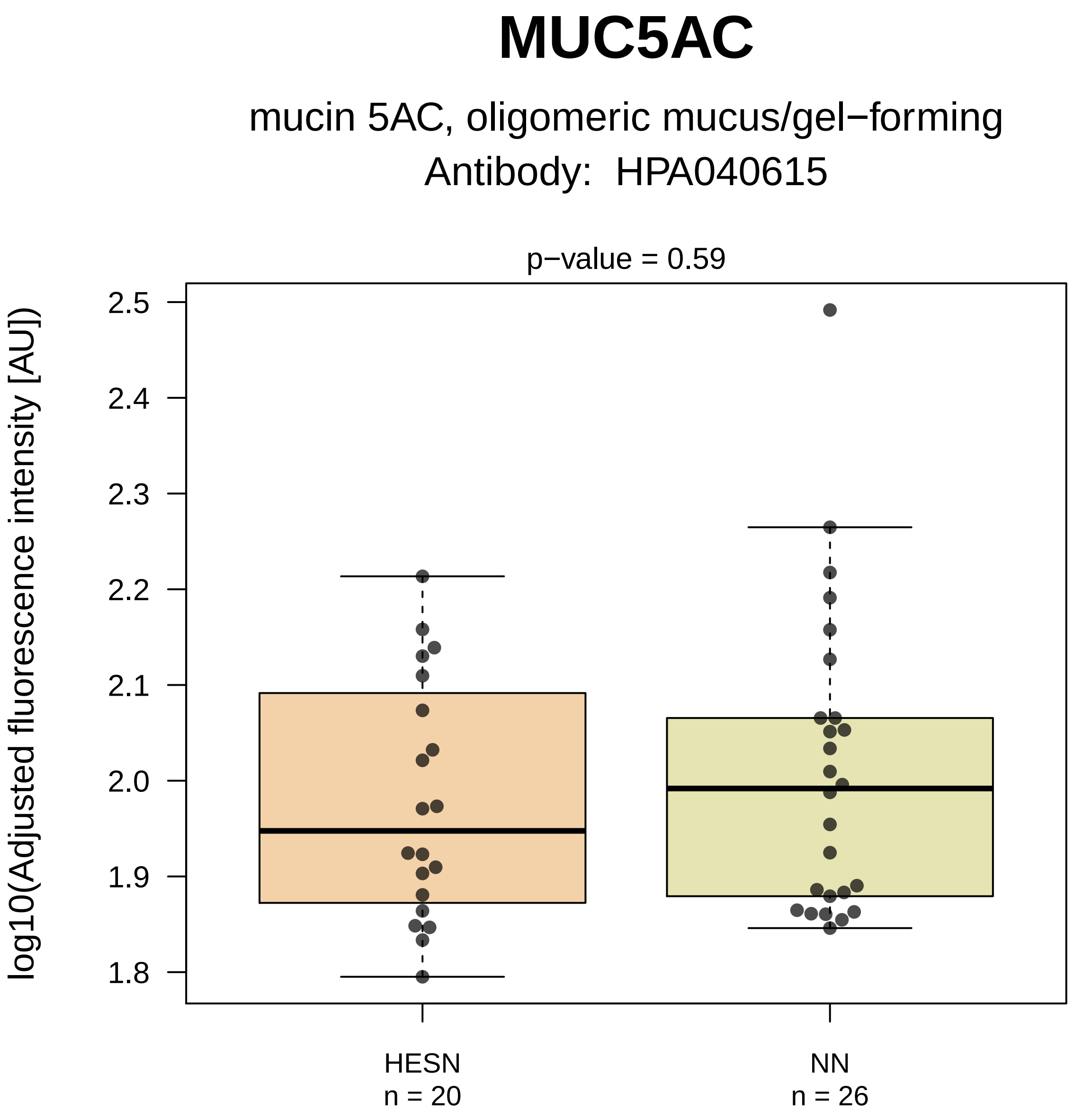
MUC5AC
mucin 5AC, oligomeric mucus/gel−forming
Antibody: HPA040615
p−value = 0.59
log10(Adjusted fluorescence intensity [AU])
2.5
2.4
2.3
2.2
2.1
2.0
1.9
1.8
HESN
n = 20
NN
n = 26

TACSTD2

tumor associated calcium signal transducer 2
Antibody: HPA043104

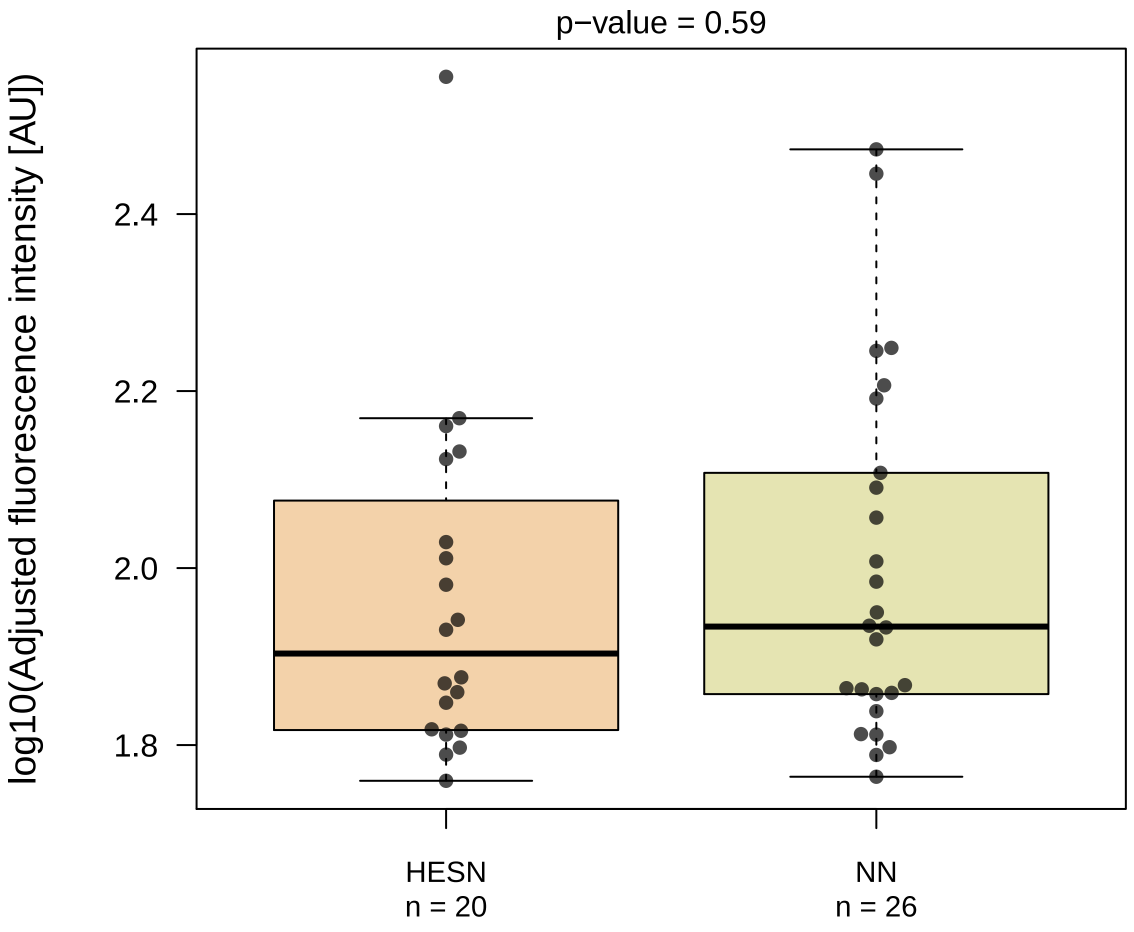

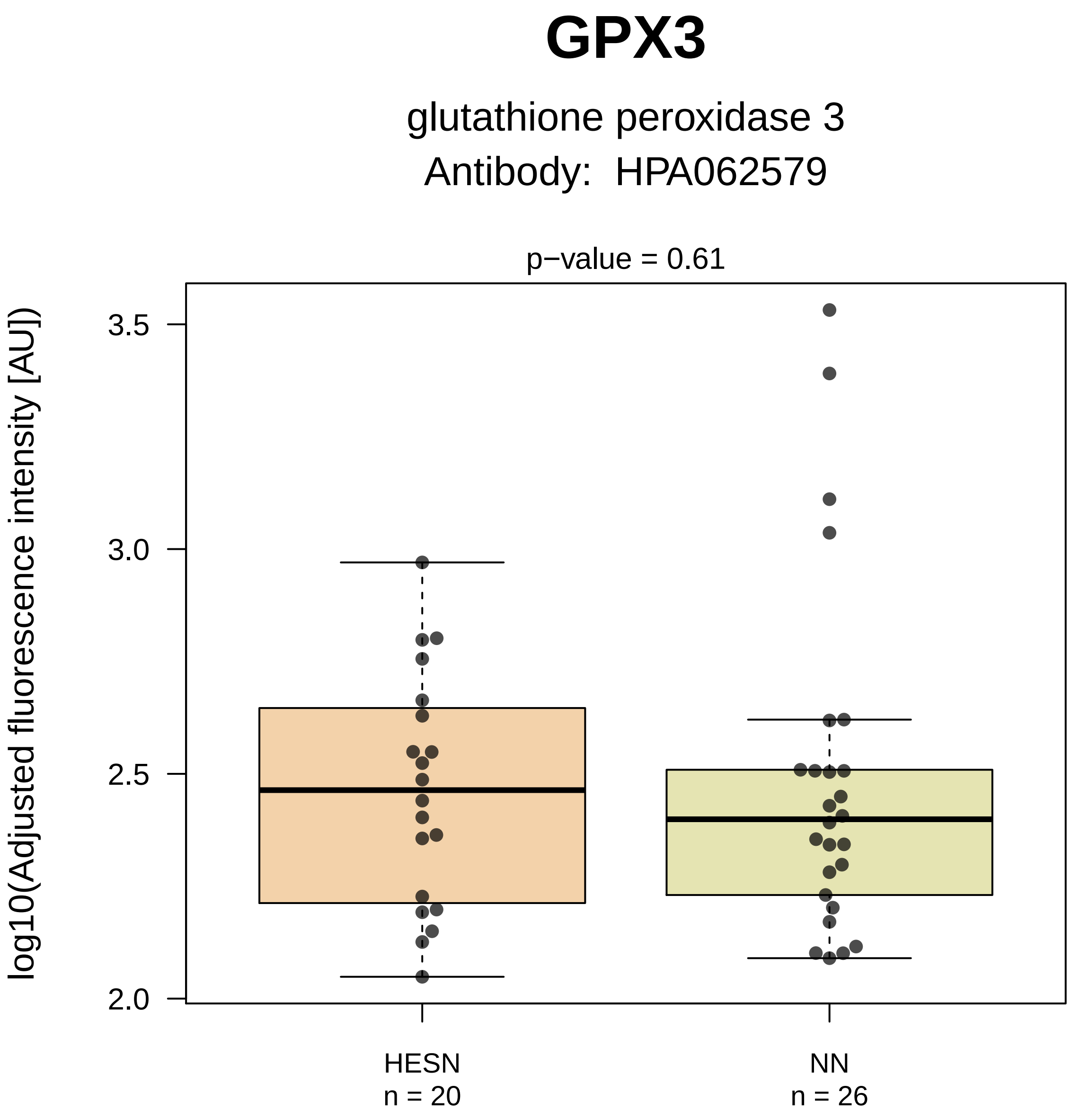
GPX3
glutathione peroxidase 3
Antibody: HPA062579
p−value = 0.61
log10(Adjusted fluorescence intensity [AU])
3.5
3.0
2.5
2.0
HESN
n = 20
NN
n = 26

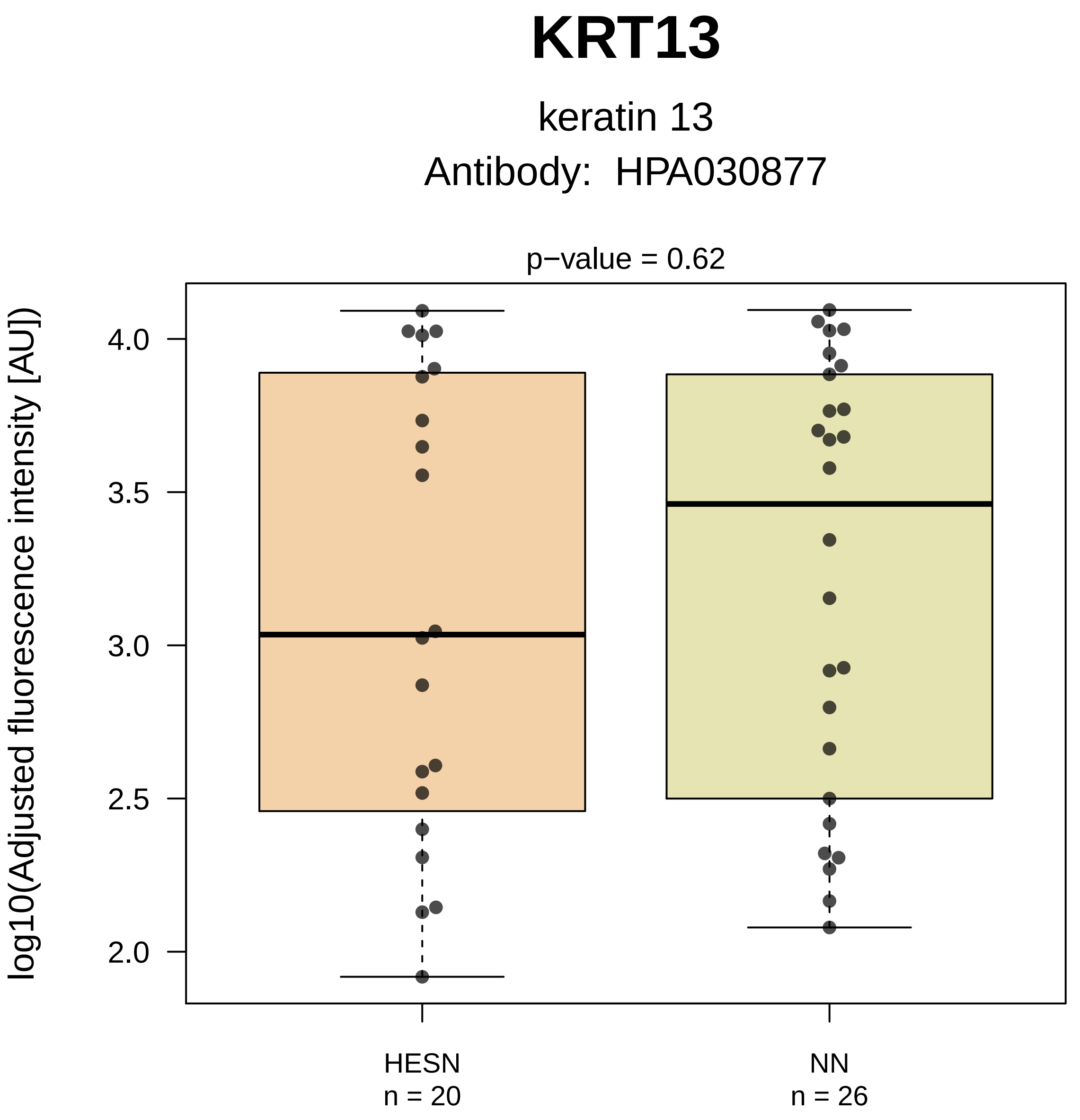

KRT13
keratin 13
Antibody: HPA030877
p−value = 0.62
log10(Adjusted fluorescence intensity [AU])
4.0
3.5
3.0
2.5
2.0
HESN
n = 20
NN
n = 26

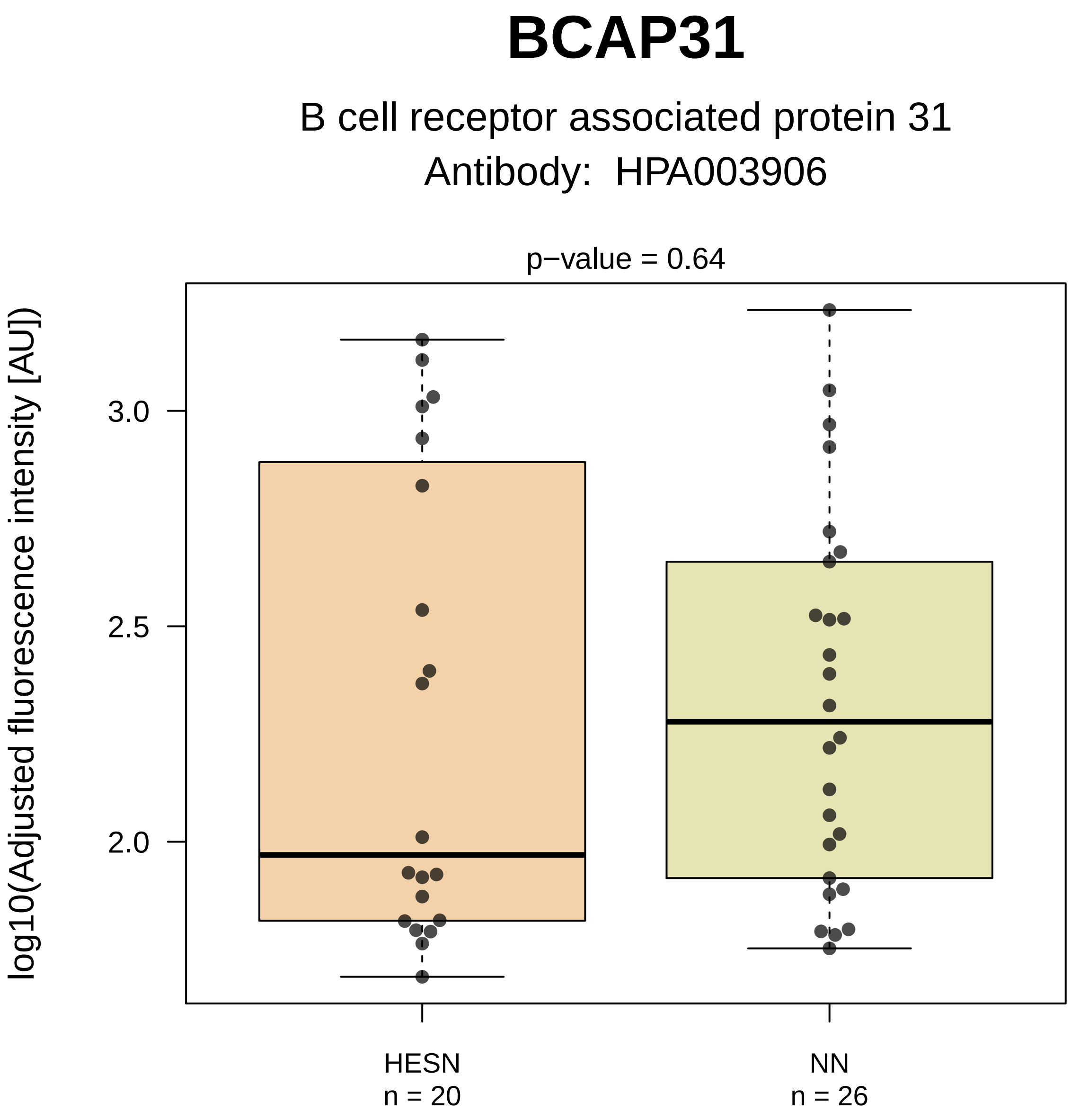

BCAP31
B cell receptor associated protein 31
Antibody: HPA003906
p−value = 0.64
log10(Adjusted fluorescence intensity [AU])
3.0
2.5
2.0
HESN
n = 20
NN
n = 26

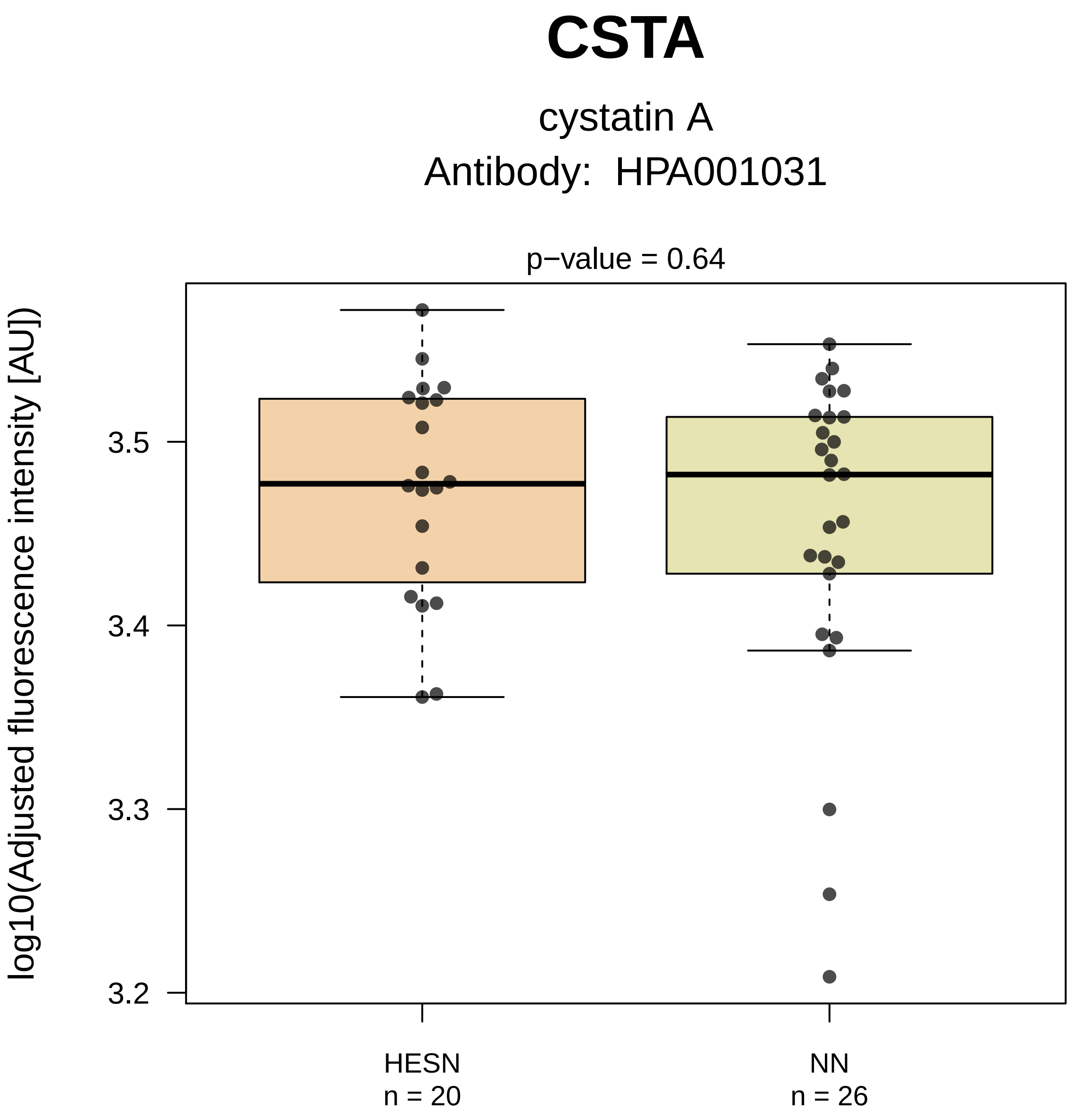

CSTA
cystatin A
Antibody: HPA001031
p−value = 0.64
log10(Adjusted fluorescence intensity [AU])
3.2
3.3
3.4
3.5
HESN
n = 20
NN
n = 26

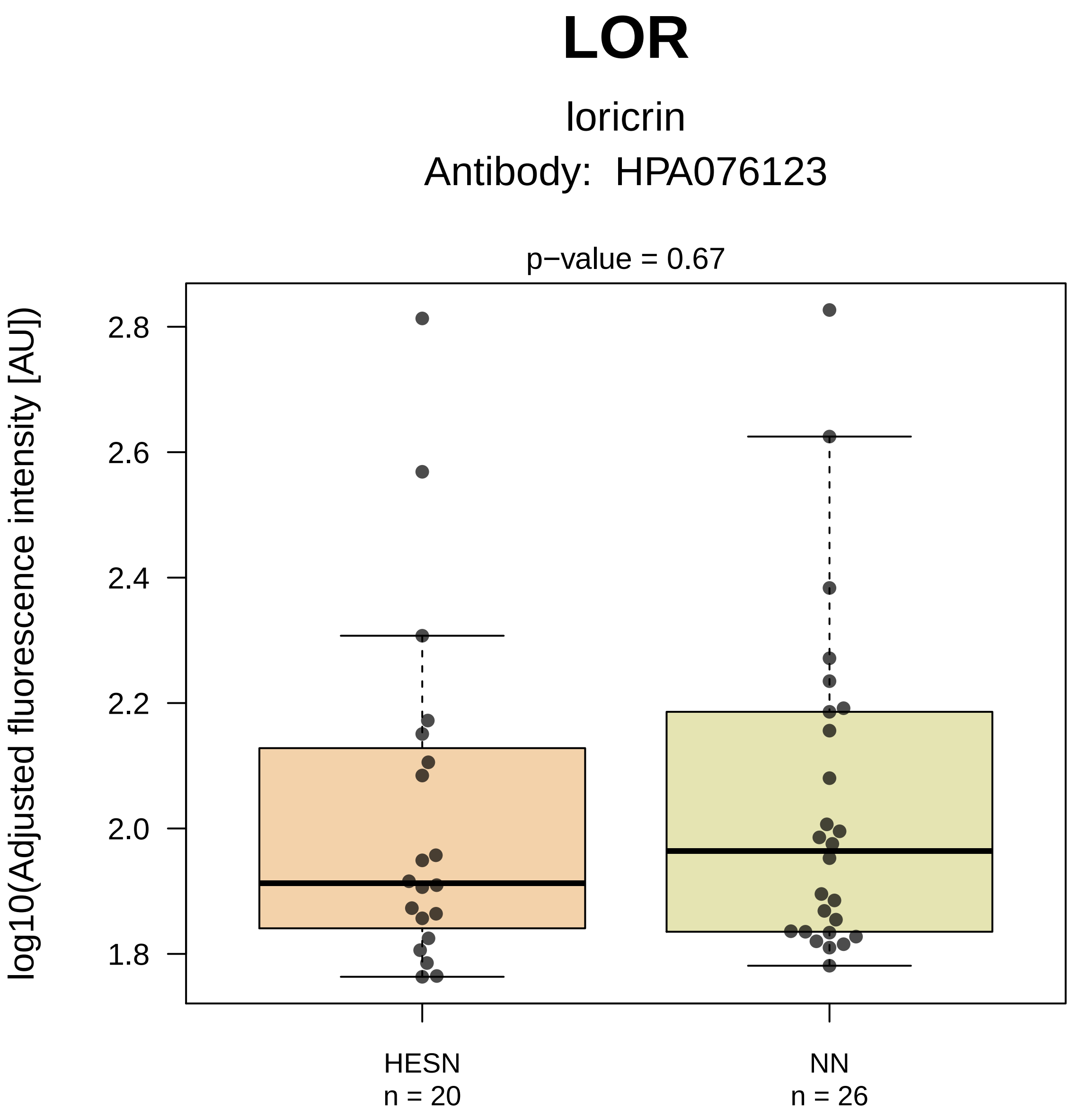

LOR
loricrin
Antibody: HPA076123
p−value = 0.67
log10(Adjusted fluorescence intensity [AU])
2.8
2.6
2.4
2.2
2.0
1.8
HESN
n = 20
NN
n = 26

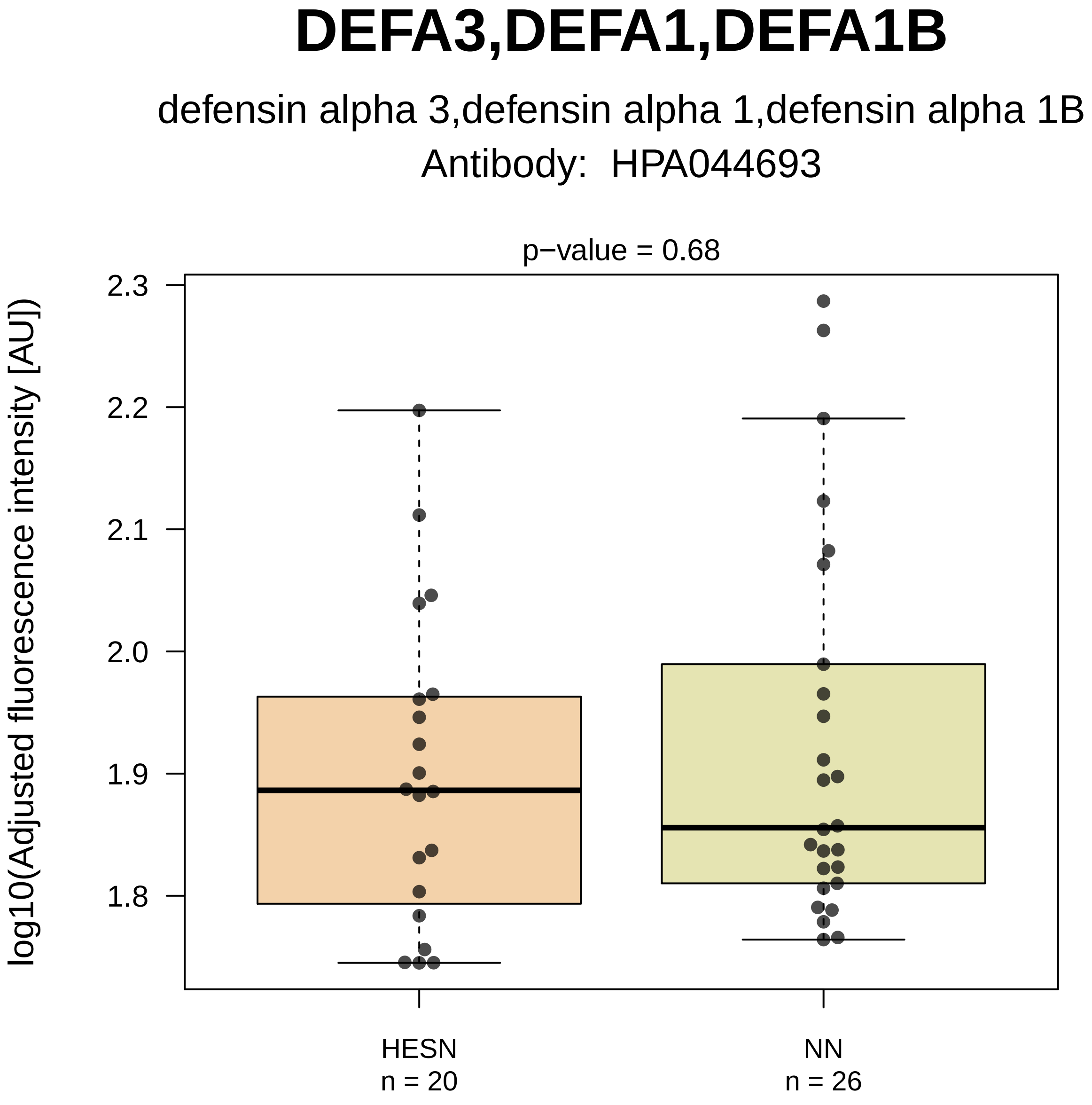

DEFA3,DEFA1,DEFA1B
defensin alpha 3,defensin alpha 1,defensin alpha 1B
Antibody: HPA044693
p−value = 0.68
log10(Adjusted fluorescence intensity [AU])
2.3
2.2
2.1
2.0
1.9
1.8
HESN
n = 20
NN
n = 26

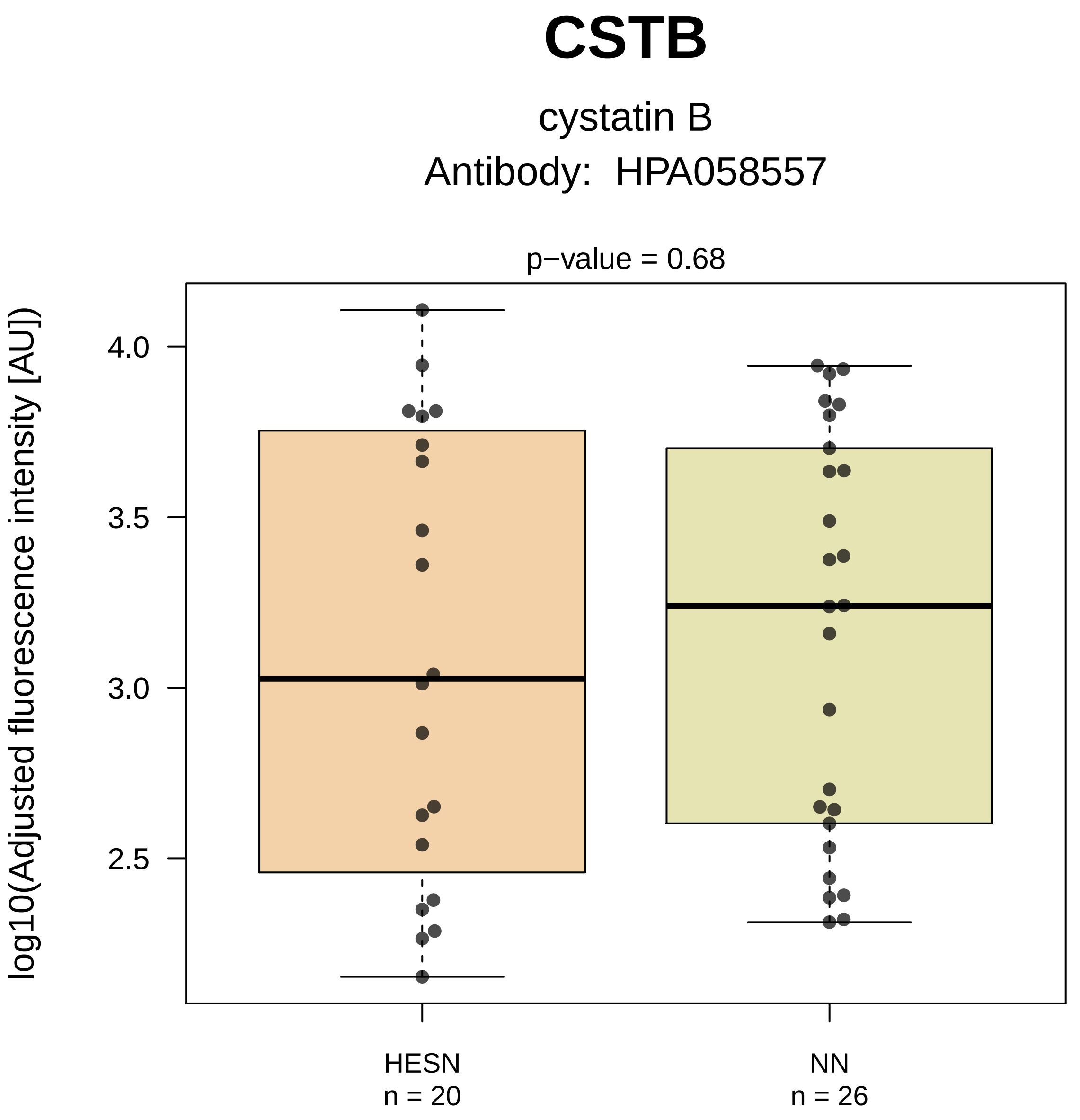
CSTB
cystatin B
Antibody: HPA058557
p−value = 0.68
log10(Adjusted fluorescence intensity [AU])
4.0
3.5
3.0
2.5
HESN
n = 20
NN
n = 26

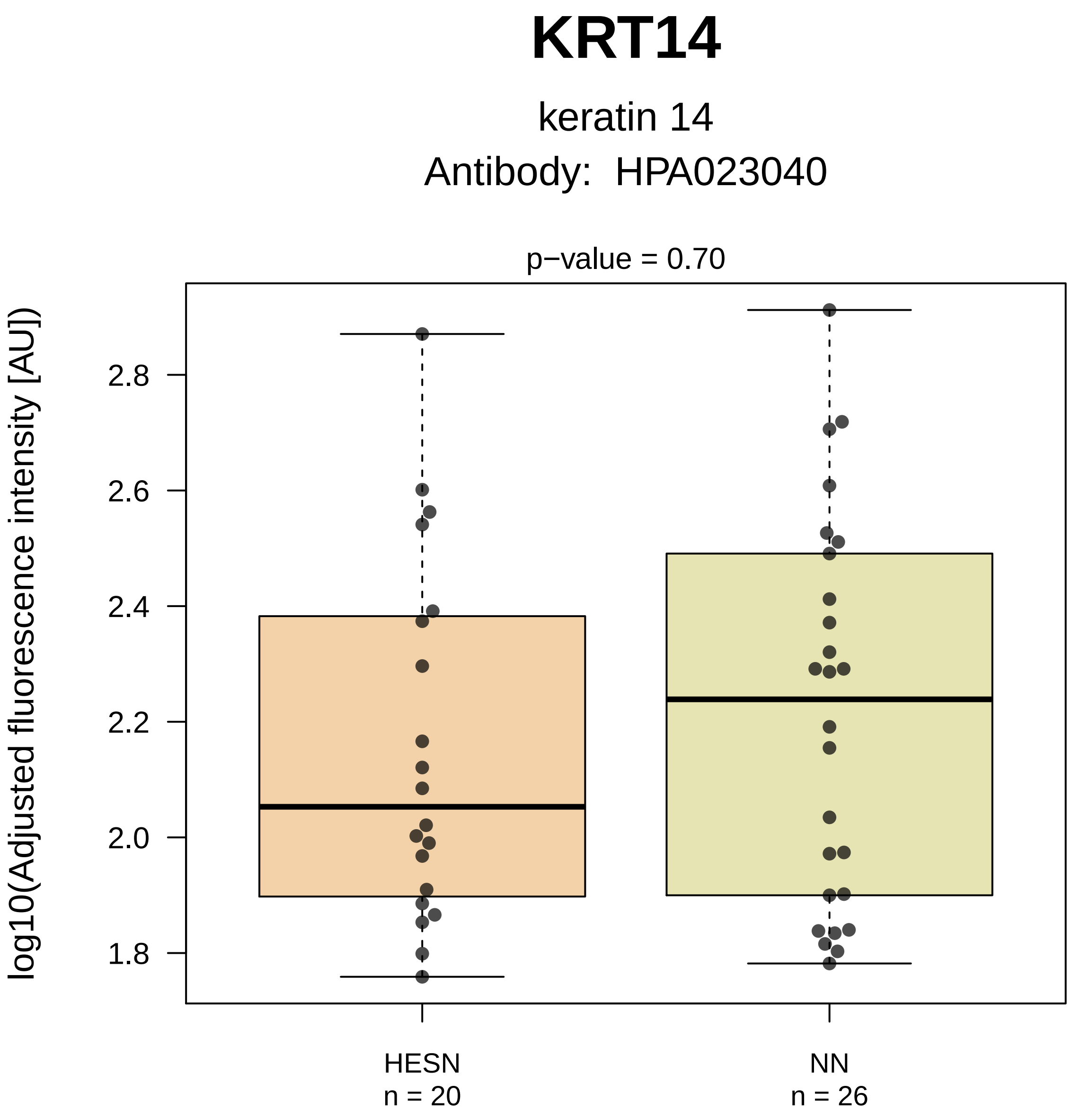

KRT14
keratin 14
Antibody: HPA023040
p−value = 0.70
log10(Adjusted fluorescence intensity [AU])
2.8
2.6
2.4
2.2
2.0
1.8
HESN
n = 20
NN
n = 26

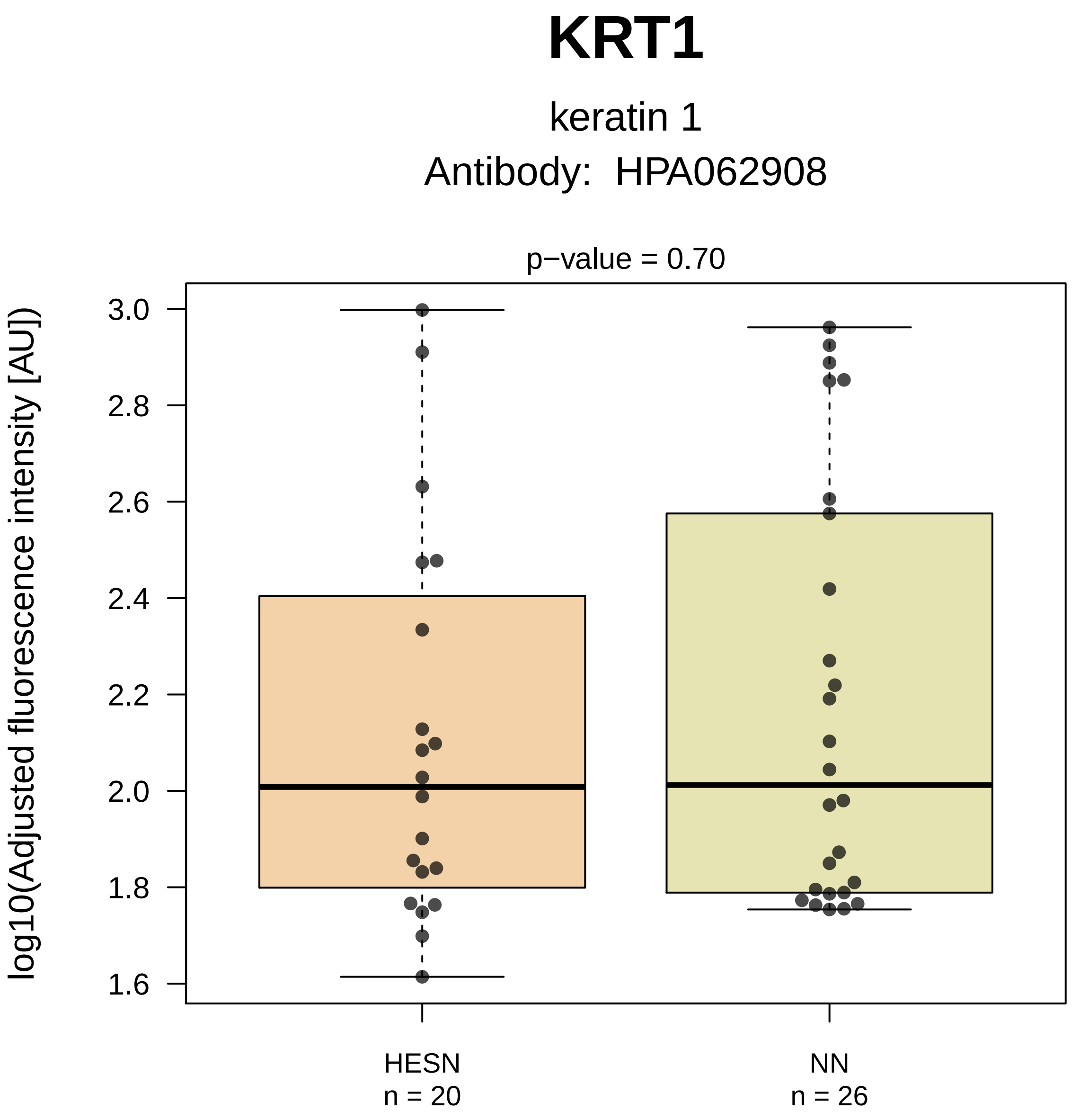
KRT1
keratin 1
Antibody: HPA062908
p−value = 0.70
log10(Adjusted fluorescence intensity [AU])
3.0
2.8
2.6
2.4
2.2
2.0
1.8
1.6
HESN
n = 20
NN
n = 26

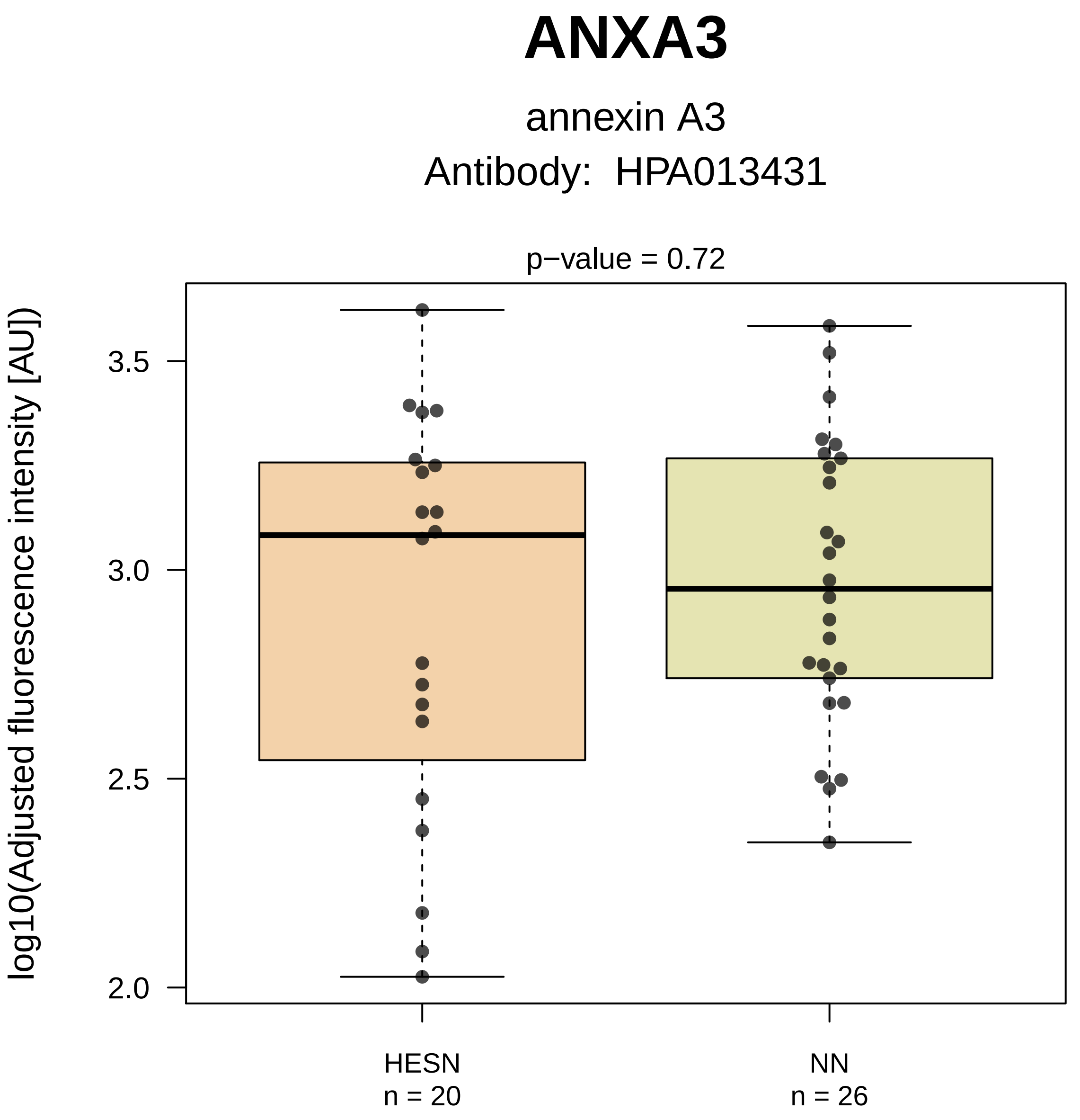
ANXA3
annexin A3
Antibody: HPA013431
p−value = 0.72
log10(Adjusted fluorescence intensity [AU])
3.5
3.0
2.5
2.0
HESN
n = 20
NN
n = 26

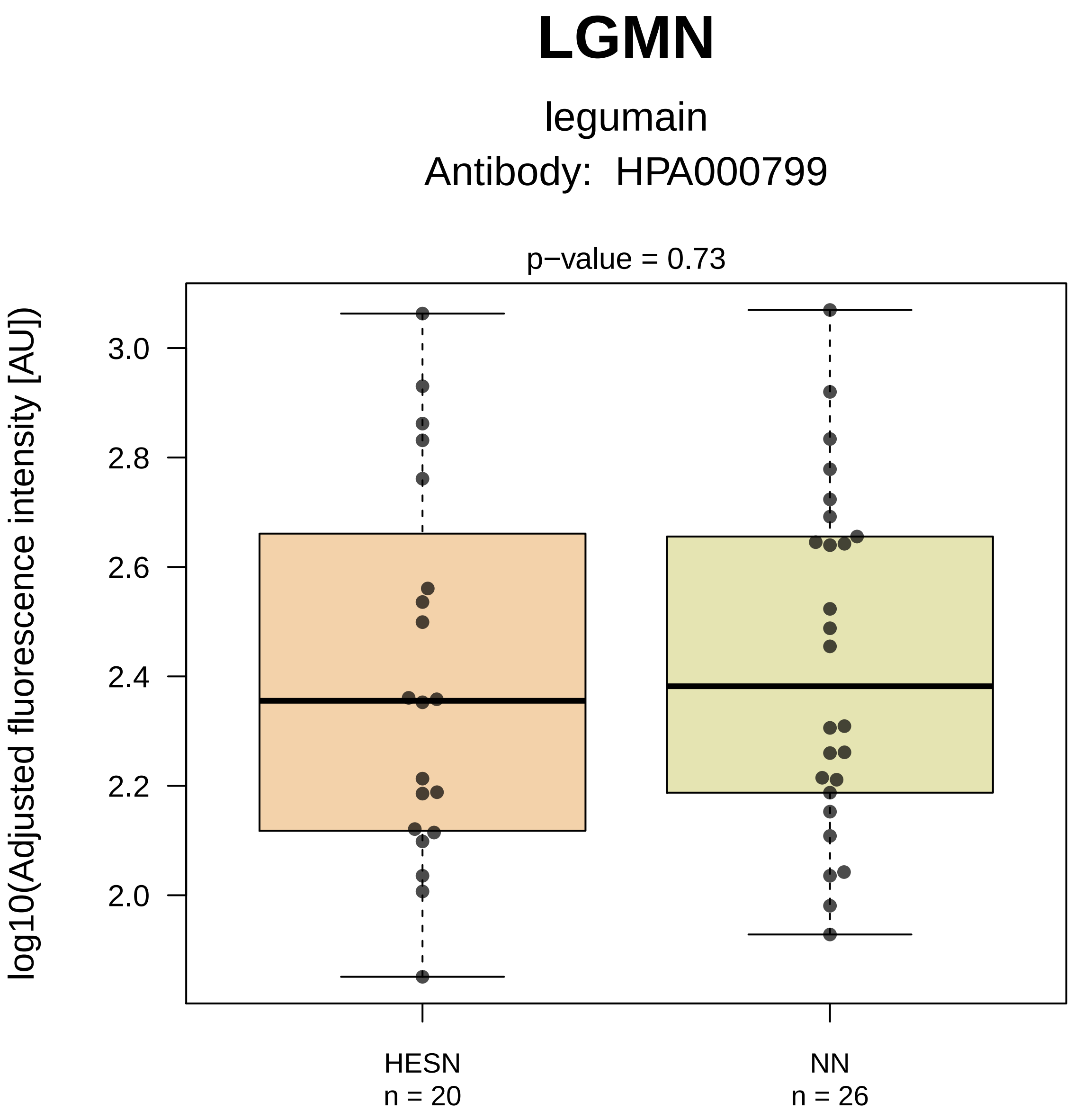
LGMN
legumain
Antibody: HPA000799
p−value = 0.73
log10(Adjusted fluorescence intensity [AU])
3.0
2.8
2.6
2.4
2.2
2.0
HESN
n = 20
NN
n = 26

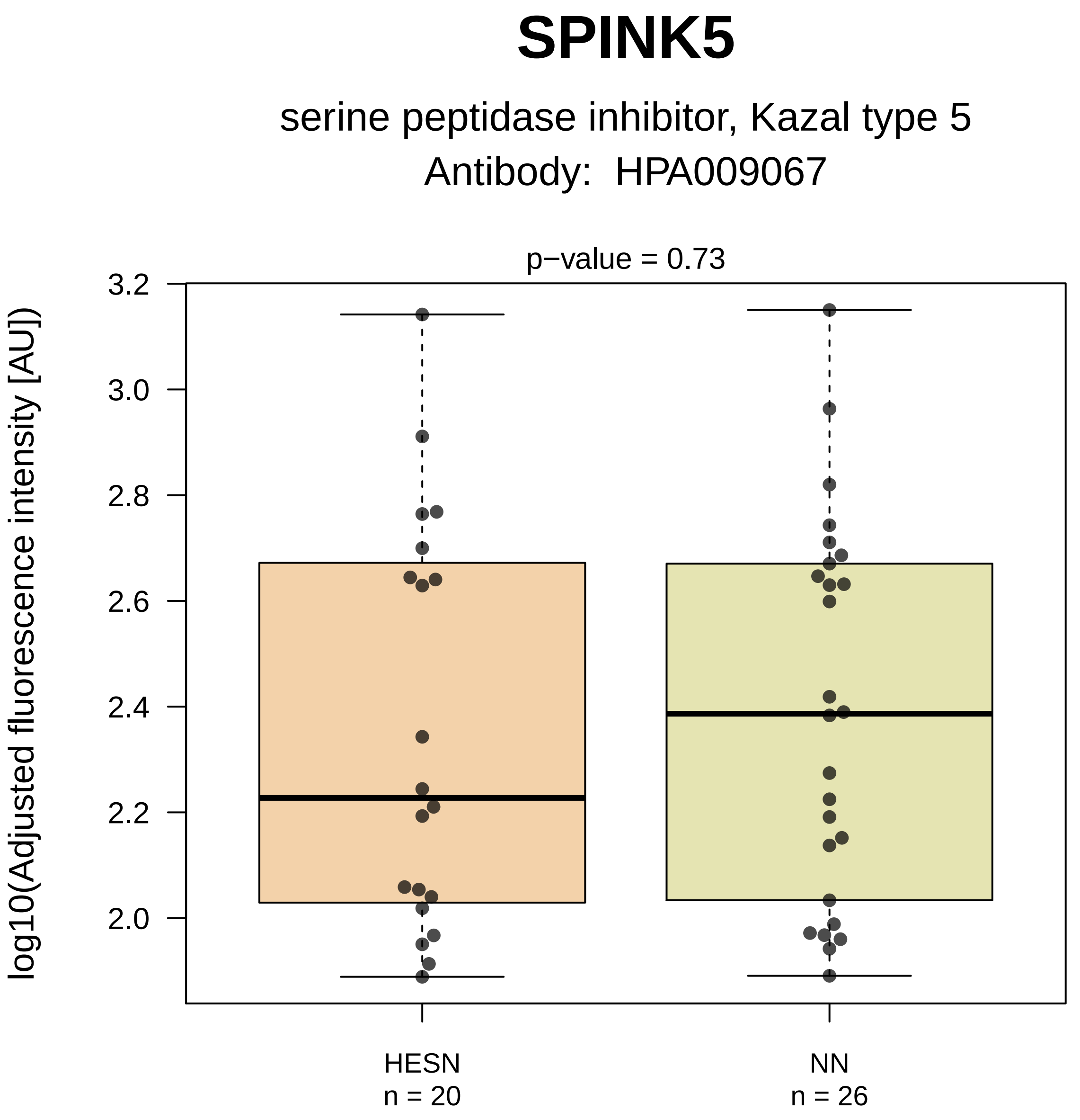
SPINK5
serine peptidase inhibitor, Kazal type 5
Antibody: HPA009067
p−value = 0.73
log10(Adjusted fluorescence intensity [AU])
3.2
3.0
2.8
2.6
2.4
2.2
2.0
HESN
n = 20
NN
n = 26

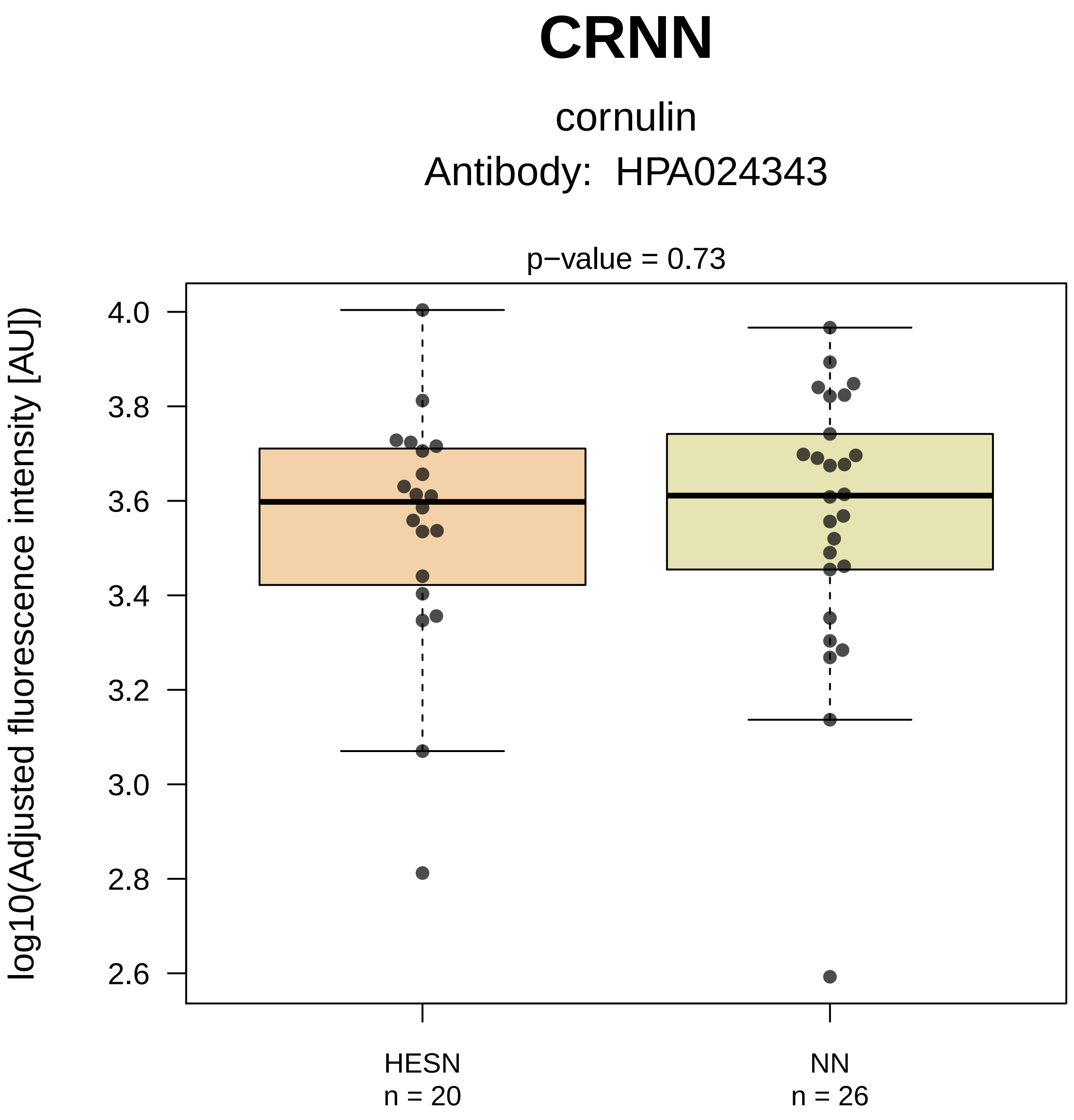
CRNN
cornulin
Antibody: HPA024343
p−value = 0.73
log10(Adjusted fluorescence intensity [AU])
4.0
3.8
3.6
3.4
3.2
3.0
2.8
2.6
HESN
n = 20
NN
n = 26

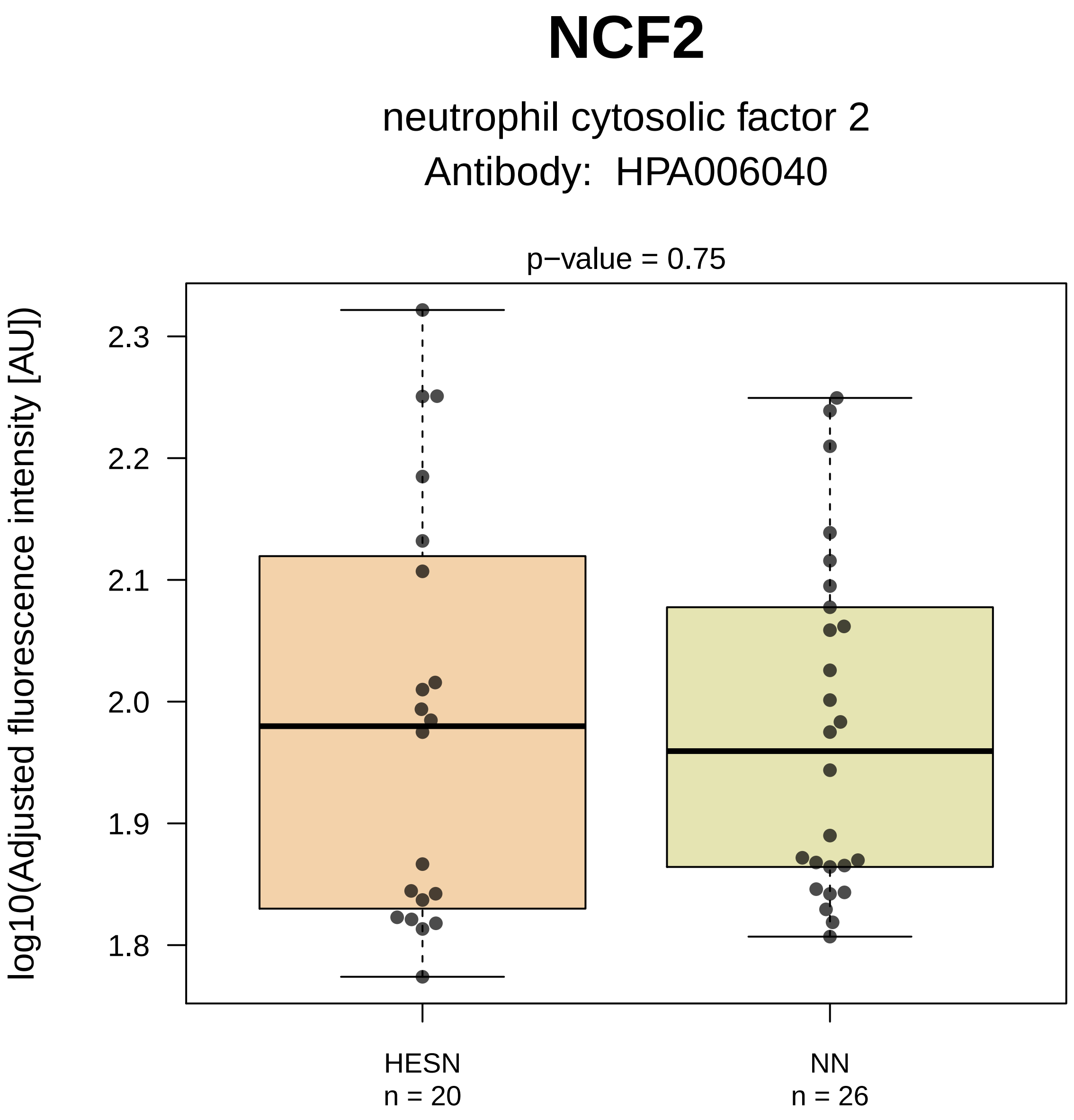
NCF2
neutrophil cytosolic factor 2
Antibody: HPA006040
p−value = 0.75
log10(Adjusted fluorescence intensity [AU])
2.3
2.2
2.1
2.0
1.9
1.8
HESN
n = 20
NN
n = 26

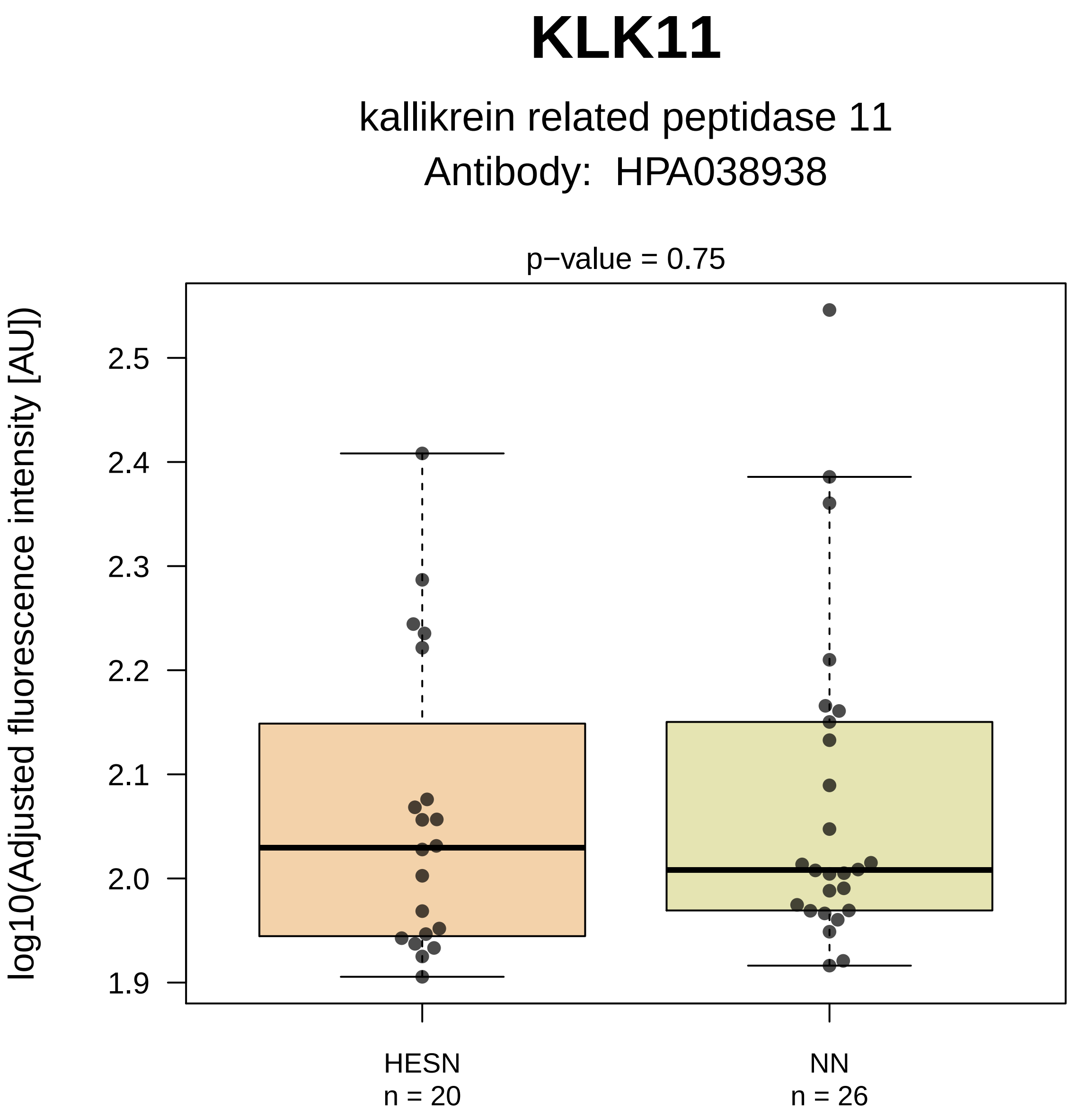
KLK11
kallikrein related peptidase 11
Antibody: HPA038938
p−value = 0.75
log10(Adjusted fluorescence intensity [AU])
2.5
2.4
2.3
2.2
2.1
2.0
1.9
HESN
n = 20
NN
n = 26

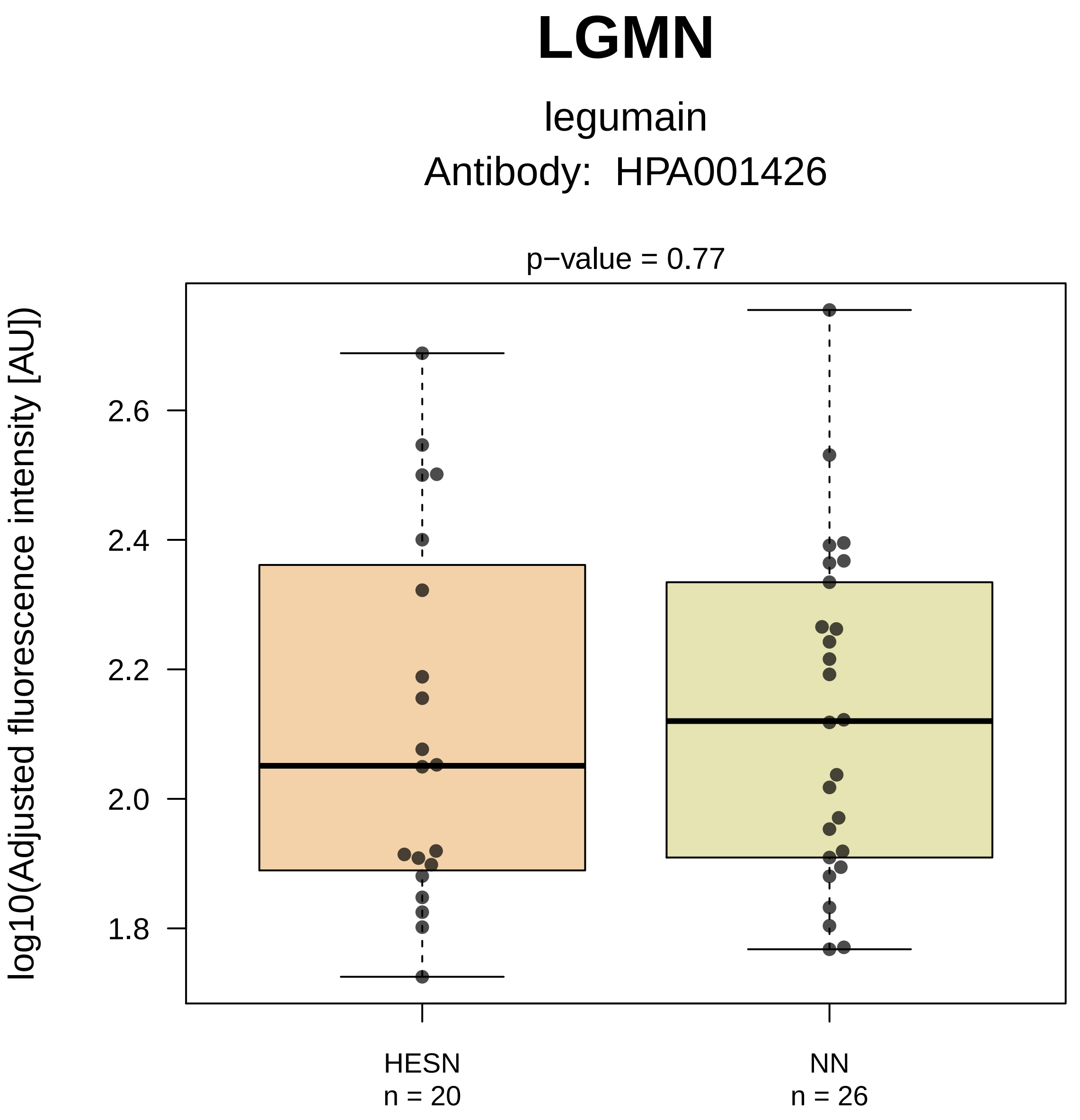
LGMN
legumain
Antibody: HPA001426
p−value = 0.77
log10(Adjusted fluorescence intensity [AU])
2.6
2.4
2.2
2.0
1.8
HESN
n = 20
NN
n = 26

KRT1

keratin 1
Antibody: HPA017917

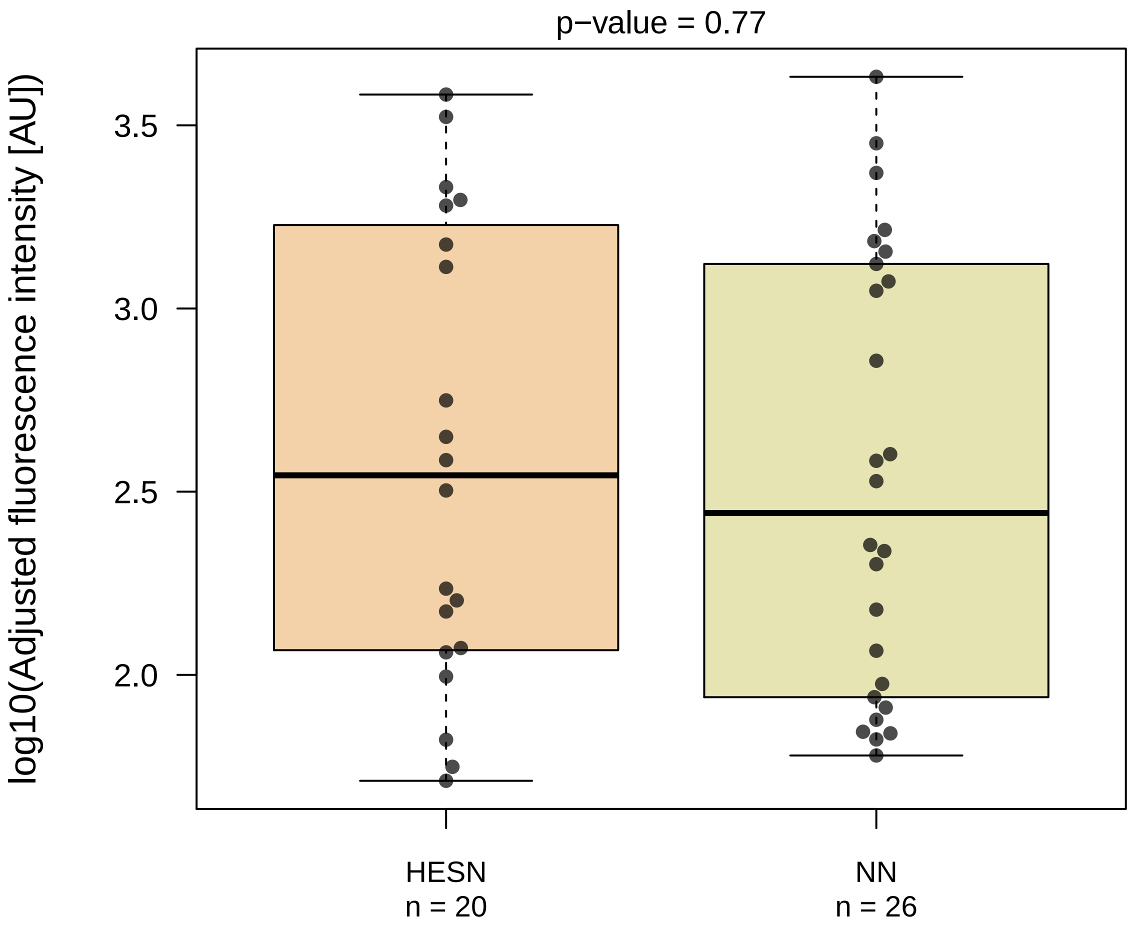

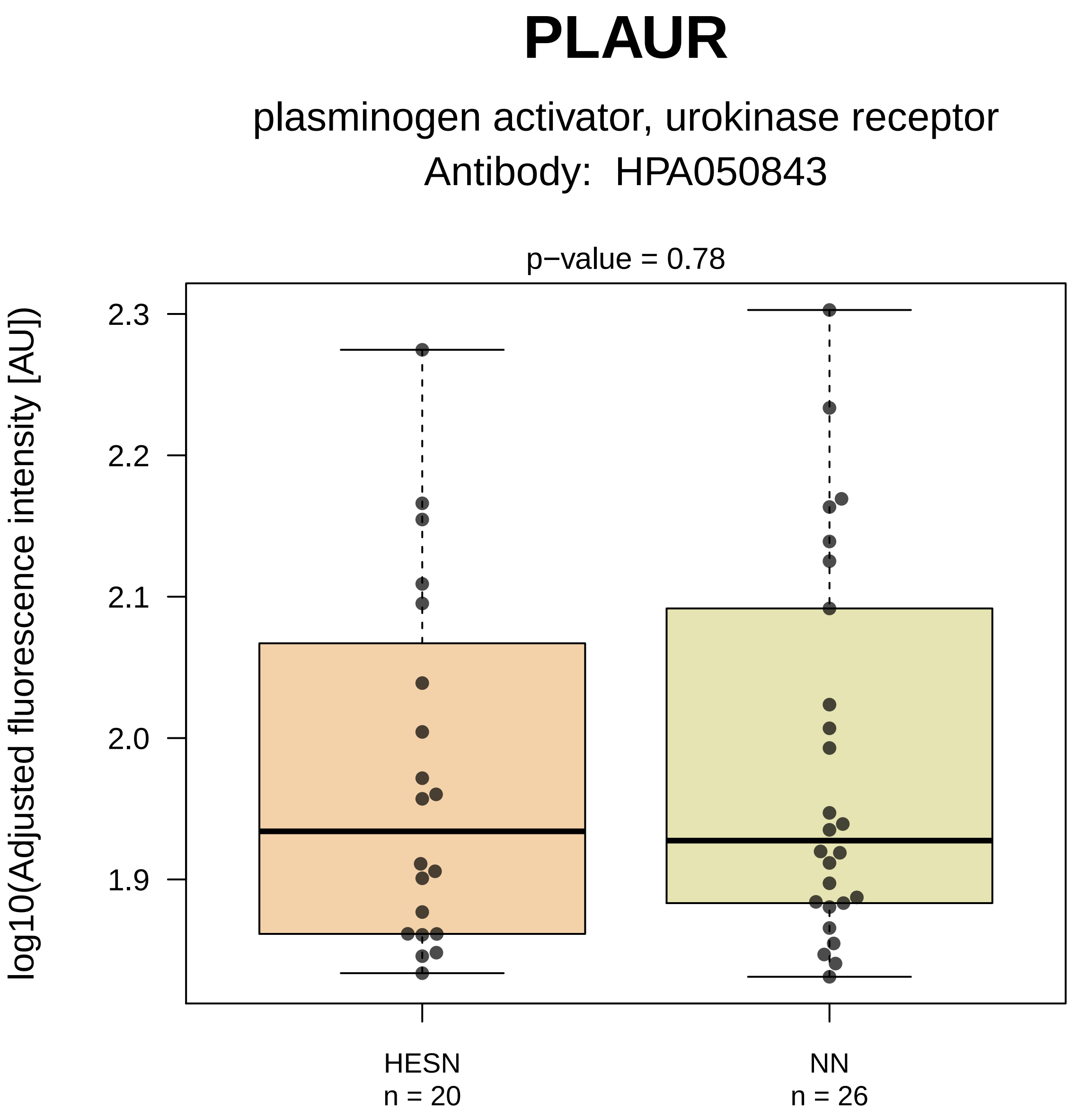

PLAUR
plasminogen activator, urokinase receptor
Antibody: HPA050843
p−value = 0.78
log10(Adjusted fluorescence intensity [AU])
2.3
2.2
2.1
2.0
1.9
HESN
n = 20
NN
n = 26

KRT5

keratin 5

Antibody: HPA059479

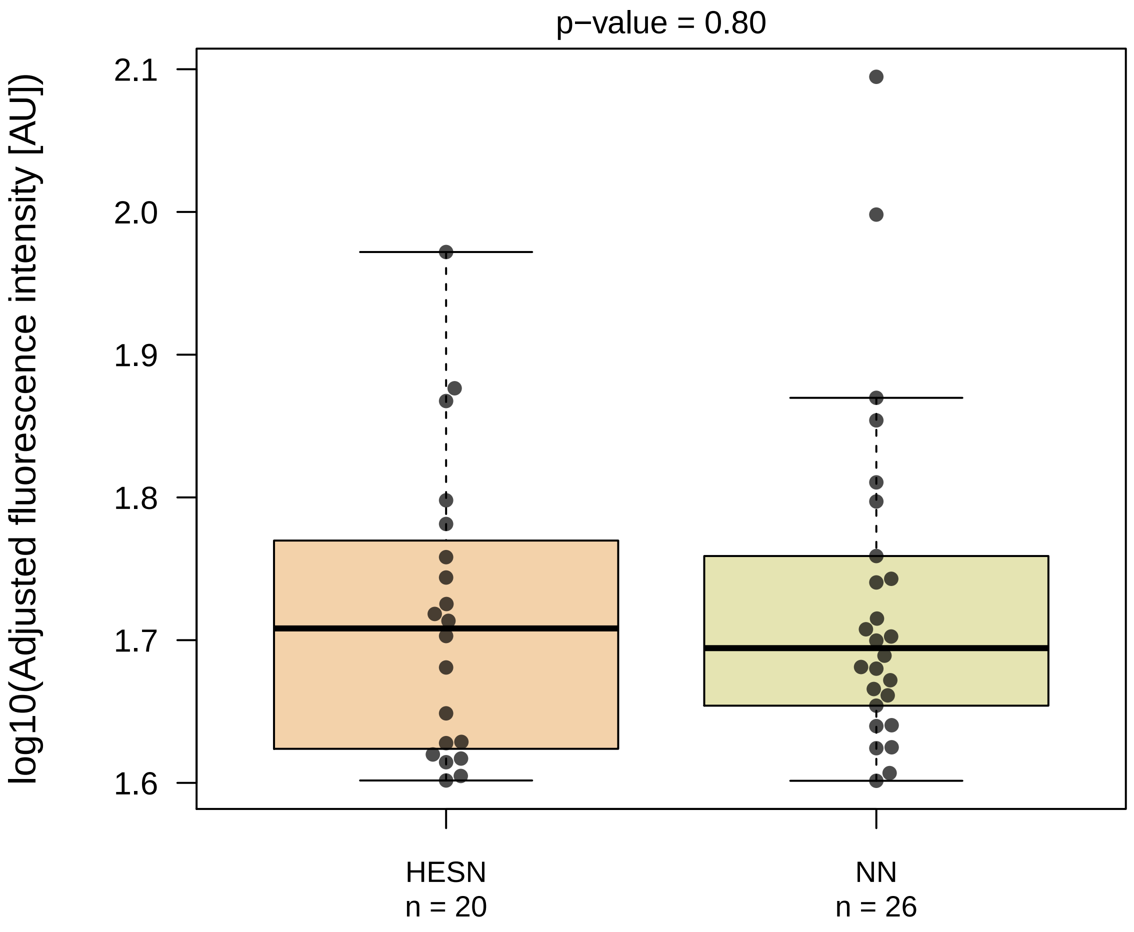

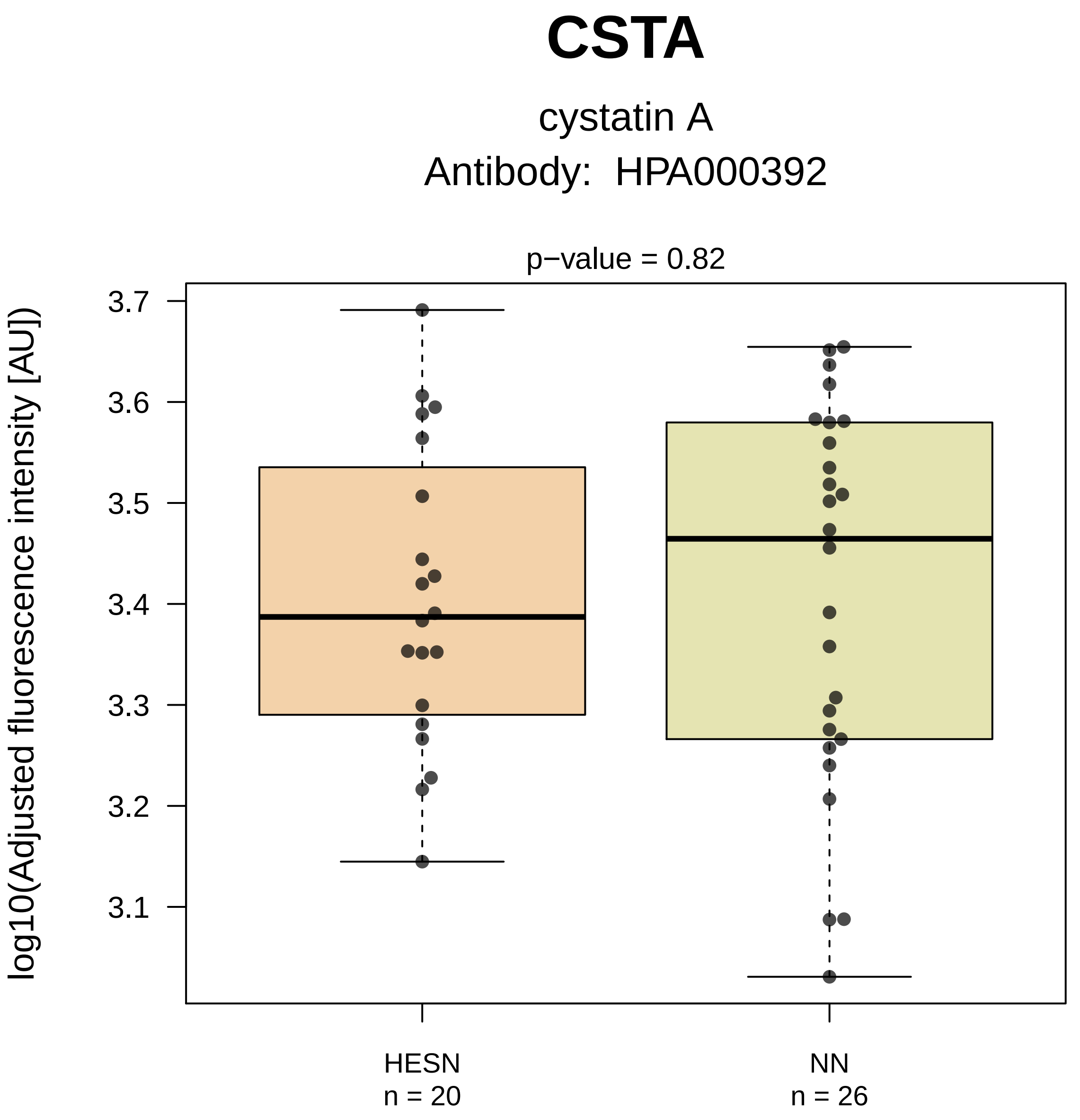
CSTA
cystatin A
Antibody: HPA000392
p−value = 0.82
log10(Adjusted fluorescence intensity [AU])
3.1
3.2
3.3
3.4
3.5
3.6
3.7
HESN
n = 20
NN
n = 26

DEFA5

defensin alpha 5

Antibody: HPA015775

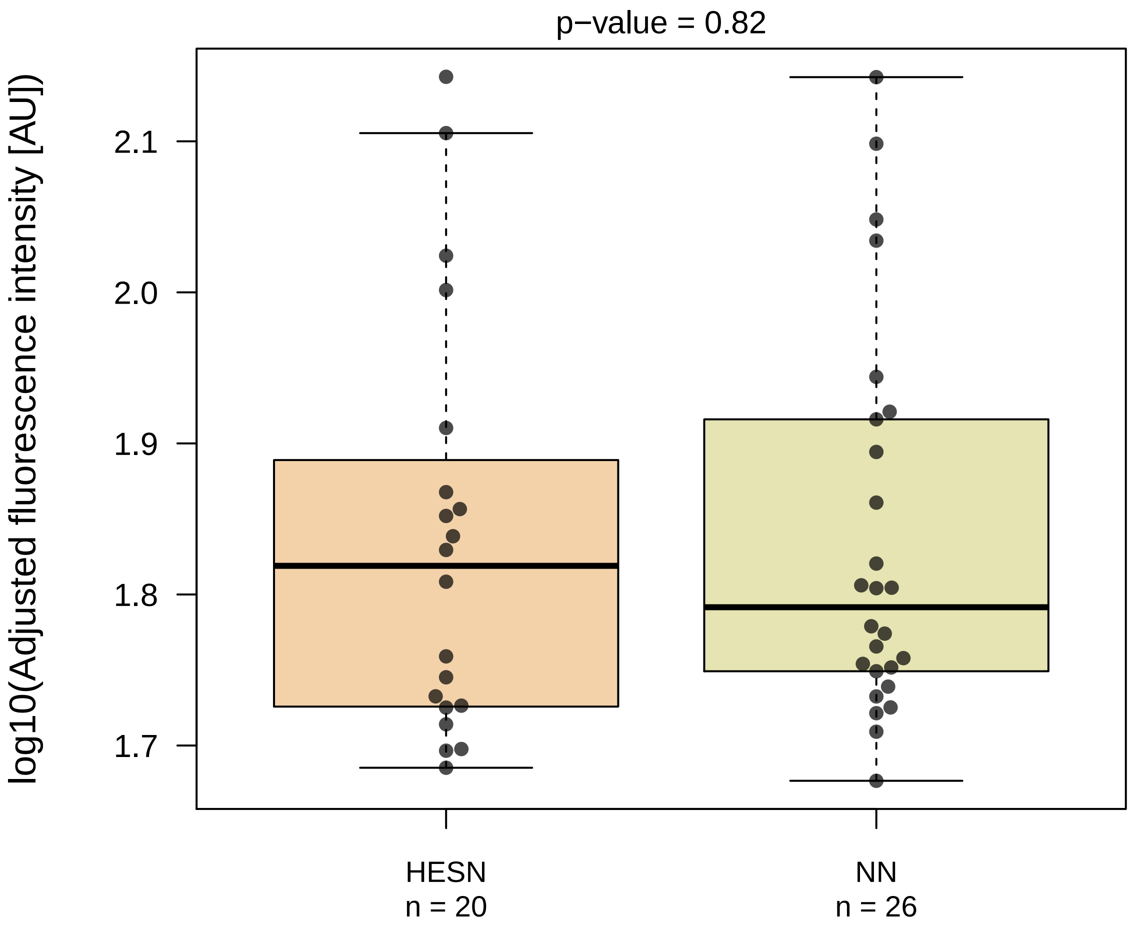

CAPN1

calpain 1

Antibody: HPA005992

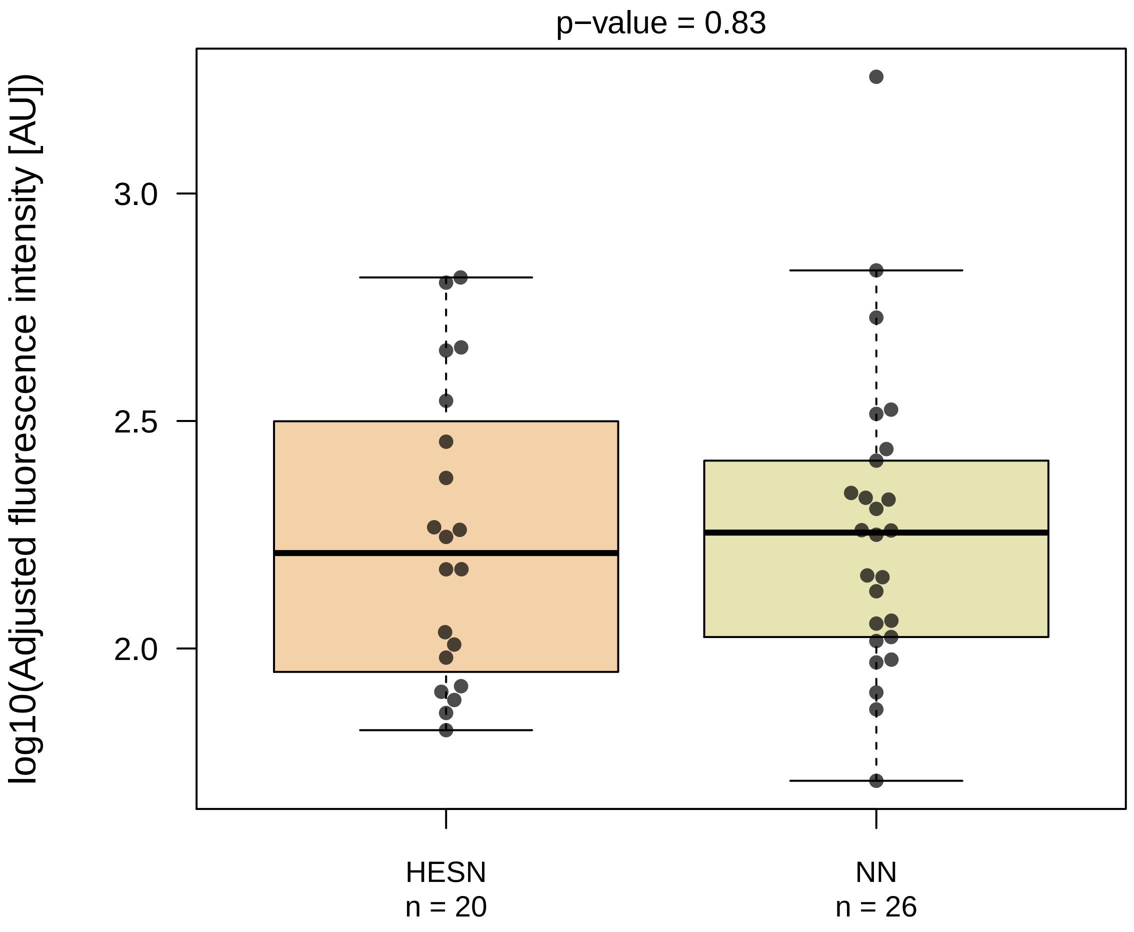

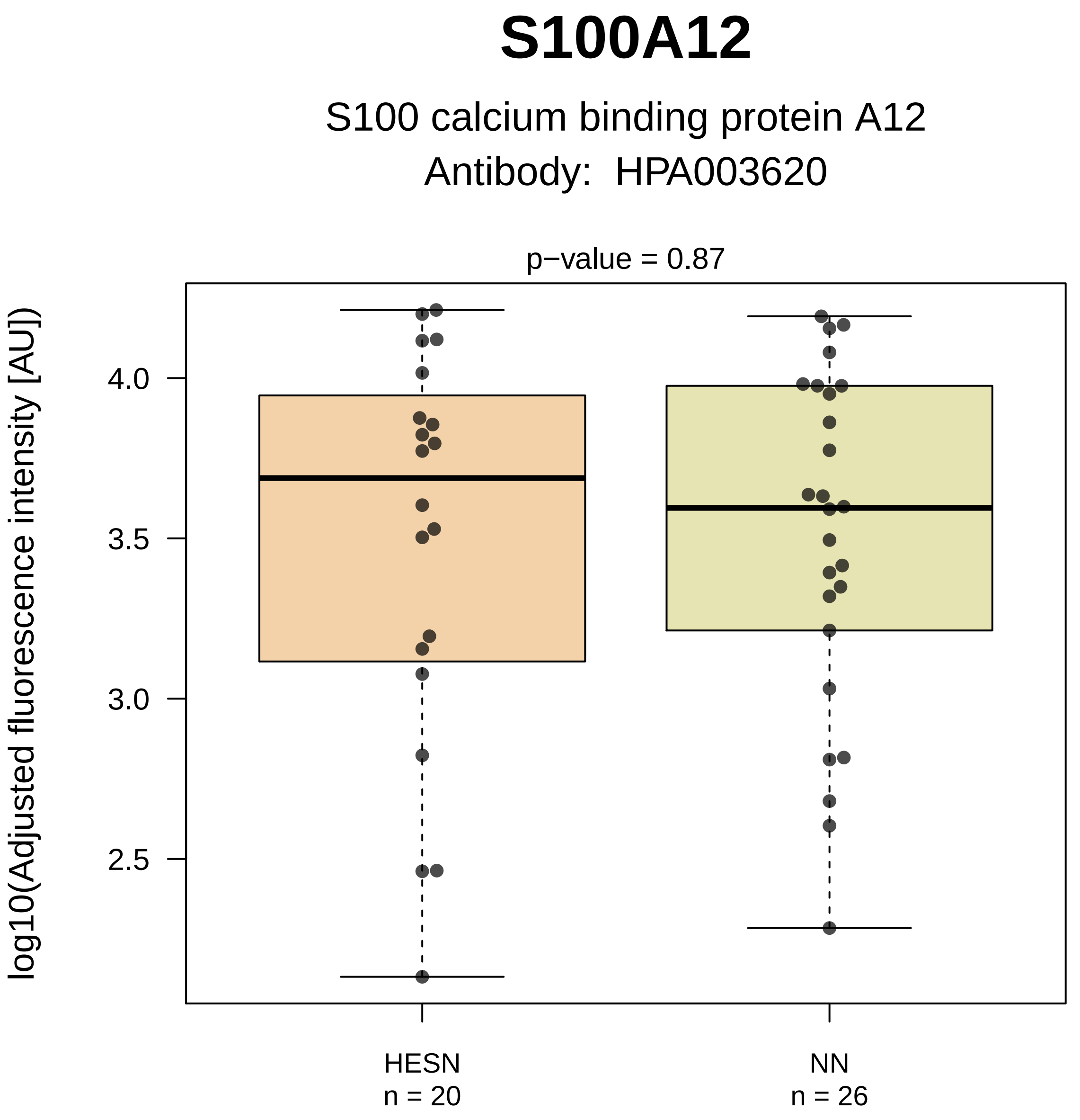
S100A12
S100 calcium binding protein A12
Antibody: HPA003620
p−value = 0.87
log10(Adjusted fluorescence intensity [AU])
4.0
3.5
3.0
2.5
HESN
n = 20
NN
n = 26

ANXA3

annexin A3

Antibody: HPA013398

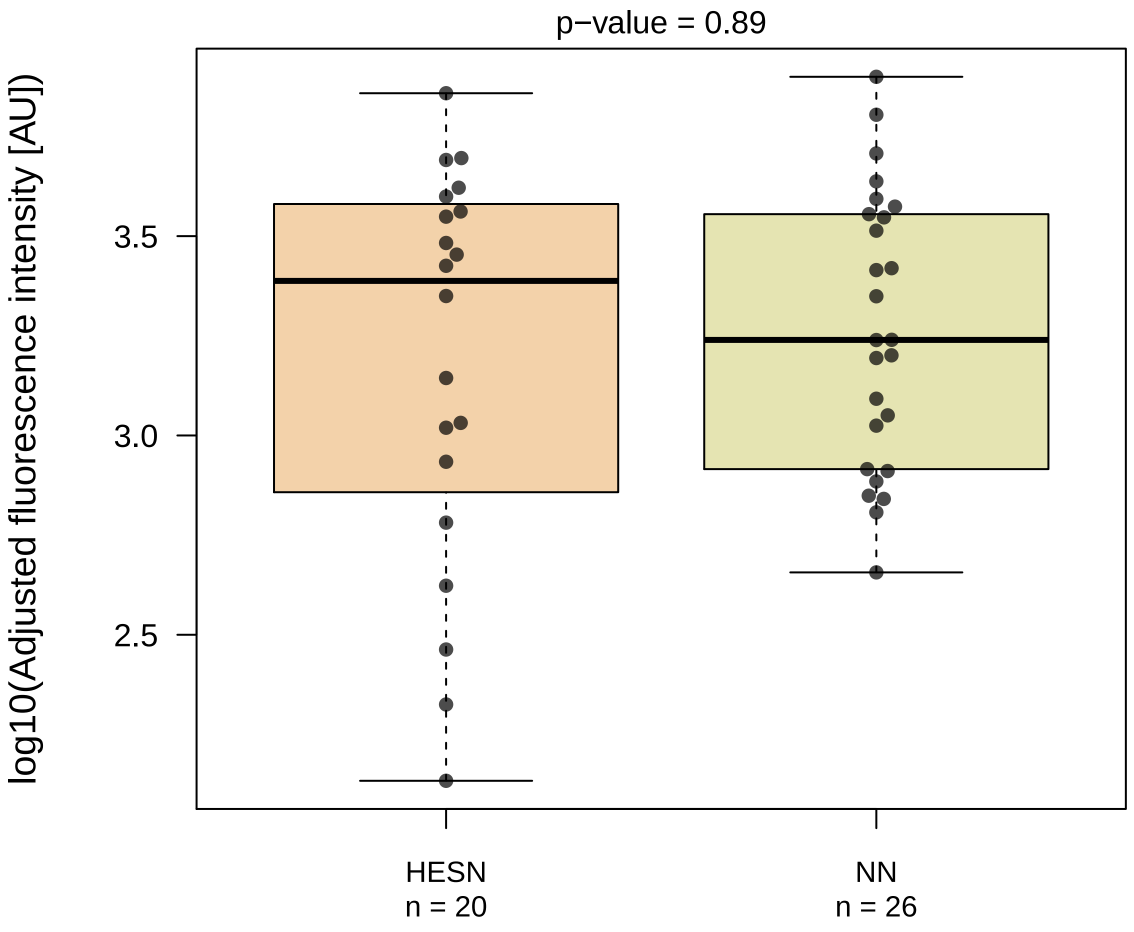

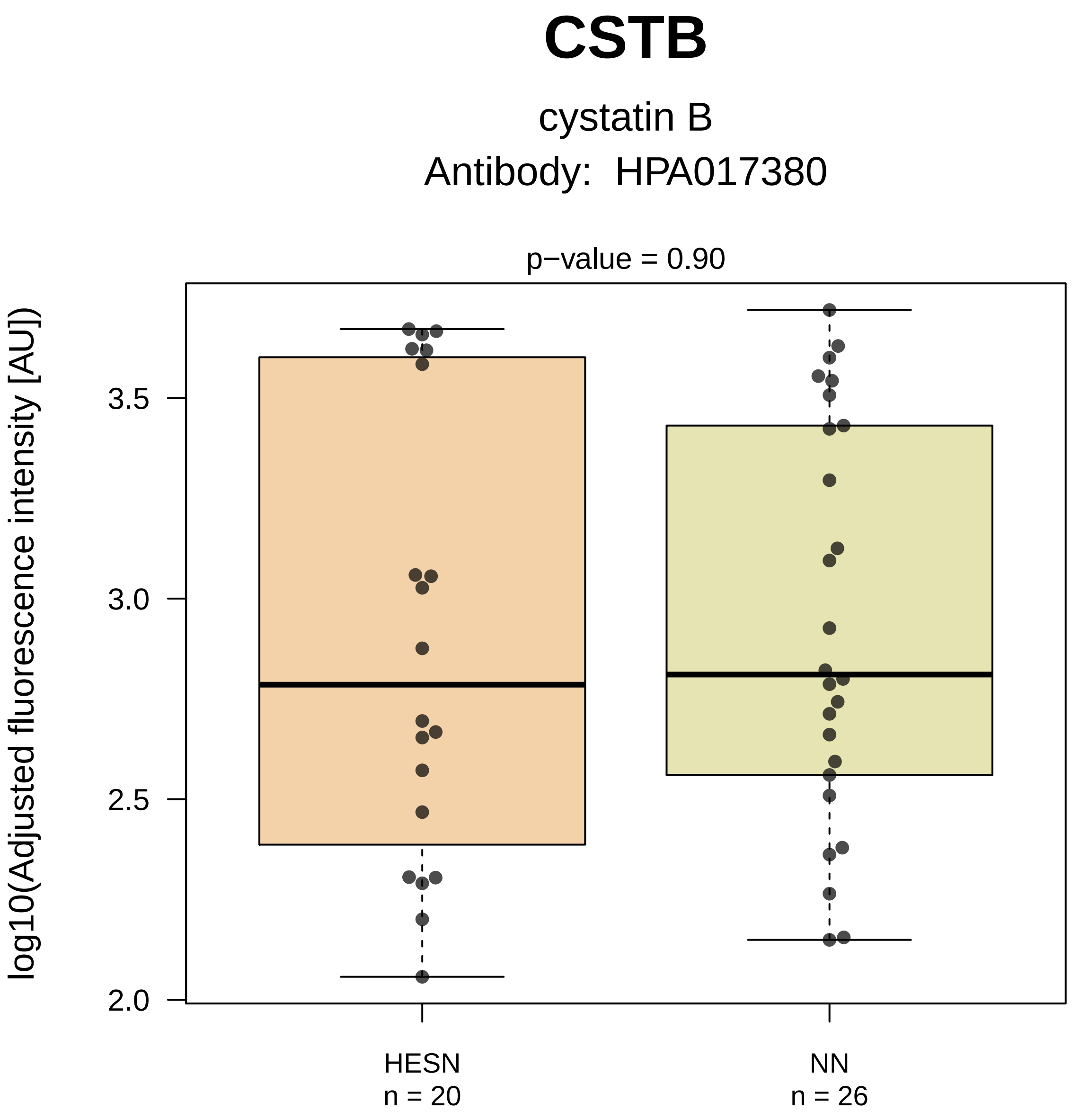

CSTB
cystatin B
Antibody: HPA017380
p−value = 0.90
log10(Adjusted fluorescence intensity [AU])
3.5
3.0
2.5
2.0
HESN
n = 20
NN
n = 26

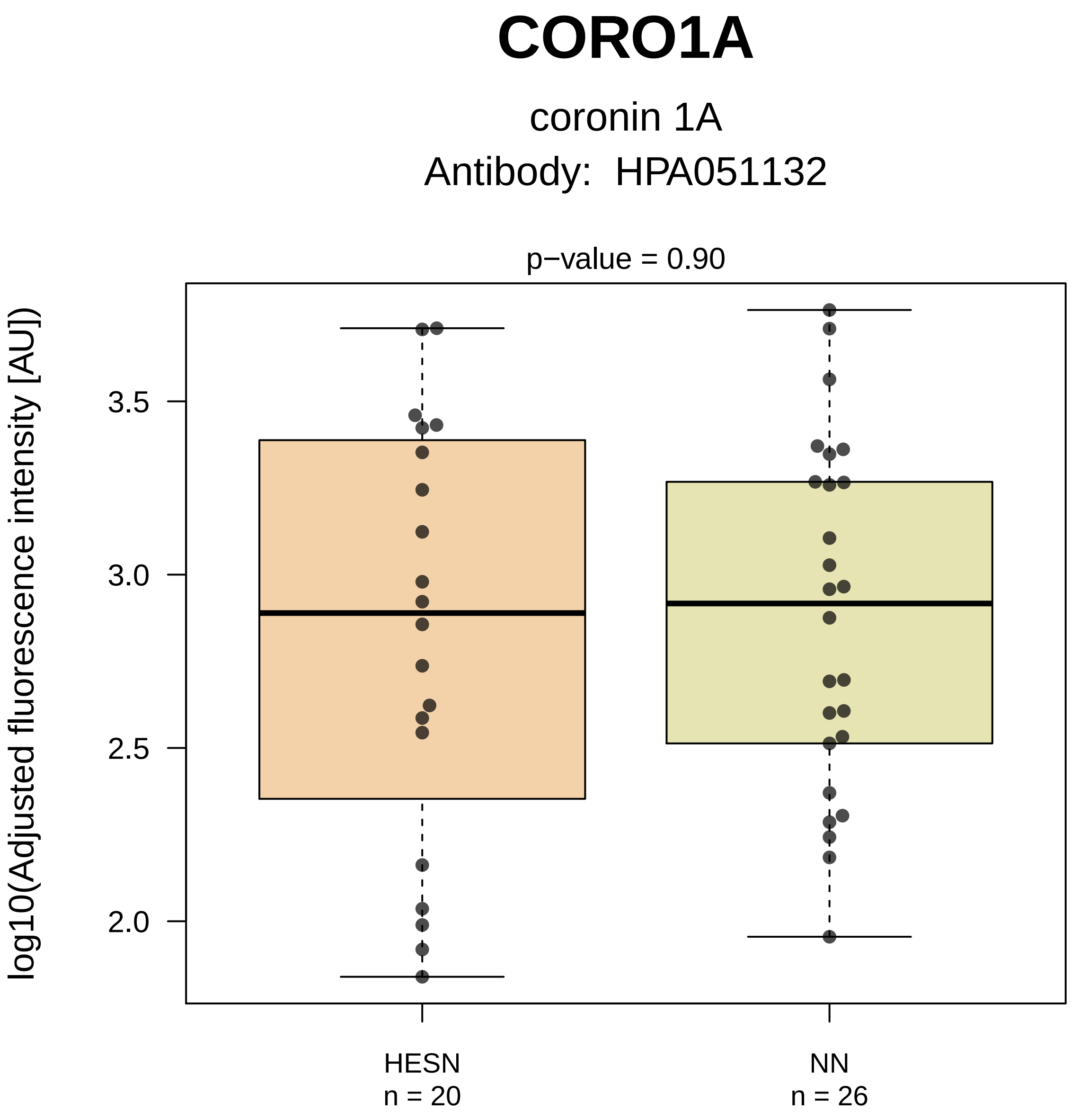
CORO1A
coronin 1A
Antibody: HPA051132
p−value = 0.90
log10(Adjusted fluorescence intensity [AU])
3.5
3.0
2.5
2.0
HESN
n = 20
NN
n = 26

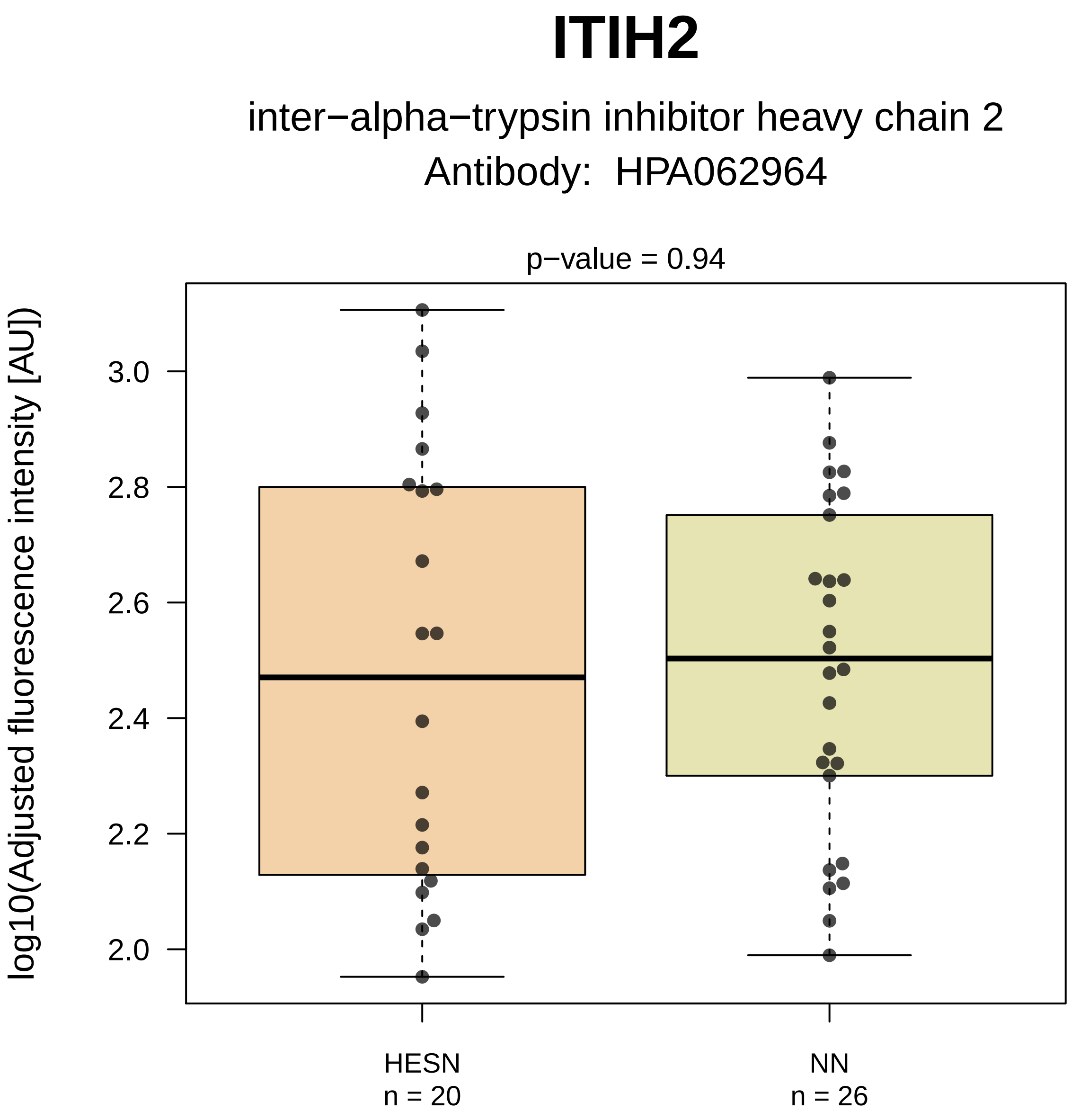
ITIH2
inter−alpha−trypsin inhibitor heavy chain 2
Antibody: HPA062964
p−value = 0.94
log10(Adjusted fluorescence intensity [AU])
3.0
2.8
2.6
2.4
2.2
2.0
HESN
n = 20
NN
n = 26

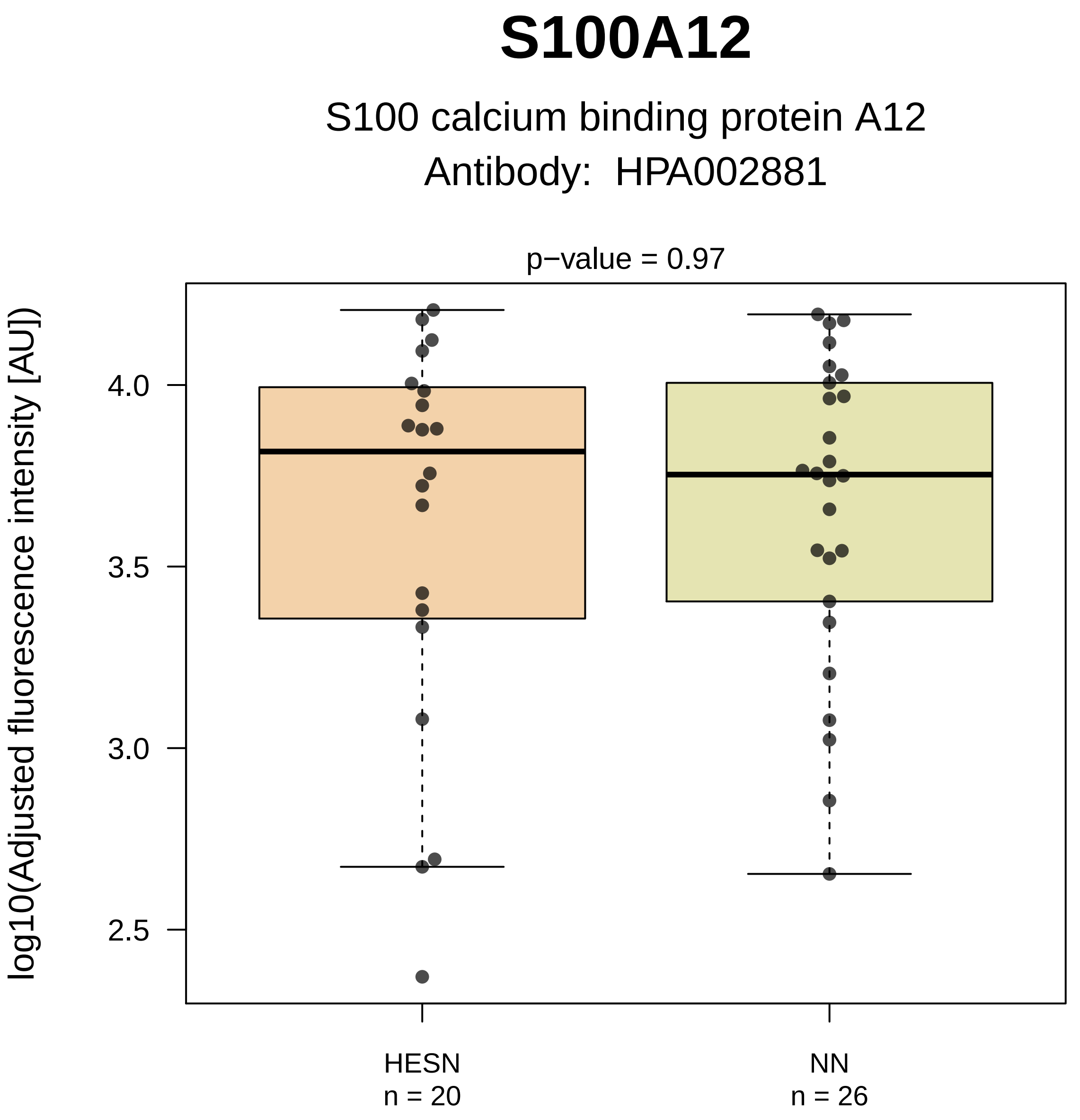

S100A12
S100 calcium binding protein A12
Antibody: HPA002881
p−value = 0.97
log10(Adjusted fluorescence intensity [AU])
4.0
3.5
3.0
2.5
HESN
n = 20
NN
n = 26

STS

steroid sulfatase

Antibody: HPA002904

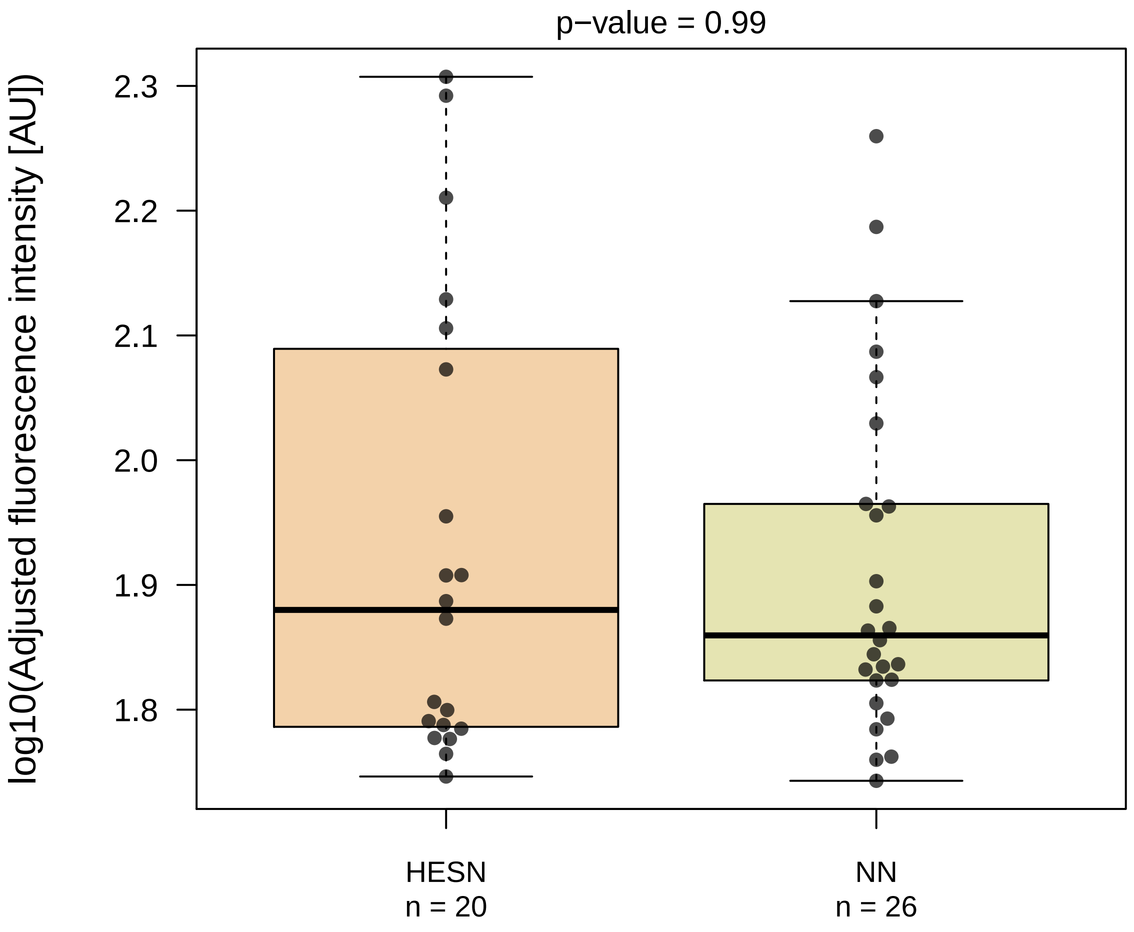

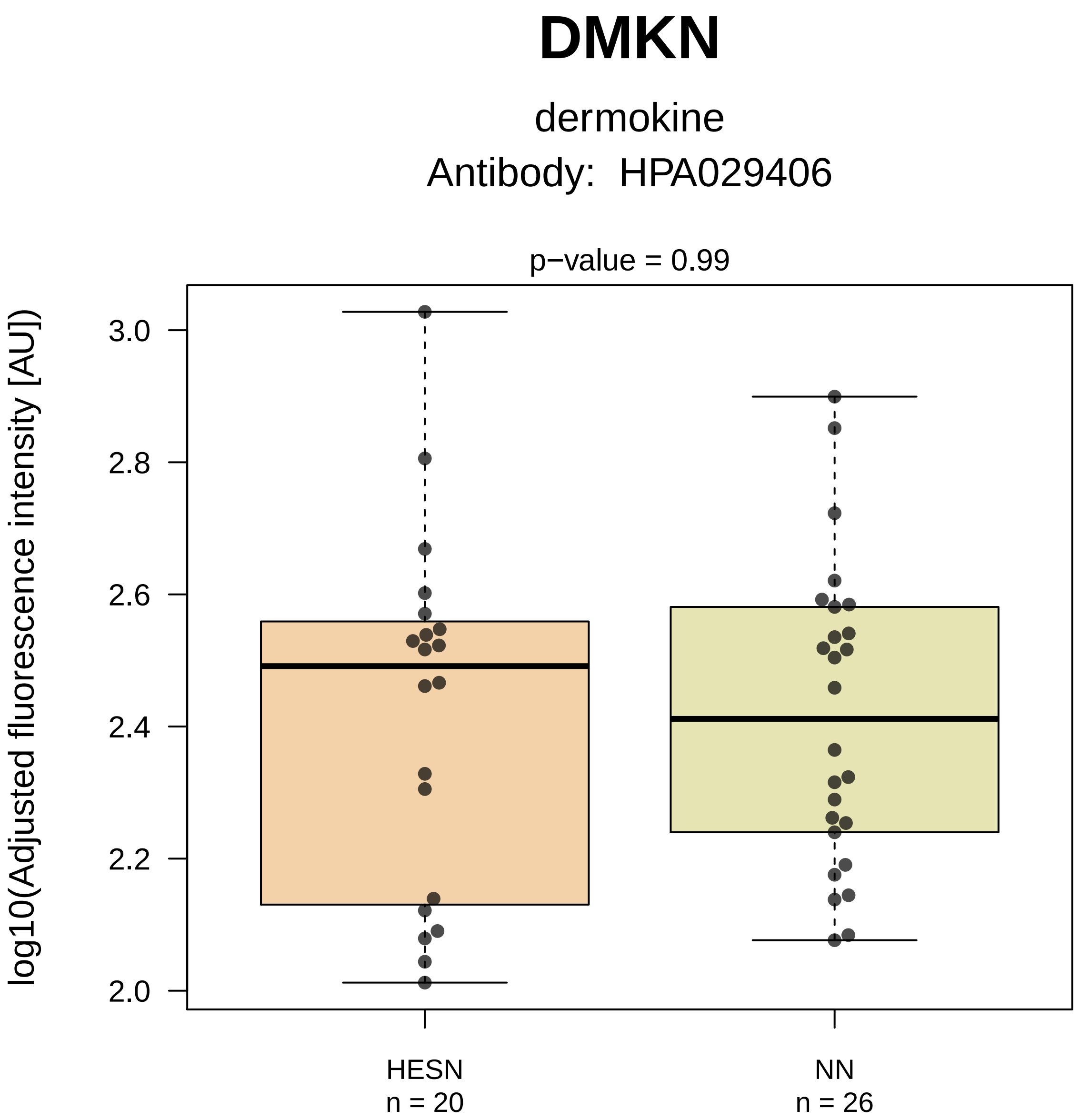
DMKN
dermokine
Antibody: HPA029406
p−value = 0.99
log10(Adjusted fluorescence intensity [AU])
3.0
2.8
2.6
2.4
2.2
2.0
HESN
n = 20
NN
n = 26

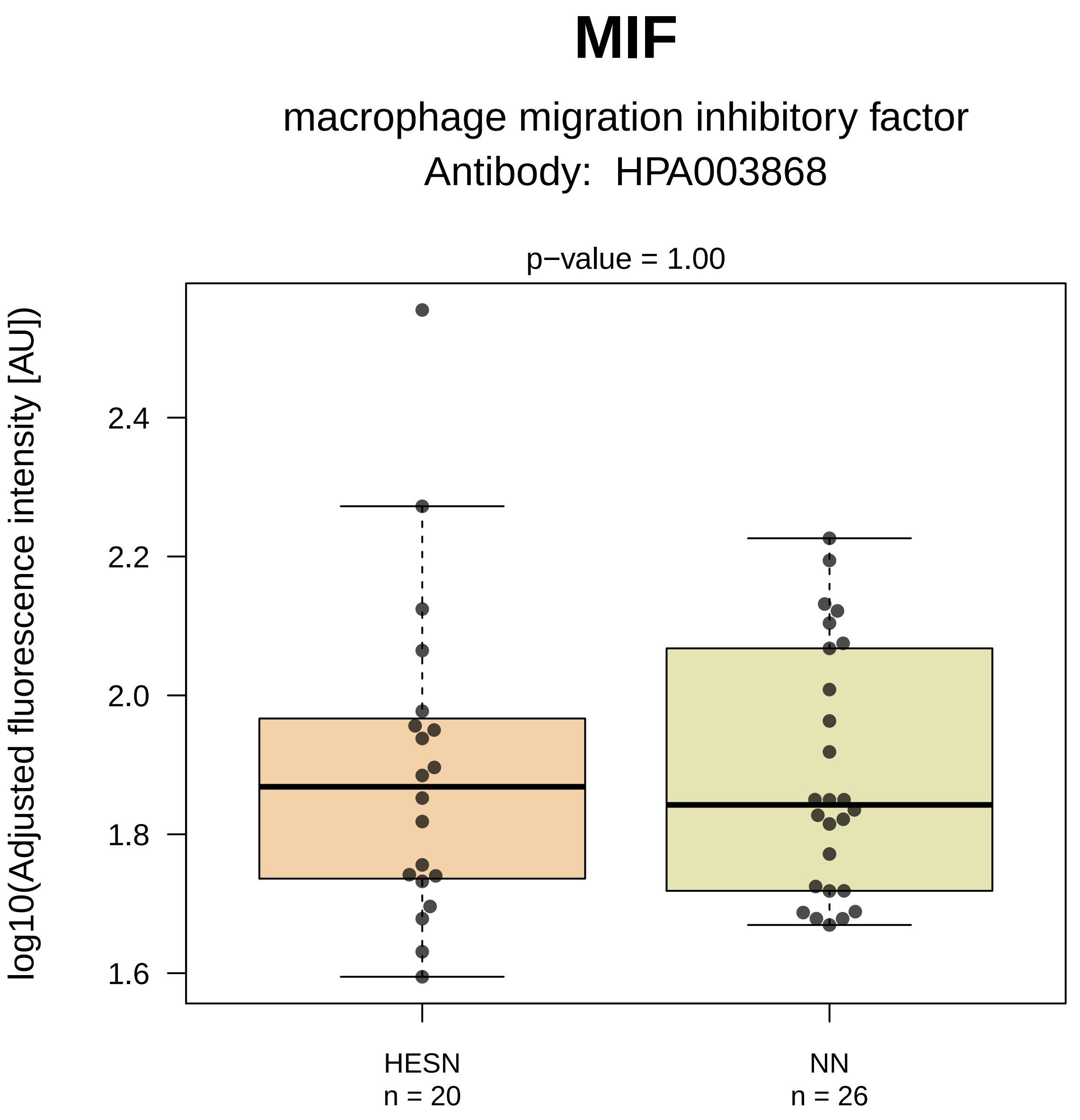

MIF
macrophage migration inhibitory factor
Antibody: HPA003868
p−value = 1.00
log10(Adjusted fluorescence intensity [AU])
2.4
2.2
2.0
1.8
1.6
HESN
n = 20
NN
n = 26
