# Supplementary material for: HIV-Exposed Seronegative Sex Workers Express Low T-Cell Activation and an Intact Ectocervical Tissue Microenvironment
Source: Vaccines (Basel). 2021 Mar 4;9(3):217. doi: 10.3390/vaccines9030217 (PMC7998094; doi:10.3390/vaccines9030217)
Supplement: Supplementary file 1 [file vaccines-09-00217-s001.zip › vaccines-1102333-new-supplementary/New Suppl.Tbl.1.pdf]

**Table S1.**	**Gene description**		
	Gene description	Uniprot ID	Antibody
AHSG	alpha 2-HS glycoprotein	P02765	HPA001524
ANXA1	annexin A1	P04083	HPA011918
ANXA3	annexin A3	P12429	HPA013431, HPA013398
BCAP31	B cell receptor associated protein 31	P51572	HPA003906
C4BPA	complement component 4 binding protein alpha	P04003	HPA001845
CAPN1	calpain 1	P07384	HPA005992
CAPN14	calpain 14	A8MX76	HPA067311
CAPNS1	calpain small subunit 1	P04632	HPA006872
CD5L	CD5 molecule like	O43866	HPA026432
CFH	complement factor H	P08603	HPA049176
COL1A2	collagen type I alpha 2 chain	P08123	HPA068464
CORO1A	coronin 1A	P31146	HPA051132
CRNN	cornulin	Q9UBG3	HPA024343
CSTA	cystatin A	P01040	HPA000392, HPA001031
CSTB	cystatin B	P04080	HPA058557, HPA017380
CTSG	cathepsin G	P08311	HPA047737
CXCL10	C-X-C motif chemokine ligand 10	P02778	HPA029858
DEFA1; DEFA1B; DEFA3	defensin alpha 1;defensin alpha 1B;defensin alpha 3	P59665;P59666	HPA044693
DEFA5	defensin alpha 5	Q01523	HPA015775
DMKN	dermokine	Q6E0U4	HPA029406
DSC2	desmocollin 2	Q02487	HPA012615
DSG1	desmoglein 1	Q02413	HPA022128
DSG3	desmoglein 3	P32926	HPA056863
ELANE	elastase, neutrophil expressed	P08246	HPA066836
F11R	F11 receptor	Q9Y624	HPA061700
FGA	fibrinogen alpha chain	P02671	HPA064755, HPA051370
FLNA	filamin A	P21333	HPA002925, HPA001115
GPX3	glutathione peroxidase 3	P22352	HPA071520, HPA062579
IFNG	interferon gamma	P01579	HPA049525
IL36G	interleukin 36 gamma	Q9NZH8	HPA055042
ITIH2	inter-alpha-trypsin inhibitor heavy chain 2	P19823	HPA059150, HPA062964

KLK11	kallikrein related peptidase 11	Q9UBX7	HPA038938
KNG1	kininogen 1	P01042	HPA001616
KRT1	keratin 1	P04264	HPA017917, HPA019797, HPA062908
KRT13	keratin 13	P13646	HPA030877
KRT14	keratin 14	P02533	HPA023040
KRT18	keratin 18	P05783	HPA001605
KRT4	keratin 4	P19013	HPA034881
KRT5	keratin 5	P13647	HPA059479
LDHA	lactate dehydrogenase A	P00338	HPA075026
LGMN	legumain	Q99538	HPA000799, HPA001426
LOR	loricrin	P23490	HPA076123
LRG1	leucine rich alpha-2-glycoprotein 1	P02750	HPA001888
LSP1	lymphocyte-specific protein 1	P33241	HPA019693
LYZ	lysozyme	P61626	HPA048284
MIF	macrophage migration inhibitory factor	P14174	HPA003868
MMP8	matrix metallopeptidase 8	P22894	HPA022935
MMP9	matrix metallopeptidase 9	P14780	HPA063909
MSN	moesin	P26038	HPA011135
MUC16	mucin 16, cell surface associated	Q8WXI7	HPA065600
MUC5AC	mucin 5AC, oligomeric mucus/gel-forming	P98088	HPA040615
MYH9	myosin heavy chain 9	P35579	HPA001644
NCF2	neutrophil cytosolic factor 2	P19878	HPA002327, HPA006040
PI3	peptidase inhibitor 3	P19957	HPA017737
PKP1	plakophilin 1	Q13835	HPA027221
PLAUR	plasminogen activator, urokinase receptor	Q03405	HPA050843
RPTN	repetin	Q6XPR3	HPA030485
S100A12	S100 calcium binding protein A12	P80511	HPA002881, HPA003620
S100A2	S100 calcium binding protein A2	P29034	HPA034651
S100A6	S100 calcium binding protein A6	P06703	HPA008060
S100P	S100 calcium binding protein P	P25815	HPA019502
SERPINA1	serpin family A member 1	P01009	HPA001291
SERPINA3	serpin family A member 3	P01011	HPA000893
SERPINA6	serpin family A member 6	P08185	HPA017864
SERPINB1	serpin family B member 1	P30740	HPA018871
SERPINB5	serpin family B member 5	P36952	HPA019025, HPA019132
SERPINB7	serpin family B member 7	O75635	HPA024200

SERPING1	serpin family G member 1	P05155	HPA048738
SPINK5	serine peptidase inhibitor, Kazal type 5	Q9NQ38	HPA011351, HPA009067
SPRR3	small proline rich protein 3	Q9UBC9	HPA024330, HPA044467
STS	steroid sulfatase	P08842	HPA002904
TACSTD2	tumor associated calcium signal transducer 2	P09758	HPA055067, HPA043104
TMPRSS11E	transmembrane serine protease 11E	Q9UL52	HPA051062
TXNDC17	thioredoxin domain containing 17	Q9BRA2	HPA022931

Protein targets were selected based on their presence in genital fluids and with associations to HIV resistance and inflammation [20], as well as identified as genes or proteins in studies on the effect of sex hormones and inflammation in the female genital tract [21-25].
