# Supplementary material for: HIV-Exposed Seronegative Sex Workers Express Low T-Cell Activation and an Intact Ectocervical Tissue Microenvironment
Source: Vaccines (Basel). 2021 Mar 4;9(3):217. doi: 10.3390/vaccines9030217 (PMC7998094; doi:10.3390/vaccines9030217)
Supplement: Supplementary file 1 [file vaccines-09-00217-s001.zip › vaccines-1102333-new-supplementary/New Suppl.Tbl.2.pdf]

Table S2. Clinical characterization of study subjects (CMC analyses)

	HESN (n=20)	NN (n=11)	p-value
	Median or number (range or %)	Median or number (range or %)	
Age (years)	37 (32-48)	33 (21-47)	ns
Years since last pregnancy[a]	7 (1-19)	8 (5-22)	ns
Progesterone levels (ng/mL)[b]	3.6 (0.6-16.9)	3.5 (0.1-16.4)	ns
BV[c]			
Negative (0-3)	9 (45%)	8 (73%)	ns
Intermediate (4-6)	6 (30%)	1 (9%)	ns
Positive (7-10)	7 (25%)	1 (9%)	ns
Yeast infection	0 (0%)	1(9%)	ns
No. of clients last 7 days	5 (2-50)	3.5 (1-18)	ns
No. of unprotected sex acts last 7 days[d]	0 (0-6)	0 (0-1)	ns
Vaginal douching[e]	10 (50%)	4 (36%)	ns

Clinical characteristics at time of enrolment for the participants from whom CMC samples were available at the first sample time point.
ns= non-significant (p<0,05), Mann-Whitney *U* test and Fisher´s exact test.
[a]Years since last pregnancy, including abortions; Data not available from 4 HESN and 1 NN participants, respectively.
[b]Progesterone levels at the time of first sample time point.

[c]BV: Bacterial vaginosis (Nugent's score, 1-10; Negative 0-3; Intermediate 4-6; Positive: 7-10), data not available from 1 NN participant
[d]Calculated based on self-reported use of condom with clients
[e]Any douching performed by inserting water, or water and soap, in the vagina since last study visit (approximately 2 weeks)
