# Supplementary figures and images for: HIV-Exposed Seronegative Sex Workers Express Low T-Cell Activation and an Intact Ectocervical Tissue Microenvironment
Source: Vaccines (Basel). 2021 Mar 4;9(3):217. doi: 10.3390/vaccines9030217 (PMC7998094; doi:10.3390/vaccines9030217)

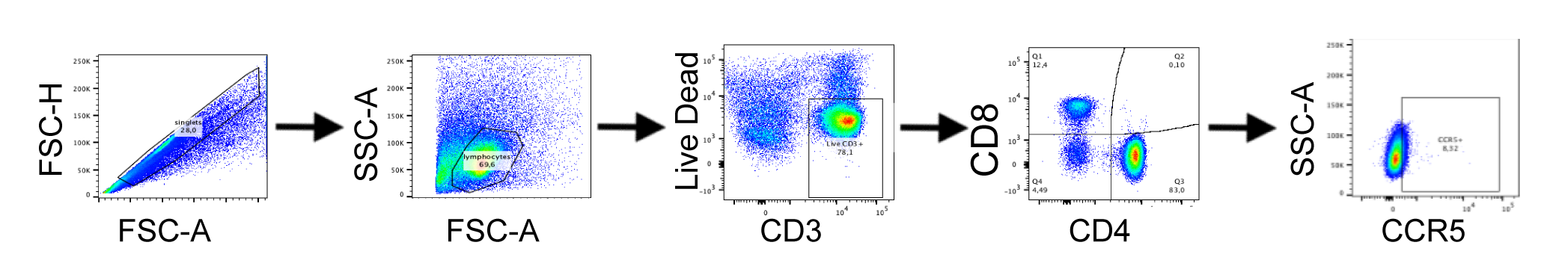

Supplement: Supplementary file 1 [file vaccines-09-00217-s001.zip › vaccines-1102333-new-supplementary/Fig S1 gating.tif]

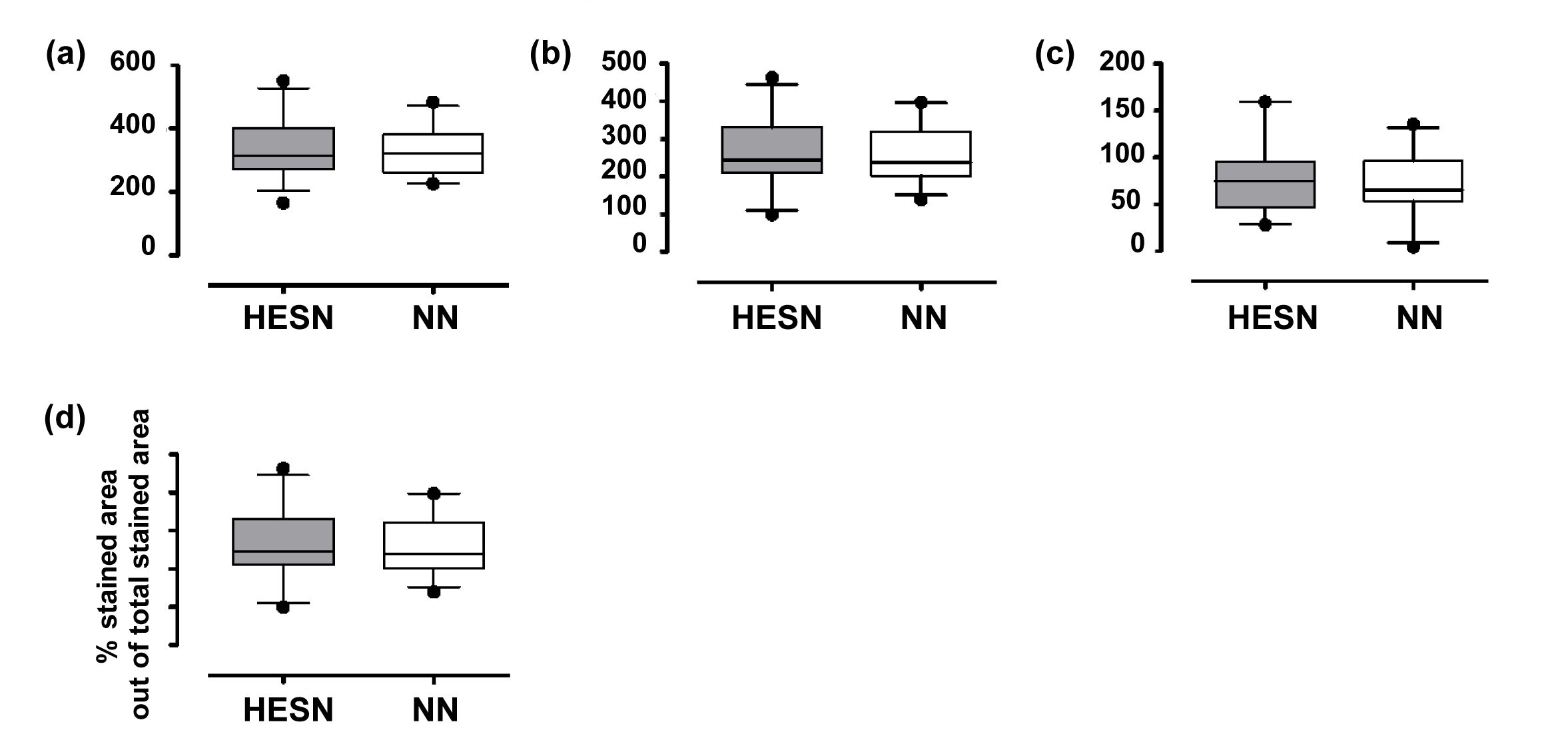

Supplement: Supplementary file 1 [file vaccines-09-00217-s001.zip › vaccines-1102333-new-supplementary/Fig S4 staining.tif]
